# Crystallographic Detection of the Spin State in Fe^III^ Complexes

**DOI:** 10.1021/acs.cgd.2c00468

**Published:** 2022-10-17

**Authors:** Conor T. Kelly, Michael Griffin, Kane Esien, Solveig Felton, Helge Müller-Bunz, Grace G. Morgan

**Affiliations:** † School of Chemistry, University College Dublin, Belfield, Dublin 4D04 N2E5, Ireland; ‡ School of Mathematics and Physics, Queen’s University Belfast, BelfastBT7 1NN, United Kingdom

## Abstract

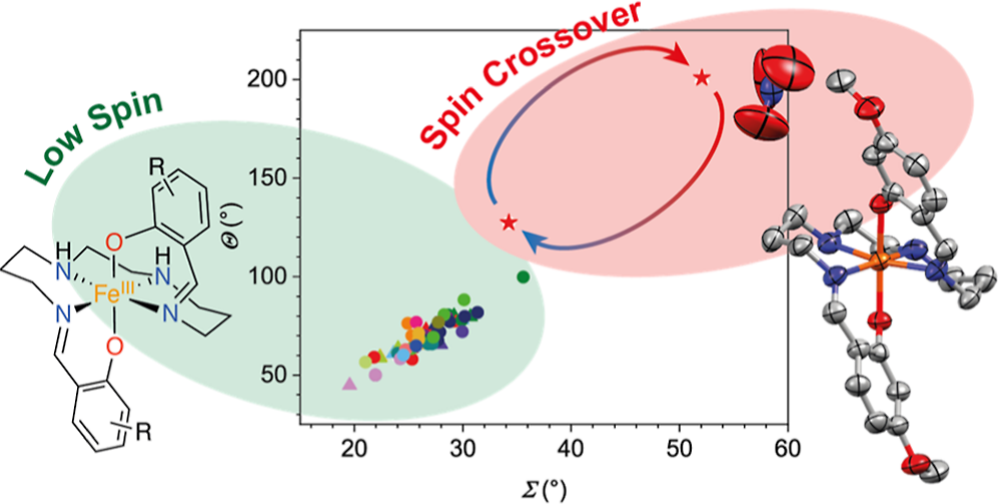

We report a single example of thermal spin crossover in a series of Fe^III^ complexes, [Fe^III^(R-sal_2_323)]^+^, which typically stabilize the low-spin (*S* = 1/2) state. Single-crystal X-ray diffraction analysis of 53 such complexes with varying “R” groups, charge-balancing anions, and/or lattice solvation confirms bond lengths in line with an *S* = 1/2 ground state, with only the [Fe^III^(4-OMe-sal_2_323)]NO_3_ complex (**1a**) exhibiting longer bond lengths associated with a percentage of the spin sextet form at room temperature. Structural distortion parameters are investigated for the series. A magnetic susceptibility measurement of **1a** reveals a gradual, incomplete transition, with *T*_1/2_ = 265 K in the solid state, while Evans method NMR reveals that the sample persists in the low-spin form in solution at room temperature. Computational analysis of the spin state preferences for the cations [Fe^III^(4-OMe-sal_2_323)]^+^ and [Fe^III^(sal_2_323)]^+^ confirmed the energetic preference for the spin doublet form in both, and the thermal spin crossover in complex **1a** is therefore attributed to perturbation of the crystal packing on warming.

## Abstract 2

A single example of thermal spin state switching is detected from more than 50 low-spin [Fe^III^(R-sal_2_323)]X complexes where crystal engineering is used to vary the ligand substituent R and charge balancing counterion X. Computational analysis indicates that the spin doublet form is more stable, and the spin crossover is therefore attributed to thermally induced perturbation of the crystal packing.

## Introduction

Transition metal complexes with a d^4^–d^7^ electronic configuration which undergo spin crossover offer a set of molecular materials for the manufacture of many prospective devices.^1^ In response to an external stimulus (most often heat), switching can be achieved between the spin state of maximum multiplicity (high spin, HS) and minimum multiplicity (low spin, LS). This phenomenon is most well reported for Fe^II^ where transitions between a paramagnetic *S* = 2 HS and diamagnetic *S* = 0 LS state can be observed.^2^ There are, however, now many examples of spin crossover with other metal centers such as Fe^III^, Mn^III^, Co^II^, and Cr^III^.^3,4^ Crystallographic measurements represent one of the most useful tools for the detection of spin crossover due to changes in metal-to-ligand bond lengths upon changing the spin state. These changes in metal-to-ligand bond length can have drastic effects on intermolecular interactions and packing effects and sometimes result in changes in symmetry.^5−7^ As such, crystallography is a powerful tool in the determination of the spin state, enabling the analysis of the factors that influence the spin state and/or spin crossover.

Chelating Schiff base ligands have offered a suitable electronic and geometric environment for a wealth of complexes that undergo spin crossover, with a number of different transition metals.^2^ With Fe^III^, these have been comprehensively reviewed recently by Harding et al.^3^ In the case of hexadentate chelates, the combination of two substituted salicylaldehydes with a tetraamine backbone has yielded a number of different ligands which support spin crossover. Tweedle and Wilson discovered that [Fe^III^(R-sal(trien)]X (which we will refer to as [Fe^III^(R-sal_2_222)]X to denote the alkylene chain connectivity in the tetraamine backbone) complexes showed thermal spin crossover in Fe^III^ in 1976, Figure 1.^8^ Later, in 2010, we showed that spin crossover was also possible in Fe^III^ complexes with an additional −CH_2_– group in the central alkylene linker of the backbone in the complex [Fe^III^(4,6-diOMe-sal_2_232)]ClO_4_.^9^ Complexes with additional −CH_2_– groups in the alkylene linkers, such as [Fe^III^(R-sal_2_333)]X, have also recently been shown to support both the HS and LS states, although switching between the two has not yet been observed.^10^ A handful of complexes of the [Fe^III^(R-sal_2_323)]X form has been reported in the literature,^11−16^ and in each case, the complexes supported the LS state. Hayami et al. suggested that the stability of the LS state is because the ligand field is stronger in the [Fe^III^(R-sal_2_323)]X complexes compared to that in the [Fe^III^(R-sal_2_333)]X analogues because the five-membered middle chelate ring may support conjugation of the π-electrons from both sides of the ligand.^12^

**Figure 1:**
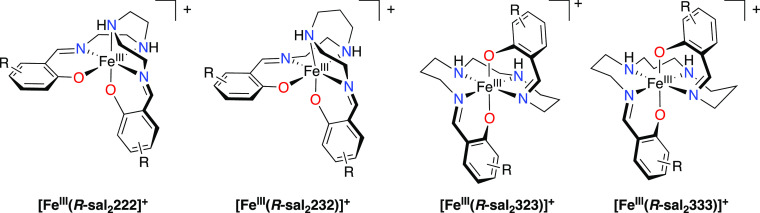
Fe^III^ complexes with hexadentate Schiff base ligands with a N_4_O_2_^2–^ coordination sphere. Relevant nomenclature is indicated beneath each molecule, whereby the numbers refer to the lengths of the alkyl chains between each of the nitrogens of the tetraamine backbone (e.g., 232 refers to an ethylene, propylene, ethylene linkage).

These three sets of complexes show that an increase in the length of the tetraamine backbone, from 6 −CH_2_– groups (“222”) to 7 −CH_2_– groups (“232”) and 9 −CH_2_– groups (“333”), can be used to modify the relative stability of the HS and LS states and the nature of spin crossover. An anomaly lies with a tetraamine backbone with 8 −CH_2_– groups (“323”) where there are no examples of the stability of both the HS and LS states in the literature. The “323” ligand is well known for supporting thermal spin crossover in Mn^III^.^4,17,18^ Herein, we investigate the feasibility of thermal spin crossover in complexes of the form [Fe^III^(R-sal_2_323)]X, Figure 1.

## Results and Discussion

**Scheme 1:**
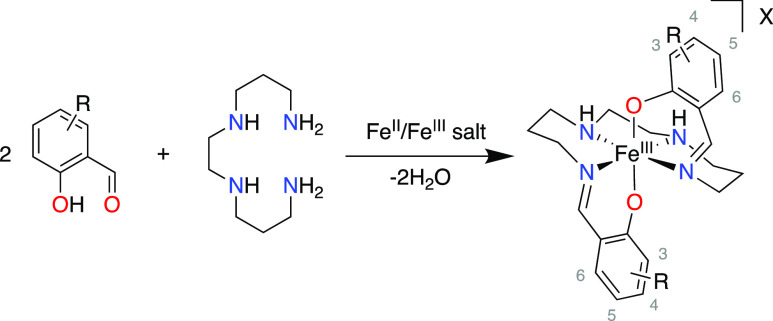
General Synthetic Approach for the Formation of Complex Families **1–17** The appropriate Fe^II^/Fe^III^ salt and exchange salt were used.

Synthesis of complex families **1–17** was achieved via a facile one-pot Schiff base condensation of a salicylaldehyde and 1,2-bis(3-aminopropylamino)ethane followed by complexation with an Fe^II^ (undergoes aerial oxidation to Fe^III^) or Fe^III^ salt. This results in the formation of complexes of the general form [Fe^III^(R-sal_2_323)]X, where “R-sal” refers to the respective substituted salicylaldehyde moiety, “323” refers to the tetra-amine backbone where the number denotes the alkylene connectivity between the nitrogen atoms (i.e., propylene, ethylene, propylene), and “X” refers to the charge balancing anion used, Scheme 1.

Modification of the “R” substituent on the salicylaldehyde moiety enabled analysis of the effect of using a variety of electron-donating and electron-withdrawing groups, Table 1. The spin doublet (*S* = 1/2) state was reliably stabilized as the ground state with the exception of [Fe^III^(4-OMe-sal_2_323)]NO_3_ (**1a**) which was the only example which exhibited thermal spin crossover behavior. Further details for each complex can be found in Table S1.1.

**Table 1:** Summary of All Complexes, **1–17**, Reported in This Work along with Their Respective Space Group, Distortion Parameters (vide infra), and Assigned Spin State^a^

complex	molecular formula	S.G.	*T* (K)^b^,^c^	Σ (°)^d^	Θ (°)^d^	spin state^e^
1a	[Fe^III^(4-OMe-sal_2_323)]NO_3_	*P*2_1_2_1_2	100	34.26	127.29	SCO
			293	52.06	200.78	
1a·S	[Fe^III^(4-OMe-sal_2_323)]NO_3_·0.75MeCN·0.25MeOH	*P*2_1_/*n*	100	24.13	58.94	LS
			293	25.22	63.53	
1b	[Fe^III^(4-OMe-sal_2_323)]PF_6_·0.45H_2_O	*P*2_1_/*c*	100	27.78	71.98	LS
1c	[Fe^III^(4-OMe-sal_2_323)]OTf·0.27H_2_O^f^	*P*2_1_/*c*	100	25.63	68.02	LS
1d	[Fe^III^(4-OMe-sal_2_323)]ClO_4_	*P*2_1_/*c*	100	21.83	59.15	LS
			200	27.34	73.46	
1e	[Fe^III^(4-OMe-sal_2_323)]BF_4_	*P*2_1_/*c*	293	26.62	72.86	LS
1f	[Fe^III^(4-OMe-sal_2_323)]SbF_6_·0.31H_2_O	*P*2_1_/*c*	100	27.79	71.75	LS
1g	[Fe^III^(4-OMe-sal_2_323)]I_3_	*P*2_1_/*c*	100	25.33	58.11	LS
1h	[Fe^III^(4-OMe-sal_2_323)]Cl·EtOH·0.25H_2_O	*P*2_1_/*c*	100	29.23	77.52	LS
2a	[Fe^III^(3-OMe-sal_2_323)]NO_3_	*Pccn*	100	35.59	99.98	LS
			293	29.20	80.03	LS
2b	[Fe^III^(3-OMe-sal_2_323)]BF_4_·H_2_O	*P*2_1_/*c*	100	28.65	80.74	LS
2c	[Fe^III^(3-OMe-sal_2_323)]PF_6_·H_2_O	*P*2_1_/*c*	293	28.27	78.45	LS
2d	[Fe^III^(3-OMe-sal_2_323)]FeCl_4_	*P*2_1_/*c*	150	30.71	79.62	LS^16^
2e	[Fe^III^(3-OMe-sal_2_323)]ClO_4_	*P*2_1_	173	29.9	78.11	LS^15^
3a	[Fe^III^(5-OMe-sal_2_323)]NO_3_	*P*2/*c*	100(I)	21.94	50.17	LS
			100(II)	24.24	58.49	
			293(I)	23.86	61.35	
			293(II)	29.92	73.33	
3b	[Fe^III^(5-OMe-sal_2_323)]BF_4_	*P*2/*c*	100(I)	28.51	79.56	LS
			100(II)	26.47	70.26	LS
4a	[Fe^III^(4,6-diOMe-sal_2_323)]NO_3_·MeOH	*P*2_1_/c	100	29.29	83.14	LS
			293	27.79	73.82	LS
4b	[Fe^III^(4,6-diOMe-sal_2_323)]BF_4_·0.5MeOH	*P*2_1_/*n*	100	29.11	79.30	LS
			293	27.26	70.69	LS
4c	[Fe^III^(4,6-diOMe-sal_2_323)]ClO_4_·0.5MeOH	*P*2_1_/*n*	100	28.51	77.47	LS
5a	[Fe^III^(3-OEt-sal_2_323)]PF_6_·EtOH	*P*2_1_/*n*	100	25.32	70.2	LS
5b	[Fe^III^(3-OEt-sal_2_323)]BF_4_·0.32H_2_O	*Pn*	100	24.95	76.39	LS
6a	[Fe^III^(4-NEt_2_-sal_2_323)]ClO_4_	*P*1̅	100	27.87	72.14	LS^13^
6b	[Fe^III^(4-NEt_2_-sal_2_323)]PF_6_	*P*1̅	100	27.62	71.25	LS
6b·S	[Fe^III^(4-NEt_2_-sal_2_323)]PF_6_·0.78MeCN·0.1EtOH	*P*1̅	100	27.69	67.81	LS
			100	31.36	81.93	LS
6c	[Fe^III^(4-NEt_2_-sal_2_323)]OTf^f^	*P*1̅	100	28.78	77.34	LS
			293	27.53	75.7	LS
6d	[Fe^III^(4-NEt_2_-sal_2_323)]BF_4_	*P*2_1_/*n*	100	30.17	79.92	LS
6d·S	[Fe^III^(4-NEt_2_-sal_2_323)]BF_4_·EtOH	*P*2_1_2_1_2_1_	100	26.2	68.86	LS
6e	[Fe^III^(4-NEt_2_-sal_2_323)]NO_3_·CH_2_Cl_2_	*P*2_1_2_1_2_1_	100	26.07	68.45	LS
7a	[Fe^III^(3-Me-sal_2_323)]ClO_4_	*P*2_1_2_1_2_1_	100	26.82	65.95	LS
7b	[Fe^III^(3-Me-sal_2_323)]PF_6_·0.68H_2_O	*C*2/*c*	100	24	61.26	LS
7c	[Fe^III^(3-Me-sal_2_323)]BF_4_	*P*2_1_2_1_2_1_	100	27.18	65.94	LS
8	[Fe^III^(3-Allyl-sal_2_323)]NO_3_·MeCN	*P*2_1_/*c*	100	25.72	76.93	LS
9a	[Fe^III^(3-^*t*^Bu-sal_2_323)]PF_6_·EtOH	*P*2_1_/*c*	100(I)	21.94	50.17	LS
			100(II)	24.24	58.49	LS
9b	[Fe^III^(3-^*t*^Bu-sal_2_323)]BF_4_	*P*4_3_22	293	19.58	44.76	LS
10a	[Fe^III^(sal_2_323)]NO_3_	*P*2_1_/*c*	100	28.27	80.76	LS^14^
10b	[Fe^III^(sal_2_323)]BPh_4_	*P*2_1_/*n*	293	28.43	74.16	LS^12^
10c	[Fe^III^(sal_2_323)]Cl	*Pccn*	100	30.1	88.4	LS^11^
10d	[Fe^III^(sal_2_323)]ClO_4_	*P*2_1_/*c*	100	27.29	69.38	LS^15^
10e	[Fe^III^(sal_2_323)]FeCl_4_	*P*2_1_2_1_2_1_	100	26.02	68.49	LS
10f	[Fe^III^(sal_2_323)]BF_4_	*P*2_1_/*c*	100	25.3	63.64	LS
11a	[Fe^III^(5-Br-sal_2_323)]PF_6_	*P*2_1_	293	25.94	65.46	LS
11b	[Fe^III^(5-Br-sal_2_323)]BF_4_·EtOH	*P*1̅	100	22.38	59.2	LS
11c	[Fe^III^(5-Br-sal_2_323)]NO_3_·^*i*^PrOH	*P*2_1_/*n*	100 (I)	25.95	71.41	LS
			100(II)	25.92	68.38	LS
12	[Fe^III^(3,5-diBr-sal_2_323)]NO_3_·^*i*^PrOH	*P*2_1_/*n*	100	24.74	63.01	LS
13a	[Fe^III^(3,5-diCl-sal_2_323)]BF_4_·^*i*^PrOH	*P*2/*n*	293(I)	23.63	60.99	LS
			293(II)	29.9	78.11	LS
13b	[Fe^III^(3,5-diCl-sal_2_323)]PF_6_	*P*2_1_/*n*	100	24.54	60.26	LS
14	[Fe^III^(3,5-diI-sal_2_323)]PF_6_	*P*2_1_/*c*	100	25.7	64.96	LS
15a	[Fe^III^(3-NO_2_-sal_2_323)]PF_6_·MeCN	*P*2_1_/*n*	293	27.96	65.59	LS
15b	[Fe^III^(3-NO_2_-sal_2_323)]NO_3_	*Cc*	100	29.94	72.17	LS
16a	[Fe^III^(5-NO_2_-sal_2_323)]PF_6_·EtOH	*P*1̅	293	24.05	63.44	LS
16b	[Fe^III^(5-NO_2_-sal_2_323)]BF_4_·EtOH	*P*1̅	293	22.36	58.7	LS
16c	[Fe^III^(5-NO_2_-sal_2_323)]ClO_4_·EtOH	*P*1̅	100	21.02	56.65	LS
17	[Fe^III^(3,5-diNO_2_-sal_2_323)]ClO_4_·EtOH	*P*2_1_/*n*	100	27.75	76.98	LS

a Seven of the complexes have been previously reported and are indicated with their respective literature reference.

b Refers to the temperature of the diffraction experiment.

c Structures with more than one independent Fe^III^ site in the asymmetric unit are indicated as (I) and (II).

d Distortion parameters Σ and Θ are described in the main text.

e Those structures previously reported in the literature are indicated with the appropriate reference.

f Where OTf is CF_3_SO_3_.

### Single-Crystal X-ray Diffraction

Single-crystal X-ray diffraction (SCXRD) is a powerful diagnostic technique for the assignment of the spin state in transition metal complexes.^5,19^ Changes in the spin state are intimately linked with the bond lengths and distortions around metal centers, with the LS state having shorter bond lengths and less distortion and the HS state having longer bond lengths and more distortion. These local distortions at the metal centers can have dramatic effects on intermolecular interactions and packing effects and sometimes result in changes in symmetry.^6^ The structures of **1–17** were determined using SCXRD, with the experiment performed at either 100 K or room temperature (293 K). In all cases, the asymmetric unit contains the [Fe^III^(R-sal_2_323)]^+^ cation, a charge balancing anion, and, in some cases, a lattice solvent. The Fe^III^ center adopts a *pseudo*-octahedral geometry with an N_4_O_2_^2–^ coordination sphere. The configuration of the ligand results in the orientation of the phenolic oxygen atoms trans to one another and the amine/imine atoms cis to one another. The bond lengths around the Fe^III^ center are relatively short, with the average Fe–O_phen_, Fe–N_imine_, and Fe–N_amine_ for all complexes of ∼1.87–1.89, ∼1.94–1.96, and ∼2.01–2.04 Å, respectively (see Table S2.2, Supporting Information).

### Structural Characterization of Complex Families **2–17**

Most of the complexes crystallize in centrosymmetric space groups (Tables 1 and S2.1, Supporting Information), but there are several examples where the complexes crystallize in non-centrosymmetric enantiopure space groups. When the complexes crystallize in the same space group, they are often isostructural. The most common space group found was *P*2_1_/*c* (*P*2_1_/*n*) with most of the complexes crystallizing with *Z* = 4, *Z*′ = 1, except **9a** and **11c** which crystallize with two independent molecules in the asymmetric unit (*Z*′ = 2). These complexes form 1D intermolecular hydrogen bonding chains between cations and anions (and the lattice solvent, where present), Table S2.3. Complexes that crystallize in the *P*1̅ space group (**6a–6c**, **11b**, **16a–16c**) have *Z* = 2, *Z*′ = 1 (except **6b·S** with *Z*′ = 2) and also feature 1D hydrogen bonding chains. Both **3a** and **3b** crystallize in *P*2/*c* with two half cations in the asymmetric unit (bisected by a twofold rotational axis) and **2a** and **10c** in *Pccn* with *Z*′ = 0.5. Those that crystallize in non-centrosymmetric space groups include **6d·S–6e**, **7a**, **7c**, and **10e** in *P*2_1_2_1_2_1_; **2e** and **11a** in *P*2_1_; and **9b** in *P*4_3_22. These complexes all facilitate strong intermolecular hydrogen bonding and other close contacts, likely helping stabilize the spin doublet ground state. The bond lengths of these complexes are all consistent with those expected for spin doublet Fe^III^.^11−16^

### Structural Characterization of Complex Family **1**

The structures of those complexes with the 4-methoxy substitution (**1a–1h**) were investigated in further detail. Complexes **1a·S**, **1b–1h** reliably crystallize in the centrosymmetric monoclinic *P*2_1_/*c* (*P*2_1_/*n*) space group and consist of similar asymmetric units containing a single cation, single anion, and, in some cases, lattice solvent. Bond lengths around the Fe^III^ center are consistent with those of the structures of **2–17**. Hydrogen bond chain formation is observed in all cases through N–H···X interactions with the anion or lattice solvent (where X is O/F/I depending on the anion/solvent), Table S2.3.

**Figure 2:**
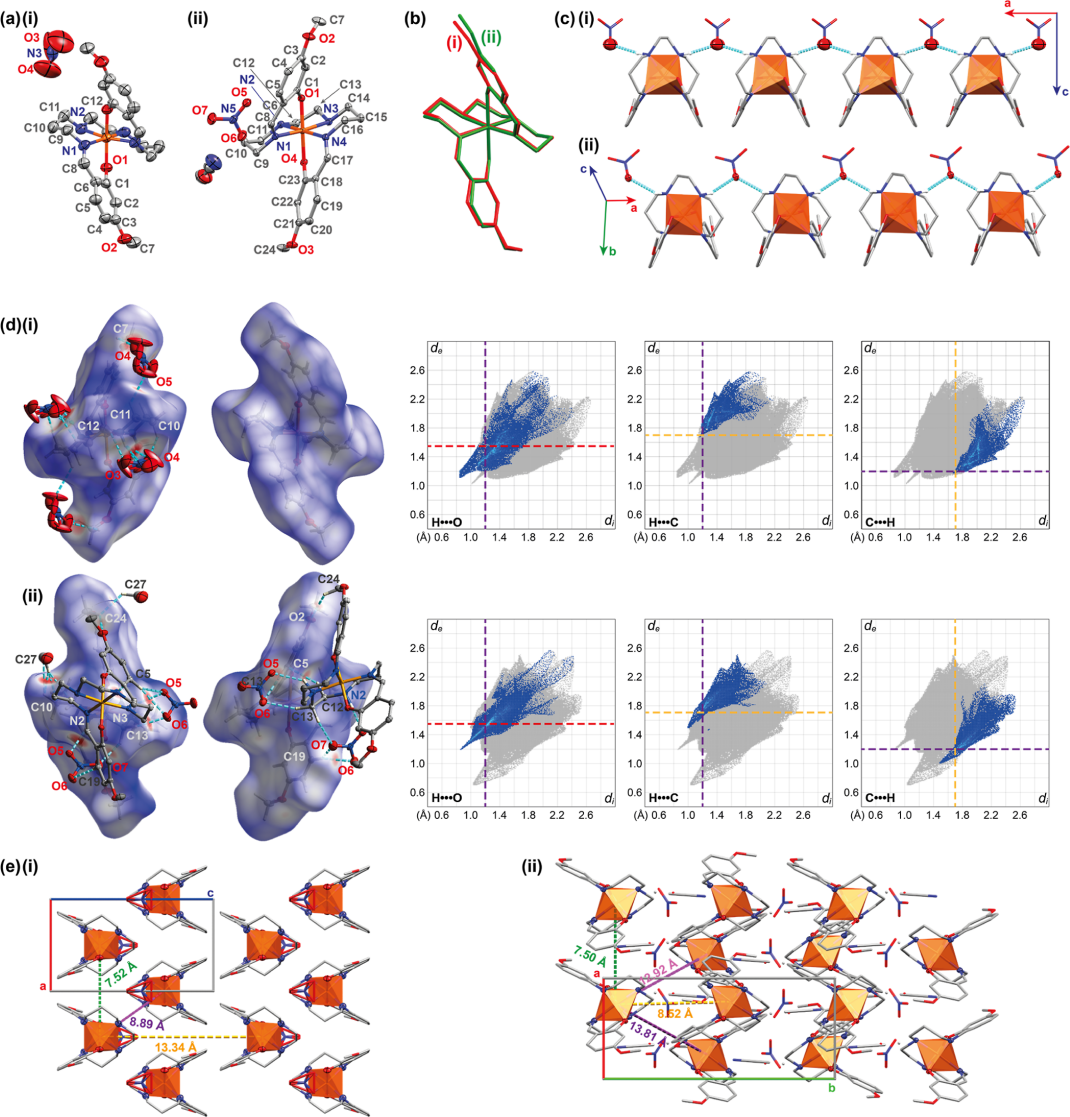
(a) Molecular structure of **1a** (i) and **1a·S** (ii) at 100 K. Hydrogen atoms have been omitted for clarity. Ellipsoids are drawn at 50% probability. The asymmetric unit of **1a** consists of a half molecule as the molecule is bisected by a twofold rotational axis. Atomic labeling is shown for the asymmetric unit. (b) Molecular overlay of the [Fe^III^(4-OMe-sal_2_323)]^+^ cation for **1a** and **1a·S** at 100 K. (c) Intermolecular hydrogen bonding network formed between the cation and anion for (i) **1a** and (ii) **1a·S**. (d) Hirshfeld surface analysis for **1a** and **1a·S** at 100 K with two views of the cation for both. The surface is mapped with *d*_norm_ values of −0.4773–1.2245 a.u. **1a** and −0.4228–1.3330 a.u. **1a·S**. Contacts which are shorter than the vdW radii of the contact atoms are shown in red on the surface and contacts longer than the vdW radii in blue. Contacts which are shorter than the vdW radii are indicated with a dashed blue line and labeled according to the contact atoms inside (white) and outside the surface (colored). Two-dimensional fingerprint plots delineated into H···O, H···C, and C···H contacts, with *d*_i_ on the *x*-axis (distance from the surface to the closest internal atom) and *d*_e_ on the *y*-axis (distance from the surface to the closest external atom). The dashed lines on the plots refer to the vdW radii of the selected atoms:^22^ hydrogen 1.2 Å, purple; oxygen 1.55 Å, red; and carbon 1.7 Å, orange. (e) Packing diagram of (i) **1a** and (ii) **1a·S** with internuclear Fe–Fe distances indicated. The Fe^III^ centers are indicated as polyhedra.

The exception to the series is for the [Fe^III^(4-OMe-sal_2_323)]NO_3_ complex, **1a**. We identified two solvatomorphs in the as-recovered batch, one which crystallized in the centrosymmetric monoclinic *P*2_1_/*n* space group with *Z*′ = 1 (**1a·S**) and one which crystallized in the non-centrosymmetric orthorhombic *P*2_1_2_1_2 space group as an inversion twin with *Z*′ = 0.5 (**1a**), Figure 2. **1a·S** has a lattice solvent (acetonitrile/methanol) present, whereas **1a** does not. Although **1a·S** has short bond lengths, similar to those of **1b–1h**, in **1a**, we observe relatively longer bond lengths, Table 2, along with an elongation of bond lengths on warming from 100 to 293 K, suggesting a thermal change in the spin state. The changes in bond length between 100 and 293 K are of the order of ΔFe–O_avg_ = 0.015 Å and ΔFe–N_avg_ = 0.059 Å. These values are slightly smaller than those observed in other Fe^III^ spin crossover complexes, most likely due to the incomplete nature of the transition between 100 and 293 K.^3^ Coupled to the changes in bond lengths, we also observe an increase in the polyhedron volume (*V*_P_) upon changing temperature, Table 2. The volume of **1a** is greater than that of **1a·S** and increases 5.5% between 100 and 293 K. Typical *V*_*P*_ changes upon spin crossover are of the order of 25% for Fe^II^ and 17% for Fe^III^. The increase in volume of **1a** is not significant enough to result in a complete change in the spin state.

**Table 2:** Summary of Bond Lengths, Polyhedron Volume, and Distortion Parameters for **1a**

	**1a**		**1a·S**	
*T* (K)	100	293	100	293
Bond Length (Å)				
Fe–O	1.908(2)	1.923(3)	1.8785(11)	1.8768(10)
			1.8858(11)	1.8841(10)
Fe–N_imine_	1.982(2)	2.048(4)	1.9514(12)	1.9487(12)
			1.9545(13)	1.9528(13)
Fe–N_amine_	2.051(3)	2.102(4)	2.0154(12)	2.0166(12)
			2.0171(13)	2.0175(13)
*V*_P_ (Å^3^)	10.27	10.83	9.85	9.84
Δ*V*_P_	+5.5%		–0.1%	
Distortion Parameters (°)				
Σ	34.26	52.06	24.13	25.22
Θ	127.29	200.78	58.94	63.53
α	49.39	50.93	44.29	45.89
τ	30.80	30.02	26.04	25.17
			26.84	26.10

Intermolecular interactions for **1a** and **1a·S** were investigated using Hirshfeld surface analysis^20,21^ and are summarized in Figure 2d. A hydrogen bonding chain is present in **1a** between the amines of the backbone and the nitrate anion, N2–H2···O3. This interaction is shorter than the sum of the van der Waals (vdW) radii of the corresponding atoms. The length of the hydrogen bond increases upon increasing temperature from 100 to 293 K, Table 3. Hirshfeld surface analysis of **1a** reveals that the surface is made up primarily of H···H contacts (54.5%), followed by O···H/H···O contacts (24.0%). There are C–H···O interactions which are shorter than the sum of the vdW radii; they increase upon heating to 293 K, with the C10–H10A···O3 interaction being longer than the sum of the vdW radii at 293 K. At 293 K, the relative contribution from O···H/H···O contacts decreases to 21.9%, Table S2.5. In **1a·S**, a hydrogen bonding network forms between cations and anions, along with a number of direct cation–cation C–H···O/C–H···C interactions which are shorter than the sum of the vdW radii. There is a slight increase in the distance of intermolecular interactions upon heating to 293 K. The relative contribution to the Hirshfeld surface is similar to that of **1a**; however, the addition of N···H/H···N interactions, due to the acetonitrile solvent molecule, makes up 5.6% of the surface. The Hirshfeld analysis for **1a·S** and **1a** at 293 K is presented in Supporting Information, Section S2.4.

**Table 3:** Summary of Intermolecular Interactions for **1a** and **1a·S**

D–H···A	*d*(D–H) (Å)	*d*(H···A) (Å)	*d*(D···A) (Å)	<(DHA) (°)	*d*(D–H) (Å)	*d*(H···A) (Å)	*d*(D···A) (Å)	<(DHA) (°)
	100 K				293 K			
**1a** [Fe^III^(4-OMe-sal_2_323)]NO_3_								
N2–H2···O3^a^	1.00	2.07	2.910(13)	140.1	0.98	2.07	2.974(4)	153.3
C10^b^–H10A^b^···O3^c^	0.99	2.54	3.221(9)	125.9	0.97	2.79	3.498(7)	130.6
C12^b^–H12B^b^···O4^c^	0.99	2.47	3.19(1)	129.4	0.97	2.67	3.29(1)	121.9
**1a·S** [Fe^III^(4-OMe-sal_2_323)]NO_3_·0.75MeCN·0.25MeOH								
N2–H2···O5^d^	1.00	2.06	2.9551(17)	147.3	0.98	2.11	2.9785(18)	147.1
N3–H3···O5^e^	1.00	2.21	3.0376(18)	139.2	0.98	2.24	3.054(2)	139.6
C13–H13A···O6^e^	0.99	2.35	3.252(2)	151.8	0.97	2.40	3.285(3)	151.7
C24–H24A···O2^e^	0.98	2.55	3.299(2)	133.4	0.96	2.60	3.390(3)	139.8
C12–H12B···C5^e^	0.99	2.58	3.453(2)	147.3	0.97	2.64	3.484(2)	145.7
C19–H19···O6^f^	0.95	2.51	3.406(2)	156.8	0.93	2.58	3.460(3)	158.4
C5–H5···O6	0.95	2.50	3.359(2)	151.3	0.93	2.56	3.411(2)	152.0

a Symmetry transformations used to generate equivalent atoms: −*x* + 1/2, *y* + 1/2, −*z* + 1.

b Symmetry transformations used to generate equivalent atoms: −*x*,–*y* + 1, *z*.

c Symmetry transformations used to generate equivalent atoms: *x* – 1/2, −*y* + 1/2, −*z* + 1.

d Symmetry transformations used to generate equivalent atoms: −*x* + 3/2, *y* – 1/2, −*z* + 3/2.

e Symmetry transformations used to generate equivalent atoms: −*x* + 1/2, *y* – 1/2, −*z* + 3/2.

f Symmetry transformations used to generate equivalent atoms: *x* – 1, *y*, *z*.

Comparison of the intermolecular interactions for the spin labile **1a** with those of the remainder of complexes **1–17** reveals little difference between them: the vast majority feature formation of the hydrogen bonding chain between the cations, anions, and lattice solvent (when present). We do not identify any specific structural or intermolecular interactions which lead to **1a** supporting the HS state over the other complexes. However, the fact that in the absence of the lattice solvent complex **1a** displays a thermal spin crossover indicates that stabilization of both spin doublet and sextet forms of Fe^III^ is possible in this N_4_O_2_^2–^ ligand sphere. Quenching of the spin crossover upon inclusion of solvent molecules suggests that subtle lattice pressure differences may play a greater role in choice of the spin state than intermolecular interactions.

### Distortion Parameters

We also used some structural distortion parameters to quantify the degree of molecular distortion around the Fe^III^ center. The octahedral distortion parameters, Σ and Θ, are commonly used to diagnose the spin state.^23−26^
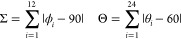


**Figure 3:**
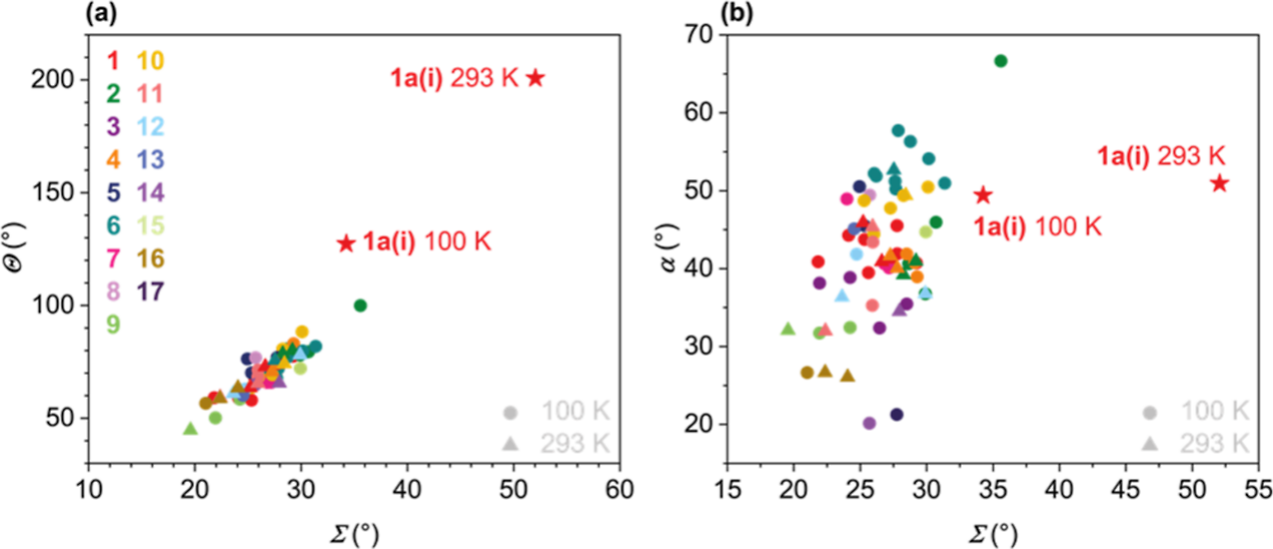
Plot of distortion parameters Σ and Θ (a) and Σ and α (b) for **1–17** at either 100 K (circles) or 293 K (triangles). The points are colored based on their respective ligand as indicated. The points for **1a** are denoted with red stars as the chief outlier which undergoes thermal spin crossover.

The Σ parameter is the sum of the deviation of the 12 unique cis ligand–metal–ligand angles (ϕ_*i*_) from 90°. The Θ parameter is the sum of the deviation of the 24 unique torsional angles between ligand atoms on opposite triangular faces of the octahedron. In addition, we define two other distortion parameters, α and τ. The α parameter can be defined as the dihedral angle between the least squares planes of the two phenolate rings, as utilized by Halcrow et al. for [Fe^III^(R-sal_2_222)]^+^ complexes.^27^ The τ parameter can be defined as the Fe–O–C–C torsional angle, also utilized by Halcrow et al.^27^ The ideal value for a perfect octahedron for all of the distortion parameters would be 0°. The distortion parameters for **1–17** are summarized in Table S2.2. We observe a range of values for Σ between 19.58 and 35.59° and Θ between 44.76 and 99.98°, for those complexes confirmed as LS (*S =* 1/2). These values are greater than the range expected for LS [Fe^III^(R-sal_2_222)]^+^ complexes.^27^ In **1a**, the values of Σ and Θ are outside of the range described previously, with values of Σ = 34.26° and Θ *=* 127.29° at 100 K and Σ = 52.06° and Θ *=* 200.78° at 293 K. The 293 K value is larger than the 100 K value, suggesting a thermal change in distortion, which would be coupled to spin crossover. When comparing this value to that of other known HS (*S =* 5/2) complexes with related Schiff base ligands with different chelate sizes, namely, [Fe^III^(R-sal_2_222)]^+^, [Fe^III^(R-sal_2_232)]^+^, and [Fe^III^(R-sal_2_333)]^+^, we find that the values obtained for **1a** are more closely related to those of the [Fe^III^(5-F-sal_2_333)]^+^ complex (Σ = 57.97°, Θ *=* 230.34°) than those of the “232” and “222” analogues (Table S2.4, Supporting Information). The tighter chelation of the “222” and “232” tetraamine backbones, leading to a *cis* configuration of phenolate donors, likely requires further distortion to facilitate the HS state, compared to that of the longer “323” and “333” backbones, which have a *trans* configuration of the phenolate donors. The relationship between Σ and Θ is close to linear (*R*^2^ = 0.90), Figure 3a.

We observe a range of values of α between 20.18 and 66.64° and τ between 13.84 and 36.52°. The link between α and τ and the expected spin state of the complex is less clear. In general, the α and τ values are closely grouped according to the ligand type, Figure 3b. Complex **1a** has a relatively large value of α and τ at 49.39 and 30.80° at 100 K and 50.93 and 30.02° at 293 K, respectively. There is a minimal change in these values across a temperature change. There is another outlier on the plot in Figure 3, **2a** (green circle), which has large values for Σ, Θ, and α of 35.59, 99.98, and 66.64°, respectively. This sample does not undergo thermal spin crossover, with 293 K data revealing Σ, Θ, and α parameters of 29.19, 80.03, and 40.95°, respectively, a decrease from the lower temperature values.

### Magnetic Characterization

**Figure 4:**
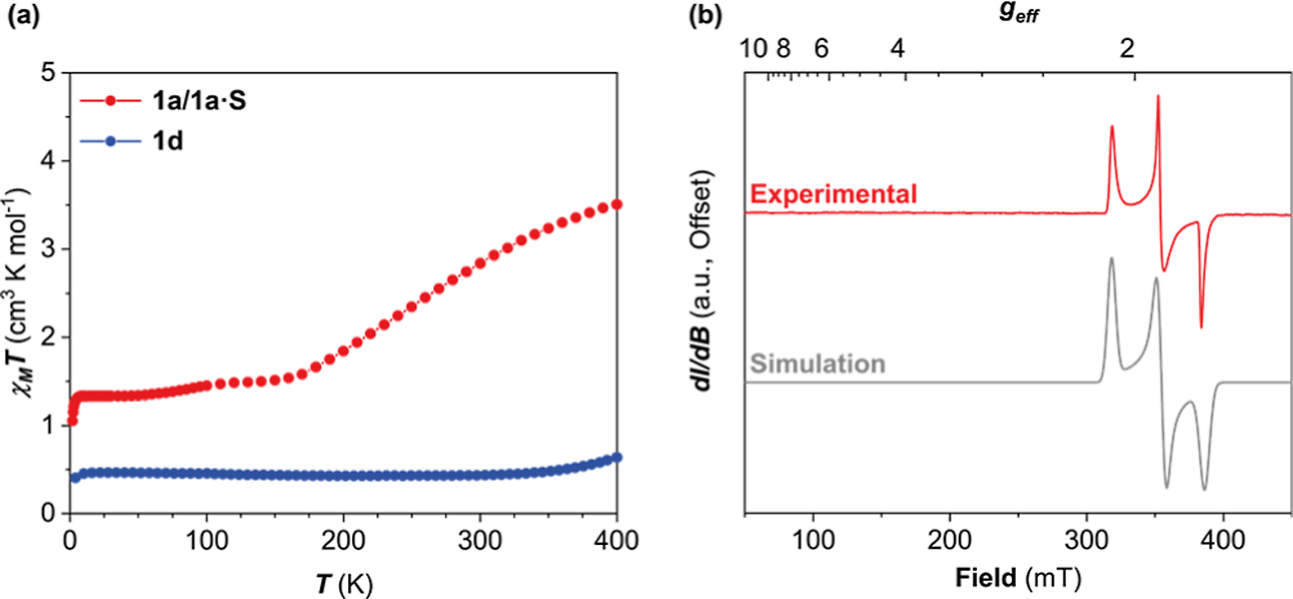
(a) Magnetic susceptibility (χ_M_*T*) recorded between 400 and 2 K for **1a**/**1a·S** and **1d**. (b) EPR spectrum for **1a**/**1a·S** recorded in a frozen solution of ethanol/methanol (1:9) at 77 K between 50 and 450 mT.

The spin state of these Fe^III^ complexes can readily be assigned using crystallography, that is, by analysis of bond lengths around the Fe^III^ center and consideration of distortion parameters. We utilize this as a routine indicator of the spin state; however, it only gives information at the specific measurement temperature and does not easily reveal the nature of the spin state transition (where present). In the case of **1a**, where we have evidence of spin crossover, we investigated the magnetic susceptibility of the sample between 2 and 400 K. A plot of χ_M_*T* versus *T* for the as-recovered polycrystalline mixture of **1a** and **1a·S** reveals a gradual, incomplete transition, Figure 4. At 400 K, the **1a**/**1a·S** sample reaches a χ_M_*T* value of 3.5 cm^3^ K mol^–1^, while at low temperature, the value plateaus at a value of 1.33 cm^3^ K mol^–1^, which is higher than that of the fully LS **1d** (vide infra). This suggests that the SCO-active component (**1a**) in the mixture does not reach a fully LS state on cooling, which is in line with the longer bond lengths for the **1a** solvatomorph at 100 K, Table 2. A Boltzmann fit of the χ_M_*T* curve for **1a**/**1a·S**, Figure S3.1, reveals a *T*_1/2_ value of 265 K for the gradual transition, which would saturate at higher temperatures at a value of χ_M_*T* = 3.65 cm^3^ K mol^–1^.

The incomplete nature of the thermal spin crossover is likely due to the fact that only solvatomorph **1a** shows spin state switching, while crystallography indicates that solvatomorph **1a·S** is LS at temperatures up to 293 K (Table 2). Powder diffraction data of the polycrystalline sample of solvatomorphs used for the susceptibility experiment were collected several months after the magnetic data were measured. Comparison with the simulated powder data of components **1a** and solvatomorph **1a·S** suggests the presence of only the spin crossover component **1a**, Figure S5.1, by the time of the powder diffraction measurement, which is not surprising given the likelihood that the solvated component converts to the unsolvated species on standing or during sample preparation for powder diffraction measurements. However, we have interpreted the magnetic data assuming the presence of both the spin crossover solvatomorph **1a** and the LS solvated solvatomorph **1a·S** in the freshly recovered product. The presence of small amounts of LS **1a·S** will damp the χ_M_*T* value at elevated temperatures. Bond length data also indicate that the dominant spin labile component **1a** is not fully LS at 100 K, which may account for the higher than expected χ_M_*T* value of 1.33 cm^3^ K mol^–1^. The challenges associated with including an accurate diamagnetic correction for a mixture of solvatomorphs are also a factor here.

By way of comparison, the thermal response of χ_M_*T* for LS complex **1d** is also shown in Figure 4, where a χ_M_*T* value of 0.46 cm^3^ K mol^–1^ is observed over most of the measured range. The upturn in χ_M_*T* above 350 K for complex **1d** indicates onset of thermal spin crossover at higher temperature, underscoring the delicate balance of the two spin states which can be achieved within this complex family.

Electron paramagnetic resonance (EPR) spectroscopy was also used to characterize the electronic structure of the polycrystalline **1a**/**1a·S** sample. EPR measurements collected on a frozen solution (EtOH/MeOH) showed a characteristic rhombic LS Fe^III^ EPR signal, Figure 4 b, and fitting reveals *g*_*x*_ = 1.75, *g*_*y*_ = 1.89, and *g*_*z*_ = 2.10 for an *S* = 1/2 assignment. Although the very minor feature close to *g* = 4, Figure 4, possibly indicates a trace HS component, this is tenuous, and it is more likely that the spin state of the free [Fe^III^(4-OMe-sal_2_323)]^+^ cation in frozen glass is fully LS, which would be in agreement with the susceptibility data in solution at room temperature as determined using Evan’s method.^28^ The NMR experiment was performed at 25 °C in DMSO-*d*_6_. A χ_M_*T* value of 0.69 cm^3^ K mol^–1^ was obtained for **1a** and a value of 0.76 cm^3^ K mol^–1^ for **1d**. As both the nitrate and perchlorate salts of [Fe^III^(4-OMe-sal_2_323)]^+^ are LS in solution, we suggest that the thermal change in the spin state observed in the bond length data for **1a** and the susceptibility data of the mixture of solid **1a**/**1a·S** is a result of packing in the crystalline form of the unsolvated nitrate complex [Fe^III^(4-OMe-sal_2_323)]NO_3_, **1a**.

### Quantum Chemical Calculations

**Figure 5:**
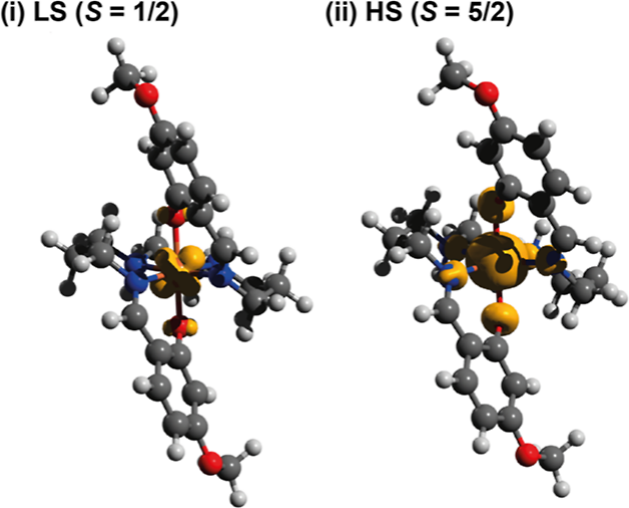
Isocontour plots (0.008 a.u.) of unpaired electron spin density plots of the [Fe^III^(4-OMe-sal_2_323)]^+^ cation for the spin doublet (i) and spin sextet (ii) states.

A theoretical study was carried out using ORCA 4.2.1.^29,30^ For the purposes of the calculations, the cations of the ligands hosting the unsubstituted salicylaldehyde, [Fe^III^(sal_2_323)]^+^, and that with the 4-methoxy-substituted salicylaldehyde, [Fe^III^(4-OMe-sal_2_323)]^+^, were considered. As such, we have not considered the effects of the anion or packing in a crystalline lattice but only the electronic effects of substitution and differences between the doublet and sextet states. The experimentally determined SCXRD structures were used as a starting point for geometry optimization. In both cases, the structures were optimized for the spin doublet (*S* = 1/2) and spin sextet (*S =* 5/2) states, Figure 5. To ensure that a true energy minimum had been reached and not a saddle point, an analytical frequency calculation was performed on the optimized geometries. Comparison of the optimized and experimental geometries reveals similarities in the bond lengths, angles, and distortion parameters, Table 4. We expect slight deviations from experimental structures since the crystal packing effect and intermolecular interactions are not considered. The 293 K structure of **1a** has shorter bond lengths and smaller distortion parameters than those of the optimized sextet state, which is likely because the Fe^III^ center has not reached a complete HS state at this temperature, which we confirmed by magnetic susceptibility measurements, Figure 4a.

**Table 4:** Bond Lengths and Distortion Parameters of the Optimized Geometries of [Fe^III^(sal_2_323)]^+^ and [Fe^III^(4-OMe-sal_2_323)]^+^ and the Experimental Structures of **1a** and **1a**·**S** for Comparison

	1a		1a·S		[Fe^III^(4-OMe-sal_2_323)]^+^^a^		[Fe^III^(sal_2_323)]^+^^a^	
*T* (K)	100	293	100	293	*S* = 1/2	*S* = 5/2	*S* = 1/2	*S* = 5/2
Bond Lengths (Å)								
Fe–O	1.908(2)	1.923(3)	1.8785(11)	1.8768(10)	1.887	1.958	1.881	1.944
			1.8858(11)	1.8841(10)	1.887	1.958	1.881	1.944
Fe–N_imine_	1.982(2)	2.048(4)	1.9514(12)	1.9487(12)	1.922	2.100	1.925	2.126
			1.9545(13)	1.9528(13)	1.922	2.101	1.925	2.126
Fe–N_amine_	2.051(3)	2.102(4)	2.0154(12)	2.0166(12)	2.045	2.241	2.043	2.240
			2.0171(13)	2.0175(13)	2.045	2.241	2.043	2.239
Distortion Parameters (°)								
Σ	34.26	52.06	24.13	25.22	30.24	78.92	30.12	82.71
Θ	127.29	200.78	58.94	63.53	67.22	308.47	67.90	338.89
α	49.39	50.93	44.29	45.89	40.15	66.21	39.24	68.39
τ	30.80	30.02	26.04	25.17	25.36	27.32	25.14	26.95
			26.84	26.10	25.35	27.45	25.20	26.97

a Obtained from the geometry-optimized structures of the [Fe^III^(4-OMe-sal_2_323)]^+^ and [Fe^III^(sal_2_323)]^+^. See experimental details for more information.

Kepp benchmarked a number of functionals to known spin crossover Fe^II^ and Fe^III^ complexes and found that the B3LYP* (15% HF exchange) was the most accurate in determining the free energy of spin crossover, Δ*G*_SCO_.^31^ We utilize the methodology described in that work to determine Gibb’s free energy, Δ*G*_SCO_. Thermodynamic corrections, Δ*G*_therm_, are added to the electronic energy difference, Δ*E*. The thermodynamic corrections are negative because of the increased vibrational entropy and zero-point vibrational energy in the HS state, Table S4.2. A Δ*G*_SCO_ value of +18.4 kJ mol^–1^ for the [Fe^III^(4-OMe-sal_2_323)]^+^ cation and +18.8 kJ mol^–1^ for the [Fe^III^(sal_2_323)]^+^ cation are obtained, showing a clear stabilization of the doublet (*S* = 1/2) ground state. There is little difference between the unsubstituted complex and the methoxy-substituted analogue; as such, the thermal spin crossover in **1a** is more likely because of the effects of packing and intermolecular interactions due to the crystalline environment and less likely a result of electronic influence of ligand substitution as there is little change in Δ*G*_SCO_ between [Fe^III^(4-OMe-sal_2_323)]^+^ and [Fe^III^(sal_2_323)]^+^.

## Conclusions

We have synthesized and characterized a variety of [Fe^III^(R-sal_2_323)]X complexes, **1–17**. All except one exist in the LS (*S* = 1/2) spin state, as measured by metal–ligand bond lengths and distortion parameters. Magnetic susceptibility measurement of the mixture of **1a** and **1a·S** shows gradual and incomplete thermal spin crossover, which is coupled to lengthening of the bond lengths in **1a**. We do not identify specific structural effects that specifically promote the HS state in **1a** compared to the larger library of LS complexes reported, but the thermal spin crossover in the solid state for this one example demonstrates the fine energy balance between spin doublet and sextet states for Fe^III^ in the R-sal_2_323 coordination sphere. The mixture of **1a** and **1a·S** retains LS (*S* = 1/2) characteristics in solution, with a χ_M_*T* value of 0.69 cm^3^ K mol^–1^ obtained using the Evans method ^1^H NMR. Investigation by density functional theory (DFT) shows little energy difference in the unsubstituted salicylaldehyde complex, [Fe^III^(sal_2_323)]^+^, and the 4-methoxy-substituted salicylaldehyde analogue, [Fe^III^(4-OMe-sal_2_323)]^+^, but a net favoring of the LS (*S* = 1/2) state for both cations.

## Experimental Methods

### Synthesis

The synthetic procedure for **1–17** is described in detail in Supporting Information, S1.

### Physical Measurements

Elemental analysis (C, H, N) was carried out using an Exeter Analytical CE-440 Elemental Analyzer. Magnetic susceptibility measurements were performed using a Quantum Design MPMS-XL SQUID magnetometer operating between 2 and 400 K. Polycrystalline samples were packed in a gelatin capsule. Diamagnetic corrections were applied to correct for the inherent diamagnetism of the gelatin capsule and the samples using Pascal’s constants.^32^

### Single-Crystal X-ray Diffraction

SCXRD was performed using suitable single crystals with either a Bruker SMART APEX CCD area detector diffractometer or a Rigaku Oxford Diffraction SuperNova diffractometer. Data sets were collected using either monochromatic Cu-Kα or Mo-Kα radiation. Measurements were performed at either 100 K or room temperature (293 K).

For the data collected on the Bruker diffractometer, Bruker SMART software was used for the data collection and integration,^33^ Bruker SAINT software was used for the data reduction,^34^ and empirical absorption corrections were performed using the SADABS program.^35^ The structures were solved using direct methods in SHELXS-97 and refined using full-matrix least-squares minimization on F^2^ using SHELXL-97.^36^

For the data collected on the Rigaku Oxford Diffraction diffractometer, CrysAlis^PRO^ software was used for the data collection, integration, reduction, and finalization.^37^ A numerical absorption correction based on the shape of the crystal and an empirical correction were performed using CrysAlis^PRO^. The structures were solved using direct methods in SHELXS and refined using full-matrix least-squares minimization on F^2^ using SHELXL.^38^

Hydrogen atoms were geometrically constrained and refined riding on the parent atoms, except for hydrogens attached to heteroatoms, which were typically located in the difference Fourier map and allowed to refine freely. Anisotropic displacement parameters were used for all non-hydrogen atoms, except where not possible due to disorder. Further crystallographic details can be found in Supporting Information, S2. CCDC 2166978–2167028 and 2044252, 2044253, and 2044257 contain the supplementary crystallographic data for this paper. The data can be obtained free of charge from The Cambridge Crystallographic Data Centre via www.ccdc.cam.ac.uk/structures.

Hirshfeld surface analysis^20,21^ was used to investigate intermolecular interactions using CrystalExplorer 21 software.^39^

Octahedral distortion parameters were calculated using OctaDist.^40^

### Quantum Chemistry Calculations

Theoretical quantum chemistry calculations were performed using the ORCA 4.2.1 computational chemistry program.^29,30^ Structural data obtained from SCXRD was used as a starting geometry for geometry optimization. The cations of [Fe^III^(sal_2_323)]^+^ and [Fe^III^(4-OMe-sal_2_323)]^+^ were optimized at the DFT level using the BP86 functional^41,42^ and the polarized triple ζ def2-TZVP basis set^43^ together with the atom-pairwise dispersion correction (D3BJ).^44,45^ The resolution of identity approximation was used along with the def2/J auxiliary basis set.^43^ Increased integration grids (Grid5 in ORCA 4.2.1) and tight self-consistent field convergence criteria were used in all calculations. Cartesian coordinates of all the optimized structures can be found in Supporting Information, Table S4.1. Analytical frequencies were calculated at the BP86-def2-TZVP level in order to determine zero-point vibrational energies and thermodynamic corrections Δ*G*_therm_. Single-point energies were subsequently calculated with the B3LYP* functional^46,47^ (15% HF exchange) and the fully polarized def2-TZVPP basis set^48^ and the D3BJ correction.

### Evan’s Method ^1^H NMR

Magnetic susceptibility measurements in solution were obtained using Evan’s method^28^ using an Agilent DD2 500 MHz spectrometer. A standard 5 mm NMR tube was fitted with a 3 mm coaxial insert tube containing pure DMSO-*d*_6_. The standard 5 mm NMR tube was filled with 2.47 mg of **1a** in 400 μL of DMSO-*d*_6_. The NMR tube was carefully sealed to avoid solvent evaporation. The molar magnetic susceptibility, χ_M_, for a long cylindrical shape oriented parallel to the magnetic field was calculated according to equation X, where Δν is the shift in the frequency of the reference solvent peak in Hz, χ_0_ is the molar susceptibility of the solvent, ν_0_ is the operating frequency of the spectrometer in Hz, [*C*] is the concentration of the sample in mol L^–1^, and MW is the molecular mass (of either the solvent or sample)^49^
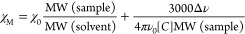


### Electron Paramagnetic Resonance

EPR spectra were measured using a Magnettech MS200 X-band (9.3 GHz) spectrometer between 50 and 450 mT with a modulation amplitude of 0.7 mT and a microwave power of 10 mW. Measurements were performed in a frozen solution of ethanol and methanol (1:9) in LN_2_ (∼77 K) and on a polycrystalline solid. EPR spectra were fitted and simulated using the EasySpin (v5.2.33) software package for MATLAB.^50^

## Supporting Information Available

Synthesis of **1–17**; crystallographic details, bond lengths, distortion parameters, and summary of intermolecular interactions for **1**–**17**; Hirshfeld surface analysis of **1**; further EPR spectra; optimized geometries for the quantum chemistry calculations; powder XRD; and author contributions (PDF)

## Abbreviations

SCO: spin crossover
HS: high spin
LS: low spin
