# Supplementary material for: Crystallographic Detection of the Spin State in FeIII Complexes
Source: Cryst Growth Des. 2022 Oct 17;22(11):6429–39. doi: 10.1021/acs.cgd.2c00468 (PMC9634697; doi:10.1021/acs.cgd.2c00468)
Supplement: Supplementary file 1 — cg2c00468_si_001.pdf [file cg2c00468_si_001.pdf]

Crystallographic Detection of the Spin State in Fe^{III} Complexes

Conor T. Kelly,[†] Michael Griffin,[†] Kane Esien,[‡] Solveig Felton,[‡] Helge Müller-Bunz,[†] Grace G. Morgan.*[†]

Addresses:

[†]School of Chemistry, University College Dublin, Belfield, Dublin 4, D04 N2E5, Ireland.

[‡]School of Mathematics and Physics, Queen's University Belfast, Belfast, BT7 1NN, United Kingdom.

Email: grace.morgan@ucd.ie

Table of Contents

S1 Experimental Methods 4

S1.1 Synthesis of [Fe^{III}(4-OMe-$sal_2$323)]NO_3 (1a) and [Fe^{III}(4-OMe-$sal_2$323)]NO_3·0.75MeCN·0.25MeOH (1a·S) 8

S1.2 Synthesis of [Fe^{III}(4-OMe-$sal_2$323)]PF_6·0.45H_2O (1b) 8

S1.3 Synthesis of [Fe^{III}(4-OMe-$sal_2$323)]OTf·0.27H_2O (1c) 8

S1.4 Synthesis of [Fe^{III}(4-OMe-$sal_2$323)]ClO_4 (1d) 8

S1.5 Synthesis of [Fe^{III}(4-OMe-$sal_2$323)]BF_4 (1e) 8

S1.6 Synthesis of [Fe^{III}(4-OMe-$sal_2$323)]SbF_6·0.31H_2O (1f) 9

S1.7 Synthesis of [Fe^{III}(4-OMe-$sal_2$323)]I_3 (1g) 9

S1.8 Synthesis of [Fe^{III}(4-OMe-$sal_2$323)]Cl·EtOH·0.25H_2O (1h) 9

S1.9 Synthesis of [Fe^{III}(3-OMe-$sal_2$323)]NO_3 (2a) 9

S1.10 Synthesis of [Fe^{III}(3-OMe-$sal_2$323)]BF_4·H_2O (2b) 9

S1.11 Synthesis of [Fe^{III}(3-OMe-$sal_2$323)]PF_6·H_2O (2c) 10

S1.12 Synthesis of [Fe^{III}(5-OMe-$sal_2$323)]NO_3 (3a) 10

S1.13 Synthesis of [Fe^{III}(5-OMe-$sal_2$323)]BF_4 (3b) 10

S1.14 Synthesis of [Fe^{III}(4,6-diOMe-$sal_2$323)]NO_3·MeOH (4a) 10

S1.15 Synthesis of [Fe^{III}(4,6-diOMe-$sal_2$323)]BF_4·0.5MeOH (4b) 10

S1.16 Synthesis of [Fe^{III}(4,6-diOMe-$sal_2$323)]ClO_4·0.5MeOH (4c) 11

S1.17 Synthesis of [Fe^{III}(3-OEt-$sal_2$323)]PF_6·EtOH (5a) 11

S1.18 Synthesis of [Fe^{III}(3-OEt-$sal_2$323)]BF_4·0.32H_2O (5b) 11

S1.19 Synthesis of [Fe^{III}(4-Et_2N-$sal_2$323)]PF_6 (6b) 11

S1.20 Synthesis of [Fe^{III}(4-Et_2N-$sal_2$323)]PF_6·0.78MeOH·0.1EtOH (6b·S) 11

S1.21 Synthesis of [Fe^{III}(4-Et_2N-$sal_2$323)]OTf (6c) 12

S1.22 Synthesis of [Fe^{III}(4-Et_2N-$sal_2$323)]BF_4 (6d) 12

S1.23 Synthesis of [Fe^{III}(4-Et_2N-$sal_2$323)]BF_4·EtOH (6d·S) 12

S1.24 Synthesis of [Fe^{III}(4-Et_2N-$sal_2$323)]NO_3·CH_2Cl_2 (6e) 12

S1.25 Synthesis of [Fe^{III}(3-Me-$sal_2$323)]ClO_4 (7a) 13

S1.26 Synthesis of [Fe^{III}(3-Me-$sal_2$323)]PF_6·0.68H_2O (7b) 13

S1.27 Synthesis of [Fe^{III}(3-Me-$sal_2$323)]BF_4 (7c) 13

S1.28 Synthesis of [Fe^{III}(3-Allyl-$sal_2$323)]NO_3·MeCN (8) 13

S1.29 Synthesis of [Fe^{III}(3-^{t}Bu-$sal_2$323)]PF_6·EtOH (9a) 13

S1.30 Synthesis of [Fe^{III}(3-^{t}Bu-$sal_2$323)]BF_4 (9b) 14

S1.31 Synthesis of [Fe^{III}($sal_2$323)]$FeCl_4$ (10e) 14

S1.32 Synthesis of [Fe^{III}($sal_2$323)]BF_4 (10f) 14

S1.33 Synthesis of [Fe^{III}(5-Br-$sal_2$323)]PF_6 (11a) 14

S1.34 Synthesis of [Fe^{III}(5-Br-$sal_2$323)]BF_4·EtOH (11b) 14

S1.35 Synthesis of [Fe^{III}(5-Br-$sal_2$323)]NO_3·^{i}PrOH (11c) 15

S1.36 Synthesis of [Fe^{III}(3,5-diBr-$sal_2$323)]NO_3·^{i}PrOH (12) 15

S1.37 Synthesis of [Fe^{III}(3,5-diCl-$sal_2$323)]BF_4·^{i}PrOH (13a) 15

S1.38 Synthesis of [Fe^{III}(3,5-diCl-$sal_2$323)]PF_6 (13b) 15

S1.39 Synthesis of [Fe^{III}(3,5-diI-$sal_2$323)]PF_6 (14) 15

S1.40 Synthesis of [Fe^{III}(3-NO_2-$sal_2$323)]PF_6·MeCN (15a) 16

S1.41 Synthesis of [Fe^{III}(3-NO_2-$sal_2$323)]NO_3 (15b) 16

S1.42 Synthesis of [Fe^{III}(5-NO_2-$sal_2$323)]PF_6·EtOH (16a) 16

S1.43 Synthesis of [Fe^{III}(5-NO_2-$sal_2$323)]BF_4·EtOH (16b) 16

S1.44 Synthesis of [Fe^{III}(5-NO_2-$sal_2$323)]ClO_4·EtOH (16c) 16

S1.45 Synthesis of [Fe^{III}(3,5-NO_2-$sal_2$323)]ClO_4·EtOH (17) 17

S2 Single Crystal X-ray Diffraction Details 18

S2.2 Bond Lengths and Distortion Parameters 29

S2.3 Intermolecular Interactions 33

S2.4 Hirshfeld Surface Analysis 38

S3 Magnetic Measurements 39

S4 Quantum Chemistry Calculations 41

S5 Powder X-ray Diffraction 44

S6 Author Contribution 44

S6 References 45

S1 Experimental Methods

Table S1.1. Summary of complex families **1** – **17** compared in this study.

Complex	Salicylaldehyde	Molecular Formula	S.G.	T (K)[a,b]	Σ (°)[c]	Θ (°)[c]	Spin State[d]
1a	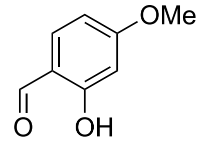<br><br>4-OMe	[Fe^{III}(4-OMe-sal$_2$323)]NO$_3$	$P2_12_12$	100	34.26	127.29	SCO
				293	52.06	200.78	
1a·S		[Fe^{III}(4-OMe-sal$_2$323)]NO$_3$·0.75MeCN·0.25MeOH	$P2_1/n$	100	24.13	58.94	LS
				293	25.22	63.53	
1b		[Fe^{III}(4-OMe-sal$_2$323)]PF$_6$·0.45H$_2$O	$P2_1/c$	100	27.78	71.98	LS
1c		[Fe^{III}(4-OMe-sal$_2$323)]OTf·0.27H$_2$O[e]	$P2_1/c$	100	25.63	68.02	LS
1d		[Fe^{III}(4-OMe-sal$_2$323)]ClO$_4$	$P2_1/c$	100	21.83	59.15	LS
				200	27.34	73.46	
1e		[Fe^{III}(4-OMe-sal$_2$323)]BF$_4$	$P2_1/c$	293	26.62	72.86	LS
1f		[Fe^{III}(4-OMe-sal$_2$323)]SbF$_6$·0.31H$_2$O	$P2_1/c$	100	27.79	71.75	LS
1g		[Fe^{III}(4-OMe-sal$_2$323)]I$_3$	$P2_1/c$	100	25.33	58.11	LS
1h		[Fe^{III}(4-OMe-sal$_2$323)]Cl·EtOH·0.25H$_2$O	$P2_1/c$	100	29.23	77.52	LS
2a	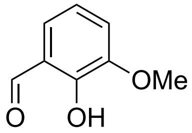<br><br>3-OMe	[Fe^{III}(3-OMe-sal$_2$323)]NO$_3$	Pccn	100	35.59	99.98	LS
				293	29.20	80.03	LS
2b		[Fe^{III}(3-OMe-sal$_2$323)]BF$_4$·H$_2$O	$P2_1/c$	100	28.65	80.74	LS
2c		[Fe^{III}(3-OMe-sal$_2$323)]PF$_6$·H$_2$O	$P2_1/c$	293	28.27	78.45	LS
2d		[Fe^{III}(3-OMe-sal$_2$323)]FeCl$_4$	$P2_1/c$	150	30.71	79.62	LS[1]
2e		[Fe^{III}(3-OMe-sal$_2$323)]ClO$_4$	$P2_1$	173	29.9	78.11	LS[2]
3a	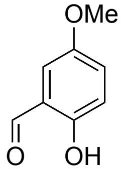<br><br>5-OMe	[Fe^{III}(5-OMe-sal$_2$323)]NO$_3$	$P2/c$	100(I)	21.94	50.17	LS
				100(II)	24.24	58.49	
				293(I)	23.86	61.35	
				293(II)	29.92	73.33	
3b		[Fe^{III}(5-OMe-sal$_2$323)]BF$_4$	$P2/c$	100(I)	28.51	79.56	LS

				100(II)	26.47	70.26	LS
4a	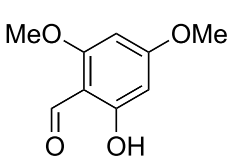	[Fe^{III}(4,6-diOMe-sal$_2$323)]NO$_3$·MeOH	P2$_1$/c	100	29.29	83.14	LS
				293	27.79	73.82	LS
4b		[Fe^{III}(4,6-diOMe-sal$_2$323)]BF$_4$·0.5MeOH	P2$_1$/n	100	29.11	79.30	LS
				293	27.26	70.69	LS
4c	4,6-diOMe	[Fe^{III}(4,6-diOMe-sal$_2$323)]ClO$_4$·0.5MeOH	P2$_1$/n	100	28.51	77.47	LS
5a	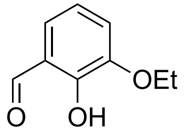	[Fe^{III}(3-OEt-sal$_2$323)]PF$_6$·EtOH	*P*2$_1$/*n*	100	25.32	70.2	LS
5b	3-OEt	[Fe^{III}(3-OEt-sal$_2$323)]BF$_4$·0.32H$_2$O	Pn	100	24.95	76.39	LS
6a		[Fe^{III}(4-NEt$_2$-sal$_2$323)]ClO$_4$	*P*-1	100	27.87	72.14	LS[3]
6b		[Fe^{III}(4-NEt$_2$-sal$_2$323)]PF$_6$	*P*-1	100	27.62	71.25	LS
6b·S	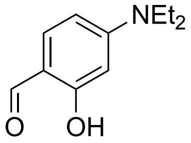	[Fe^{III}(4-NEt$_2$-sal$_2$323)]PF$_6$·0.78MeCN·0.1EtOH	*P*-1	100 (I)	27.69	67.81	LS
				100 (II)	31.36	81.93	LS
6c		[Fe^{III}(4-NEt$_2$-sal$_2$323)]OTf[e]	*P*-1	100	28.78	77.34	LS
				293	27.53	75.7	LS
6d	4-NEt$_2$	[Fe^{III}(4-NEt$_2$-sal$_2$323)]BF$_4$	*P*2$_1$/*n*	100	30.17	79.92	LS
6d·S		[Fe^{III}(4-NEt$_2$-sal$_2$323)]BF$_4$·EtOH	*P*2$_1$2$_1$2$_1$	100	26.2	68.86	LS
6e		[Fe^{III}(4-NEt$_2$-sal$_2$323)]NO$_3$·CH$_2$Cl$_2$	*P*2$_1$2$_1$2$_1$	100	26.07	68.45	LS
7a	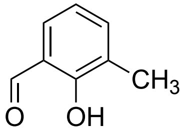	[Fe^{III}(3-Me-sal$_2$323)]ClO$_4$	*P*2$_1$2$_1$2$_1$	100	26.82	65.95	LS
7b		[Fe^{III}(3-Me-sal$_2$323)]PF$_6$·0.68H$_2$O	*C*2/*c*	100	24	61.26	LS
7c	3-Methyl	[Fe^{III}(3-Me-sal$_2$323)]BF$_4$	*P*2$_1$2$_1$2$_1$	100	27.18	65.94	LS
8	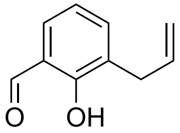 3-Allyl	[Fe^{III}(3-Allyl-sal$_2$323)]NO$_3$·MeCN	*P*2$_1$/*c*	100	25.72	76.93	LS
9a		[Fe^{III}(3-^{t}Bu-sal$_2$323)]PF$_6$·EtOH	*P*2$_1$/*c*	100(I)	21.94	50.17	LS

	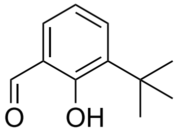			100(II)	24.24	58.49	LS
9b	3-^{t}Bu	[Fe^{III}(3-^{t}Bu-$sal_2$323)]BF_4	$P4_322$	293	19.58	44.76	LS
10a	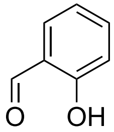	[Fe^{III}($sal_2$323)]NO_3	$P2_1/c$	100	28.27	80.76	LS[4]
10b		[Fe^{III}($sal_2$323)]BPh_4	$P2_1/n$	293	28.43	74.16	LS[5]
10c		[Fe^{III}($sal_2$323)]Cl	$Pccn$	100	30.1	88.4	LS[6]
10d		[Fe^{III}($sal_2$323)]ClO_4	$P2_1/c$	100	27.29	69.38	LS[2]
10e	H	[Fe^{III}($sal_2$323)]$FeCl_4$	$P2_12_12_1$	100	26.02	68.49	LS
10f		[Fe^{III}($sal_2$323)]BF_4	$P2_1/c$	100	25.3	63.64	LS
11a	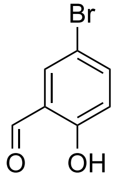	[Fe^{III}(5-Br-$sal_2$323)]PF_6	$P2_1$	293	25.94	65.46	LS
11b		[Fe^{III}(5-Br-$sal_2$323)]BF_4·EtOH	P-1	100	22.38	59.2	LS
11c		[Fe^{III}(5-Br-$sal_2$323)]NO_3·^{i}PrOH	$P2_1/n$	100 (I)	25.95	71.41	LS
	5-Br			100(II)	25.92	68.38	LS
12	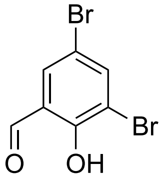 3,5-diBr	[Fe^{III}(3,5-diBr-$sal_2$323)]NO_3·^{i}PrOH	$P2_1/n$	100	24.74	63.01	LS
13a	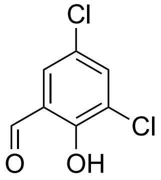	[Fe^{III}(3,5-diCl-$sal_2$323)]BF_4·^{i}PrOH	$P2/n$	293(I)	23.63	60.99	LS
				293(II)	29.9	78.11	LS
13b	3,5-diCl	[Fe^{III}(3,5-diCl-$sal_2$323)]PF_6	$P2_1/n$	100	24.54	60.26	LS

14	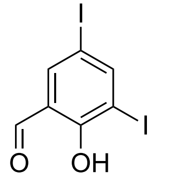 3,5-diI	[Fe^{III}(3,5-diI-sal$_2$323)]PF$_6$	*P*2$_1$/*c*	100	25.7	64.96	LS	
15a	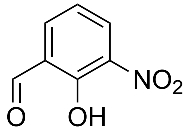 3-NO$_2$	[Fe^{III}(3-NO$_2$-sal$_2$323)]PF$_6$·MeCN	*P*2$_1$/*n*	293	27.96	65.59	LS	
15b		[Fe^{III}(3-NO$_2$-sal$_2$323)]NO$_3$	*Cc*	100	29.94	72.17	LS	
16a	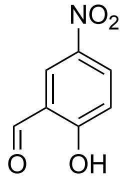 5-NO$_2$	[Fe^{III}(5-NO$_2$-sal$_2$323)]PF$_6$·EtOH	*P*-1	293	24.05	63.44	LS	
16b		[Fe^{III}(5-NO$_2$-sal$_2$323)]BF$_4$·EtOH	*P*-1	293	22.36	58.7	LS	
16c		[Fe^{III}(5-NO$_2$-sal$_2$323)]ClO$_4$·EtOH	*P*-1	100	21.02	56.65	LS	
17	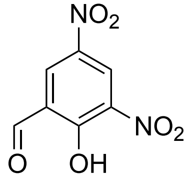 3,5-diNO$_2$	[Fe^{III}(3,5-diNO$_2$-sal$_2$323)]ClO$_4$·EtOH	*P*2$_1$/*n*	100	27.75	76.98	LS	

[a]Refers to the temperature of the diffraction experiment. [b]Structures with more than one independent Fe^{III} site in the asymmetric unit are indicated as (I) and (II). [c]Distortion parameters Σ and Θ are described in the main text. [d]Those structures previously reported in the literature are indicated with the appropriate reference. [e]Where OTf is CF_3SO_3.

S1.1 Synthesis of [Fe^{III}(4-OMe-$sal_2$323)]NO_3 (1a) and [Fe^{III}(4-OMe-$sal_2$323)]NO_3·0.75MeCN·0.25MeOH (1a·S)

4-Methoxysalicylaldehyde (30.4 mg, 0.2 mmol) and 1,2-bis(3-aminopropylamino)ethane (17.4 mg, 0.1 mmol) were briefly stirred in methanol/acetonitrile (1:1, 5 mL), a yellow color was immediately observed. To this solution iron(III) nitrate nonahydrate (40.4 mg, 0.1 mmol) was added, a deep purple color was observed. The solution was briefly stirred to dissolve all solids, and subsequently filtered. Crystals were obtained through slow evaporation of solvent over a week. Elemental analysis, calculated for $C_{24}H_{32}N_5O_7Fe$, Theory % (Found %): C 51.62 (51.49); H 5.78 (5.54); N 12.54 (12.52).

S1.2 Synthesis of [Fe^{III}(4-OMe-$sal_2$323)]PF_6·0.45H_2O (1b)

4-Methoxysalicylaldehyde (30.4 mg, 0.2 mmol) and 1,2-bis(3-aminopropylamino)ethane (17.4 mg, 0.1 mmol) were briefly stirred in methanol (5 mL), a yellow color was immediately observed. To this solution iron(III) chloride (12.7 mg, 0.1 mmol) and potassium hexafluorophosphate (27.6 mg, 0.15 mmol) was added, a deep purple color was observed. The solution was briefly stirred to dissolve all solids, and subsequently filtered. Crystals were obtained through slow evaporation of solvent over a week. Elemental analysis, calculated for $C_{24}H_{32.90}N_4O_{4.45}F_6PFe$, Theory % (Found %): C 44.39 (44.23); H 5.11 (5.11); N 8.63 (8.55).

S1.3 Synthesis of [Fe^{III}(4-OMe-$sal_2$323)]OTf·0.27H_2O (1c)

4-Methoxysalicylaldehyde (30.4 mg, 0.2 mmol) and 1,2-bis(3-aminopropylamino)ethane (17.4 mg, 0.1 mmol) were briefly stirred in methanol (5 mL), a yellow color was immediately observed. To this solution iron(III) chloride (12.7 mg, 0.1 mmol) and lithium trifluoromethanesulfonate (23.4 mg, 0.15 mmol) was added, a deep purple color was observed. The solution was briefly stirred to dissolve all solids, and subsequently filtered. Crystals were obtained through slow evaporation of solvent over a week. Elemental analysis, calculated for $C_{25}H_{32.52}N_4O_{7.26}F_3SFe$, Theory % (Found %): C 46.18 (46.19); H 5.04 (5.01); N 8.62 (8.55).

S1.4 Synthesis of [Fe^{III}(4-OMe-$sal_2$323)]ClO_4 (1d)

4-Methoxysalicylaldehyde (30.4 mg, 0.2 mmol) and 1,2-bis(3-aminopropylamino)ethane (17.4 mg, 0.1 mmol) were briefly stirred in methanol (5 mL), a yellow color was immediately observed. To this solution iron(II) perchlorate hexahydrate (36.3 mg, 0.1 mmol) was added, a deep purple color was observed. The solution was briefly stirred to dissolve all solids, and subsequently filtered. Crystals were obtained through slow evaporation of solvent over a week. Elemental analysis, calculated for $C_{24} H_{32} N_4 O_8$ Cl Fe, Theory % (Found %): C 48.38 (48.16); H 5.41 (5.31); N 9.40 (9.28).

S1.5 Synthesis of [Fe^{III}(4-OMe-$sal_2$323)]BF_4 (1e)

4-Methoxysalicylaldehyde (30.4 mg, 0.2 mmol) and 1,2-bis(3-aminopropylamino)ethane (17.4 mg, 0.1 mmol) were briefly stirred in methanol (5 mL), a yellow color was immediately observed. To this solution iron(II) tetrafluoroborate hexahydrate (33.8 mg, 0.1 mmol) was added, a deep purple color was observed. The solution was briefly stirred to dissolve all solids, and subsequently filtered. Crystals were obtained through slow evaporation of solvent over a week.

Elemental analysis, calculated for $C_{24}H_{32}BN_4O_4F_4Fe$, Theory % (Found %): C 49.43 (49.55); H 5.53 (5.50); N 9.61 (9.44).

S1.6 Synthesis of [Fe^{III}(4-OMe-$sal_2$323)]SbF_6·0.31H_2O (1f)

4-Methoxysalicylaldehyde (30.4 mg, 0.2 mmol) and 1,2-bis(3-aminopropylamino)ethane (17.4 mg, 0.1 mmol) were briefly stirred in methanol (5 mL), a yellow color was immediately observed. To this solution iron(III) chloride (12.7 mg, 0.1 mmol) and sodium hexafluoroantimonate (38.9 mg, 0.15 mmol) was added, a deep purple color was observed. The solution was briefly stirred to dissolve all solids, and subsequently filtered. Crystals were obtained through slow evaporation of solvent over a week. Elemental analysis, calculated for $C_{24}H_{32.62}N_4O_{4.31}FeF_6Sb$, Theory % (Found %): C 39.08 (37.97); H 4.46 (4.41); N 7.59 (7.55).

S1.7 Synthesis of [Fe^{III}(4-OMe-$sal_2$323)]I_3 (1g)

4-Methoxysalicylaldehyde (30.4 mg, 0.2 mmol) and 1,2-bis(3-aminopropylamino)ethane (17.4 mg, 0.1 mmol) were briefly stirred in methanol (5 mL), a yellow color was immediately observed. To this solution iron(III) chloride (12.7 mg, 0.1 mmol) and sodium iodide (22.5 mg, 0.15 mmol) was added, a deep purple color was observed. The solution was briefly stirred to dissolve all solids, and subsequently filtered. Crystals were obtained through slow evaporation of solvent over a week. Too few crystals were obtained to carry out elemental analysis on the bulk sample.

S1.8 Synthesis of [Fe^{III}(4-OMe-$sal_2$323)]Cl·EtOH·0.25H_2O (1h)

4-Methoxysalicylaldehyde (30.4 mg, 0.2 mmol) and 1,2-bis(3-aminopropylamino)ethane (17.4 mg, 0.1 mmol) were briefly stirred in methanol (5 mL), a yellow color was immediately observed. To this solution iron(III) chloride (12.7 mg, 0.1 mmol) was added, a deep purple color was observed. The solution was briefly stirred to dissolve all solids, and subsequently filtered. Crystals were obtained through slow evaporation of solvent over a week. Too few crystals were obtained to carry out elemental analysis on the bulk sample.

S1.9 Synthesis of [Fe^{III}(3-OMe-$sal_2$323)]NO_3 (2a)

3-Methoxysalicylaldehyde (30.4 mg, 0.2 mmol) and 1,2-bis(3-aminopropylamino)ethane (17.4 mg, 0.1 mmol) were briefly stirred in methanol (5 mL), a yellow color was immediately observed. To this solution iron(III) nitrate nonahydrate (40.4 mg, 0.1 mmol) was added, a deep purple color was observed. The solution was briefly stirred to dissolve all solids, and subsequently filtered. Crystals were obtained through slow evaporation of solvent over a week. Elemental analysis, calculated for $C_{24}H_{32}N_5O_7Fe$, Theory % (Found %): C 51.62 (50.71); H 5.78 (5.48); N 12.54 (12.11).

S1.10 Synthesis of [Fe^{III}(3-OMe-$sal_2$323)]BF_4·H_2O (2b)

3-Methoxysalicylaldehyde (304 mg, 2 mmol) and 1,2-bis(3-aminopropylamino)ethane (174 mg, 1 mmol) were briefly stirred in IMS and acetonitrile (1:1, 20 mL), a yellow color was immediately observed. To this solution iron(II) tetrafluoroborate hexahydrate (338 mg, 1 mmol) was added, a deep purple color was observed. The solution was briefly stirred to dissolve all solids, and subsequently filtered. Crystals were obtained through slow evaporation of

solvent over a week. Elemental analysis, calculated for $C_{24}H_{34}BN_4O_5F_4Fe$, Theory % (Found %): C 47.95 (48.06); H 5.70 (5.58); N 9.32 (9.28).

S1.11 Synthesis of [Fe^{III}(3-OMe-sal$_2$323)]PF$_6$·H$_2$O (2c)

3-Methoxysalicylaldehyde (304 mg, 2 mmol) and 1,2-bis(3-aminopropylamino)ethane (174 mg, 1 mmol) were briefly stirred in IMS and acetonitrile (1:1, 20 mL), a yellow color was immediately observed. To this solution iron(III) chloride (162 mg, 1 mmol) and ammonium hexafluorophosphate (163 mg, 1 mmol) was added, a deep purple color was observed. The solution was briefly stirred to dissolve all solids, and subsequently filtered. Crystals were obtained through slow evaporation of solvent over a week. Elemental analysis, calculated for $C_{24}H_{34}N_4O_5F_6PFe$, Theory % (Found %): C 43.72 (43.60); H 5.20 (5.02); N 8.50 (8.68).

S1.12 Synthesis of [Fe^{III}(5-OMe-sal$_2$323)]NO$_3$ (3a)

5-Methoxysalicylaldehyde (30.4 mg, 0.2 mmol) and 1,2-bis(3-aminopropylamino)ethane (17.4 mg, 0.1 mmol) were briefly stirred in methanol (5 mL), a yellow color was immediately observed. To this solution iron(III) nitrate nonahydrate (40.4 mg, 0.1 mmol) was added, a deep purple color was observed. The solution was briefly stirred to dissolve all solids, and subsequently filtered. Crystals were obtained through slow evaporation of solvent over a week. Elemental analysis, calculated for $C_{24}H_{32}N_5O_7Fe$, Theory % (Found %): C 51.62 (51.38); H 5.78 (5.73); N 12.54 (12.38).

S1.13 Synthesis of [Fe^{III}(5-OMe-sal$_2$323)]BF$_4$ (3b)

5-Methoxysalicylaldehyde (304 mg, 2 mmol) and 1,2-bis(3-aminopropylamino)ethane (174 mg, 1 mmol) were briefly stirred in IMS and acetonitrile (1:1, 20 mL), a yellow color was immediately observed. To this solution iron(III) nitrate nonahydrate (404 mg, 1 mmol) was added, a deep purple color was observed. The solution was briefly stirred to dissolve all solids, and subsequently filtered. Crystals were obtained through slow evaporation of solvent over a week. Elemental analysis, calculated for $C_{24}H_{32}BN_4O_4F_4Fe$, Theory % (Found %): C 49.43 (47.60); H 5.53 (5.34); N 9.61 (9.43).

S1.14 Synthesis of [Fe^{III}(4,6-diOMe-sal$_2$323)]NO$_3$·MeOH (4a)

4,6-Dimethoxysalicylaldehyde (36.4 mg, 0.2 mmol) and 1,2-bis(3-aminopropylamino)ethane (17.4 mg, 0.1 mmol) were briefly stirred in methanol (10 mL), a yellow color was immediately observed. To this solution iron(III) nitrate nonahydrate (40.4 mg, 0.1 mmol) was added, a deep red color was observed. The solution was briefly stirred to dissolve all solids, and subsequently filtered. Crystals were obtained through slow evaporation of solvent over a week. Too few crystals were obtained to carry out elemental analysis on the bulk sample.

S1.15 Synthesis of [Fe^{III}(4,6-diOMe-sal$_2$323)]BF$_4$·0.5MeOH (4b)

4,6-Dimethoxysalicylaldehyde (36.4 mg, 0.2 mmol) and 1,2-bis(3-aminopropylamino)ethane (17.4 mg, 0.1 mmol) were briefly stirred in methanol (10 mL), a yellow color was immediately observed. To this solution iron(II) tetrafluoroborate hexahydrate (33.8 mg, 0.1 mmol) was added, a deep red color was observed. The solution was briefly

stirred to dissolve all solids, and subsequently filtered. Crystals were obtained through slow evaporation of solvent over a week. Too few crystals were obtained to carry out elemental analysis on the bulk sample.

S1.16 Synthesis of [Fe^{III}(4,6-diOMe-$sal_2$323)]ClO_4·0.5MeOH (4c)

4,6-Dimethoxysalicylaldehyde (36.4 mg, 0.2 mmol) and 1,2-bis(3-aminopropylamino)ethane (17.4 mg, 0.1 mmol) were briefly stirred in methanol (10 mL), a yellow color was immediately observed. To this solution iron(II) perchlorate hexahydrate (36.3 mg, 0.1 mmol) was added, a deep red color was observed. The solution was briefly stirred to dissolve all solids, and subsequently filtered. Crystals were obtained through slow evaporation of solvent over a week. Too few crystals were obtained to carry out elemental analysis on the bulk sample.

S1.17 Synthesis of [Fe^{III}(3-OEt-$sal_2$323)]PF_6·EtOH (5a)

3-Ethoxysalicylaldehyde (33.2 mg, 0.2 mmol) and 1,2-bis(3-aminopropylamino)ethane (17.4 mg, 0.1 mmol) were briefly stirred in methanol (10 mL), a yellow color was immediately observed. To this solution iron(III) chloride (12.7 mg, 0.1 mmol) and potassium hexafluorophosphate (27.6 mg, 0.15 mmol) was added, a deep red color was observed. The solution was briefly stirred to dissolve all solids, and subsequently filtered. Crystals were obtained through slow evaporation of solvent over a week. Too few crystals were obtained to carry out elemental analysis on the bulk sample.

S1.18 Synthesis of [Fe^{III}(3-OEt-$sal_2$323)]BF_4·0.32H_2O (5b)

3-Ethoxysalicylaldehyde (33.2 mg, 0.2 mmol) and 1,2-bis(3-aminopropylamino)ethane (17.4 mg, 0.1 mmol) were briefly stirred in methanol (10 mL), a yellow color was immediately observed. To this solution iron(II) tetrafluoroborate hexahydrate (33.8 mg, 0.1 mmol) was added, a deep red color was observed. The solution was briefly stirred to dissolve all solids, and subsequently filtered. Crystals were obtained through slow evaporation of solvent over a week. Too few crystals were obtained to carry out elemental analysis on the bulk sample.

S1.19 Synthesis of [Fe^{III}(4-Et_2N-$sal_2$323)]PF_6 (6b)

4-(Diethylamino)salicylaldehyde (38.6 mg, 0.2 mmol) and 1,2-bis(3-aminopropylamino)ethane (17.4 mg, 0.1 mmol) were briefly stirred in methanol (5 mL), a yellow color was immediately observed. To this solution iron(III) chloride (12.7 mg, 0.1 mmol) and sodium hexafluorophosphate (27.6 mg, 0.15 mmol) was added, a deep purple color was observed. The solution was briefly stirred to dissolve all solids, and subsequently filtered. Crystals were obtained through slow evaporation of solvent over a week. Elemental analysis, calculated for $C_{30}H_{46}N_6O_2F_6PFe$, Theory % (Found %): C 49.80 (49.79); H 6.41 (6.45); N 11.62 (11.50).

S1.20 Synthesis of [Fe^{III}(4-Et_2N-$sal_2$323)]PF_6·0.78MeOH·0.1EtOH (6b·S)

4-(Diethylamino)salicylaldehyde (38.6 mg, 0.2 mmol) and 1,2-bis(3-aminopropylamino)ethane (17.4 mg, 0.1 mmol) were briefly stirred in ethanol (5 mL), a yellow color was immediately observed. To this solution iron(III) chloride (12.7 mg, 0.1 mmol) and sodium hexafluorophosphate (27.6 mg, 0.15 mmol) was added, a deep purple color was observed. The solution was refluxed at 110 °C for 3 hours, and subsequently filtered. The solvent was removed by

rotary evaporation. Recrystallisation of the crude solid in the minimum acetonitrile/ethanol (1:1) yielded crystals suitable for SCXRD. Too few crystals were obtained to carry out elemental analysis on the bulk sample.

S1.21 Synthesis of [Fe^{III}(4-Et_2N-$sal_2$323)]OTf (6c)

4-(Diethylamino)salicylaldehyde (38.6 mg, 0.2 mmol) and 1,2-bis(3-aminopropylamino)ethane (17.4 mg, 0.1 mmol) were briefly stirred in methanol (5 mL), a yellow color was immediately observed. To this solution iron(III) chloride (12.7 mg, 0.1 mmol) and lithium trifluoromethanesulfonate (23.4 mg, 0.15 mmol) was added, a deep purple color was observed. The solution was briefly stirred to dissolve all solids, and subsequently filtered. Crystals were obtained through slow evaporation of solvent over a week. Elemental analysis, calculated for $C_{31}H_{46}N_6O_5F_3SFe$, Theory % (Found %): C 51.17 (51.07); H 6.37 (6.41); N 11.55 (11.43).

S1.22 Synthesis of [Fe^{III}(4-Et_2N-$sal_2$323)]BF_4 (6d)

4-(Diethylamino)salicylaldehyde (38.6 mg, 0.2 mmol) and 1,2-bis(3-aminopropylamino)ethane (17.4 mg, 0.1 mmol) were briefly stirred in methanol (5 mL), a yellow color was immediately observed. To this solution iron(II) tetrafluoroborate hexahydrate (33.8 mg, 0.1 mmol) was added, a deep purple color was observed. The solution was briefly stirred to dissolve all solids, and subsequently filtered. Crystals were obtained through slow evaporation of solvent over a week. Elemental analysis, calculated for $C_{30}H_{46}BN_6O_2F_4Fe$, Theory % (Found %): C 54.15 (54.27); H 6.97 (6.98); N 12.63 (12.56).

S1.23 Synthesis of [Fe^{III}(4-Et_2N-$sal_2$323)]BF_4·EtOH (6d·S)

4-(Diethylamino)salicylaldehyde (38.6 mg, 0.2 mmol) and 1,2-bis(3-aminopropylamino)ethane (17.4 mg, 0.1 mmol) were briefly stirred in ethanol (5 mL), a yellow color was immediately observed. To this solution iron(II) tetrafluoroborate hexahydrate (33.8 mg, 0.1 mmol) was added, a deep purple color was observed. The solution was refluxed at 110 °C for 3 hours, and subsequently filtered. The solvent was removed by rotary evaporation. Recrystallisation of the crude solid in the minimum acetonitrile/ethanol (1:1) yielded crystals suitable for SCXRD. Elemental analysis, calculated for $C_{32}H_{52}BN_6O_3F_4Fe$, Theory % (Found %): C 54.02 (53.90); H 7.37 (6.97); N 11.81 (11.93).

S1.24 Synthesis of [Fe^{III}(4-Et_2N-$sal_2$323)]NO_3·CH_2Cl_2 (6e)

4-(Diethylamino)salicylaldehyde (193 mg, 1 mmol) and 1,2-bis(3-aminopropylamino)ethane (87 mg, 0.5 mmol) were briefly stirred in acetonitrile (20 mL), a yellow color was immediately observed. To this solution iron(III) nitrate nonahydrate (202 mg, 0.5 mmol) was added, a deep purple color was observed. The solution was briefly stirred to dissolve all solids, and subsequently filtered. A crude powder was collected which was recrystallized in dichloromethane. Crystals were obtained through slow evaporation of solvent over a week. Elemental analysis, calculated for $C_{30}H_{46}N_7O_5Fe$, Theory % (Found %): C 56.25 (55.91); H 7.24 (7.54); N 15.31 (14.37).

S1.25 Synthesis of [Fe^{III}(3-Me-$sal_2$323)]ClO_4 (7a)

3-Methylsalicylaldehyde (27.2 mg, 0.2 mmol) and 1,2-bis(3-aminopropylamino)ethane (17.4 mg, 0.1 mmol) were briefly stirred in methanol (5 mL), a yellow color was immediately observed. To this solution iron(II) perchlorate hexahydrate (36.3 mg, 0.1 mmol) was added, a deep purple color was observed. The solution was briefly stirred to dissolve all solids, and subsequently filtered. Crystals were obtained through slow evaporation of solvent over a week. Too few crystals were obtained to carry out elemental analysis on the bulk sample.

S1.26 Synthesis of [Fe^{III}(3-Me-$sal_2$323)]PF_6·0.68H_2O (7b)

3-Methylsalicylaldehyde (27.2 mg, 0.2 mmol) and 1,2-bis(3-aminopropylamino)ethane (17.4 mg, 0.1 mmol) were briefly stirred in methanol (5 mL), a yellow color was immediately observed. To this solution iron(III) chloride (12.7 mg, 0.1 mmol) and potassium hexafluorophosphate (27.6 mg, 0.15 mmol) was added, a deep purple color was observed. The solution was briefly stirred to dissolve all solids, and subsequently filtered. Crystals were obtained through slow evaporation of solvent over a week. $C_{24}H_{33.36}N_4O_{2.68}F_6PFe$, Theory % (Found %): C 46.37 (46.74); H 5.41 (5.66); N 9.01 (8.75).

S1.27 Synthesis of [Fe^{III}(3-Me-$sal_2$323)]BF_4 (7c)

3-Methylsalicylaldehyde (27.2 mg, 0.2 mmol) and 1,2-bis(3-aminopropylamino)ethane (17.4 mg, 0.1 mmol) were briefly stirred in methanol (5 mL), a yellow color was immediately observed. To this solution iron(II) tetrafluoroborate hexahydrate (33.8 mg, 0.1 mmol) was added, a deep purple color was observed. The solution was briefly stirred to dissolve all solids, and subsequently filtered. Crystals were obtained through slow evaporation of solvent over a week. Too few crystals were obtained to carry out elemental analysis on the bulk sample.

S1.28 Synthesis of [Fe^{III}(3-Allyl-$sal_2$323)]NO_3·MeCN (8)

3-Allylsalicylaldehyde (32.4 mg, 0.2 mmol) and 1,2-bis(3-aminopropylamino)ethane (17.4 mg, 0.1 mmol) were briefly stirred in IMS and acetonitrile (1:1, 5 mL), a yellow color was immediately observed. To this solution iron(III) nitrate nonahydrate (40.4 mg, 0.1 mmol) was added, a deep purple color was observed. The solution was briefly stirred to dissolve all solids, and subsequently filtered. Crystals were obtained through slow evaporation of solvent over a week. Too few crystals were obtained to carry out elemental analysis on the bulk sample.

S1.29 Synthesis of [Fe^{III}(3-^{t}Bu-$sal_2$323)]PF_6·EtOH (9a)

3-Tert-butyl salicylaldehyde (356 mg, 2 mmol) and 1,2-bis(3-aminopropylamino)ethane (174 mg, 1 mmol) were briefly stirred in IMS and acetonitrile (1:1, 20 mL), a yellow color was immediately observed. To this solution iron(III) chloride (162 mg, 1 mmol) and ammonium hexafluorophosphate (163 mg, 1 mmol) was added, a deep purple color was observed. The solution was briefly stirred to dissolve all solids, and subsequently filtered. Crystals were obtained through slow evaporation of solvent over a week. Elemental analysis, calculated for $C_{31}H_{47}N_4O_{2.5}F_6PFe$, Theory % (Found %): C 51.96 (51.23); H 6.61 (6.59); N 7.82 (8.19).

S1.30 Synthesis of [Fe^{III}(3-^{t}Bu-sal$_2$323)]BF_4 (9b)

3-Tert-butyl salicylaldehyde (356 mg, 2 mmol) and 1,2-bis(3-aminopropylamino)ethane (174 mg, 1 mmol) were briefly stirred in IMS and acetonitrile (1:1, 20 mL), a yellow color was immediately observed. To this solution iron(II) tetrafluoroborate hexahydrate (338 mg, 1 mmol) was added, a deep purple color was observed. The solution was briefly stirred to dissolve all solids, and subsequently filtered. Crystals were obtained through slow evaporation of solvent over a week. Elemental analysis, calculated for $C_{30}H_{44}N_4O_2F_4BFe$, Theory % (Found %): C 56.71 (55.03); H 6.98 (7.08); N 8.82 (8.77).

S1.31 Synthesis of [Fe^{III}(sal$_2$323)]$FeCl_4$ (10e)

Salicylaldehyde (244 mg, 2 mmol) and 1,2-bis(3-aminopropylamino)ethane (174 mg, 1 mmol) were briefly stirred in methanol (5 mL), a yellow color was immediately observed. To this solution iron(III) chloride (162 mg, 1 mmol) was added, a deep purple color was observed. The solution was briefly stirred to dissolve all solids, and subsequently filtered. Crystals were obtained through slow evaporation of solvent over a week. Too few crystals were obtained to carry out elemental analysis on the bulk sample.

S1.32 Synthesis of [Fe^{III}(sal$_2$323)]BF_4 (10f)

Salicylaldehyde (24.4 mg, 0.2 mmol) and 1,2-bis(3-aminopropylamino)ethane (17.4 mg, 0.1 mmol) were briefly stirred in methanol (5 mL), a yellow color was immediately observed. To this solution iron(II) tetrafluoroborate hexahydrate (33.8 mg, 0.1 mmol) was added, a deep purple color was observed. The solution was briefly stirred to dissolve all solids, and subsequently filtered. Crystals were obtained through slow evaporation of solvent over a week. Too few crystals were obtained to carry out elemental analysis on the bulk sample.

S1.33 Synthesis of [Fe^{III}(5-Br-sal$_2$323)]PF_6 (11a)

5-Bromosalicylaldehyde (402 mg, 2 mmol) and 1,2-bis(3-aminopropylamino)ethane (174 mg, 1 mmol) were briefly stirred in IMS and acetonitrile (1:1, 20 mL), a yellow color was immediately observed. To this solution iron(III) chloride (162 mg, 1 mmol) and ammonium hexafluorophosphate (163 mg, 1 mmol) was added, a deep purple color was observed. The solution was briefly stirred to dissolve all solids, and subsequently filtered. Crystals were obtained through slow evaporation of solvent over a week. Elemental analysis, calculated for $C_{22}H_{26}N_4O_2F_6PFeBr_2$, Theory % (Found %): C 35.75 (36.63); H 3.55 (4.06); N 7.58 (7.10).

S1.34 Synthesis of [Fe^{III}(5-Br-sal$_2$323)]BF_4·EtOH (11b)

5-Bromosalicylaldehyde (402 mg, 2 mmol) and 1,2-bis(3-aminopropylamino)ethane (174 mg, 1 mmol) were briefly stirred in IMS and acetonitrile (1:1, 20 mL), a yellow color was immediately observed. To this solution iron(II) tetrafluoroborate hexahydrate (338 mg, 1 mmol) was added, a deep purple color was observed. The solution was briefly stirred to dissolve all solids, and subsequently filtered. Crystals were obtained through slow evaporation of solvent over a week. Elemental analysis, calculated for $C_{24}H_{32}N_4O_3BF_4FeBr_2$, Theory % (Found %): C 39.65 (38.07); H 4.44 (3.91); N 7.71 (7.60).

S1.35 Synthesis of [Fe^{III}(5-Br-sal$_2$323)]NO_3·^{i}PrOH (11c)

5-Bromosalicylaldehyde (402 mg, 2 mmol) and 1,2-bis(3-aminopropylamino)ethane (174 mg, 1 mmol) were briefly stirred in IMS and acetonitrile (1:1, 20 mL), a yellow color was immediately observed. To this solution iron(III) nitrate nonahydrate (404 mg, 1 mmol) was added, a deep purple color was observed. The solution was briefly stirred to dissolve all solids, and subsequently filtered. Crystals were obtained through slow evaporation of solvent over a week. Elemental analysis, calculated for $C_{25}H_{34}N_5O_6FeBr_2$, Theory % (Found %): C 41.92 (41.84); H 4.50 (4.33); N 9.78 (7.90).

S1.36 Synthesis of [Fe^{III}(3,5-diBr-sal$_2$323)]NO_3·^{i}PrOH (12)

3,5-Dibromosalicylaldehyde (560 mg, 2 mmol) and 1,2-bis(3-aminopropylamino)ethane (174 mg, 1 mmol) were briefly stirred in IMS and acetonitrile (1:1, 20 mL), a yellow color was immediately observed. To this solution iron(III) nitrate nonahydrate (404 mg, 1 mmol) was added, a deep purple color was observed. The solution was briefly stirred to dissolve all solids, and subsequently filtered. Crystals were obtained through slow evaporation of solvent over a week. Elemental analysis, calculated for $C_{25}H_{32}N_5O_6FeBr_4$, Theory % (Found %): C 34.36 (33.15); H 3.69 (3.44); N 8.01 (8.02).

S1.37 Synthesis of [Fe^{III}(3,5-diCl-sal$_2$323)]BF_4·^{i}PrOH (13a)

3,5-Dichlorosalicylaldehyde (382 mg, 2 mmol) and 1,2-bis(3-aminopropylamino)ethane (174 mg, 1 mmol) were briefly stirred in IMS and acetonitrile (1:1, 20 mL), a yellow color was immediately observed. To this solution iron(II) tetrafluoroborate hexahydrate (338 mg, 1 mmol) was added, a deep purple color was observed. The solution was briefly stirred to dissolve all solids, and subsequently filtered. Crystals were obtained through slow evaporation of solvent over a week. Elemental analysis, calculated for $C_{25}H_{32}BN_4O_3F_4Cl_4Fe$, Theory % (Found %): C 41.65 (41.27); H 4.47 (4.54); N 7.77 (7.72).

S1.38 Synthesis of [Fe^{III}(3,5-diCl-sal$_2$323)]PF_6 (13b)

3,5-Dichlorosalicylaldehyde (382 mg, 2 mmol) and 1,2-bis(3-aminopropylamino)ethane (17.4 mg, 0.1 mmol) were briefly stirred in IMS and acetonitrile (1:1, 20 mL), a yellow color was immediately observed. To this solution iron(III) chloride (162 mg, 1 mmol) and ammonium hexafluorophosphate (163 mg, 1 mmol) was added, a deep purple color was observed. The solution was briefly stirred to dissolve all solids, and subsequently filtered. Crystals were obtained through slow evaporation of solvent over a week. Elemental analysis, calculated for $C_{22}H_{24}N_4O_2F_6PCl_4Fe$, Theory % (Found %): C 36.75 (37.58); H 3.36 (4.47); N 7.79 (9.93).

S1.39 Synthesis of [Fe^{III}(3,5-diI-sal$_2$323)]PF_6 (14)

3,5-Diiodosalicylaldehyde (748 mg, 2 mmol) and 1,2-bis(3-aminopropylamino)ethane (174 mg, 1 mmol) were briefly stirred in IMS and acetonitrile (1:1, 20 mL), a yellow color was immediately observed. To this solution iron(III) chloride (162 mg, 1 mmol) and ammonium hexafluorophosphate (163 mg, 1 mmol) was added, a deep purple color was observed. The solution was briefly stirred to dissolve all solids, and subsequently filtered. Crystals were obtained

through slow evaporation of solvent over a week. Elemental analysis, calculated for $C_{22}H_{24}N_4O_2F_6PFeI_4$, Theory % (Found %): C 24.36 (24.54); H 2.23 (2.28); N 5.16 (4.93).

S1.40 Synthesis of [Fe^{III}(3-NO$_2$-sal$_2$323)]PF$_6$·MeCN (15a)

3-Nitrosalicylaldehyde (334 mg, 2 mmol) and 1,2-bis(3-aminopropylamino)ethane (174 mg, 1 mmol) were briefly stirred in IMS and acetonitrile (1:1, 20 mL), a yellow color was immediately observed. To this solution iron(III) chloride (162 mg, 1 mmol) and ammonium hexafluorophosphate (163 mg, 1 mmol) was added, a deep purple color was observed. The solution was briefly stirred to dissolve all solids, and subsequently filtered. Crystals were obtained through slow evaporation of solvent over a week. Elemental analysis, calculated for $C_{24}H_{29}N_7O_6F_6PFe$, Theory % (Found %): C 40.47 (40.29); H 4.10 (4.06); N 13.76 (13.44).

S1.41 Synthesis of [Fe^{III}(3-NO$_2$-sal$_2$323)]NO$_3$ (15b)

3-Nitrosalicylaldehyde (334 mg, 2 mmol) and 1,2-bis(3-aminopropylamino)ethane (174 mg, 1 mmol) were briefly stirred in IMS and acetonitrile (1:1, 20 mL), a yellow color was immediately observed. To this solution iron(III) nitrate nonahydrate (404 mg, 1 mmol) was added, a deep purple color was observed. The solution was briefly stirred to dissolve all solids, and subsequently filtered. Crystals were obtained through slow evaporation of solvent over a week. Elemental analysis, calculated for $C_{22}H_{26}N_7O_9Fe$, Theory % (Found %): C 44.91 (44.02); H 4.45 (4.46); N 16.67 (16.75).

S1.42 Synthesis of [Fe^{III}(5-NO$_2$-sal$_2$323)]PF$_6$·EtOH (16a)

5-Nitrosalicylaldehyde (334 mg, 2 mmol) and 1,2-bis(3-aminopropylamino)ethane (174 mg, 1 mmol) were briefly stirred in IMS and acetonitrile (1:1, 20 mL), a yellow color was immediately observed. To this solution iron(III) chloride (162 mg, 1 mmol) and ammonium hexafluorophosphate (163 mg, 1 mmol) was added, a deep purple color was observed. The solution was briefly stirred to dissolve all solids, and subsequently filtered. Crystals were obtained through slow evaporation of solvent over a week. Elemental analysis, calculated for $C_{24}H_{32}N_6O_7F_6PFe$, Theory % (Found %): C 40.18 (40.06); H 4.50 (4.38); N 11.72 (11.76).

S1.43 Synthesis of [Fe^{III}(5-NO$_2$-sal$_2$323)]BF$_4$·EtOH (16b)

5-Nitrosalicylaldehyde (334 mg, 2 mmol) and 1,2-bis(3-aminopropylamino)ethane (174 mg, 1 mmol) were briefly stirred in IMS and acetonitrile (1:1, 20 mL), a yellow color was immediately observed. To this solution iron(II) tetrafluoroborate hexahydrate (338 mg, 1 mmol) was added, a deep purple color was observed. The solution was briefly stirred to dissolve all solids, and subsequently filtered. Crystals were obtained through slow evaporation of solvent over a week. Elemental analysis, calculated for $C_{24}H_{32}BN_6O_7F_4Fe$, Theory % (Found %): C 43.75 (43.60); H 4.89 (4.76); N 12.75 (12.68).

S1.44 Synthesis of [Fe^{III}(5-NO$_2$-sal$_2$323)]ClO$_4$·EtOH (16c)

5-Nitrosalicylaldehyde (33.4 mg, 0.2 mmol) and 1,2-bis(3-aminopropylamino)ethane (17.4 mg, 0.1 mmol) were briefly stirred in methanol (5 mL), a yellow color was immediately observed. To this solution iron(II) perchlorate

hydrate (36.3 mg, 0.1 mmol) was added, a deep purple color was observed. The solution was briefly stirred to dissolve all solids, and subsequently filtered. Crystals were obtained through slow evaporation of solvent over a week. Too few crystals were obtained to carry out elemental analysis on the bulk sample.

S1.45 Synthesis of [Fe^{III}(3,5-NO_2-$sal_2$323)]ClO_4·EtOH (17)

3,5-Dinitrosalicylaldehyde (42.4 mg, 0.2 mmol) and 1,2-bis(3-aminopropylamino)ethane (17.4 mg, 0.1 mmol) were briefly stirred in methanol (5 mL), a yellow color was immediately observed. To this solution iron(II) perchlorate hydrate (36.3 mg, 0.1 mmol) was added, a deep purple color was observed. The solution was briefly stirred to dissolve all solids, and subsequently filtered. Crystals were obtained through slow evaporation of solvent over a week. Too few crystals were obtained to carry out elemental analysis on the bulk sample.

S2 Single Crystal X-ray Diffraction Details

Table S2.1. Crystallographic details for **1** – **17**.

Complex	1a	1a	1a·S	1a·S	1b
**CCDC No.**	2166978	2166979	2166980	2166981	2166982
**Molecular Formula**	$[C_{24}H_{32}N_4O_4Fe]^{+}[NO_3]^{-}$	$[C_{24}H_{32}N_4O_4Fe]^{+}[NO_3]^{-}$	$[C_{24}H_{32}N_4O_4Fe]^{+}[NO_3]^{-}$ x 0.75 (C_2H_3N) x 0.25 (CH_4O)	$[C_{24}H_{32}N_4O_4Fe]^{+}[NO_3]^{-}$ x 0.73 (C_2H_3N) x 0.27 (CH_4O)	$[C_{24}H_{32}N_4O_4Fe]^{+}[F_6P]^{-}$ x 0.45 (H_2O)
$\boldsymbol{M_r}$ (g mol^{-1})	558.39	558.39	597.24	596.99	649.42
$\boldsymbol{T}$ (K)	100(2)	293(2)	100(2)	293(2)	100(2)
**Crystal System**	Orthorhombic	Orthorhombic	Monoclinic	Monoclinic	Monoclinic
**Space Group**	$P2_12_12$ (#18)	$P2_12_12$ (#18)	$P2_1/n$ (#14)	$P2_1/n$ (#14)	$P2_1/c$ (#14)
$\boldsymbol{a}$ (Å)	7.5176(4)	7.6624(2)	7.50006(6)	7.60475(5)	7.99259(9)
$\boldsymbol{b}$ (Å)	12.1717(8)	12.2069(3)	17.0390(2)	17.0965(2)	20.8108(2)
$\boldsymbol{c}$ (Å)	13.336(1)	13.5167(4)	21.7744(2)	22.0910(2)	16.0625(2)
$\boldsymbol{\alpha}$ (°)	90	90	90	90	90
$\boldsymbol{\beta}$ (°)	90	90	98.4035(8)	99.3507(6)	96.781(1)
$\boldsymbol{\gamma}$ (°)	90	90	90	90	90
$\boldsymbol{V}$ (Å^3)	1220.27(14)	1264.27(6)	2752.75(5)	2833.99(5)	2653.02(5)
Z, Z'	2, 0.5	2, 0.5	4, 1	4, 1	4, 1
**Radiation Type**	Cu *Ka*	Cu *Ka*	Cu $K\alpha$	Cu $K\alpha$	Cu $K\alpha$
$\boldsymbol{\mu}$ (mm^{-1})	5.437	5.248	4.873	4.734	5.894
**Crystal Size**	0.080 × 0.050 × 0.020	0.086 × 0.036 × 0.031	0.295 × 0.120 × 0.110	0.162 × 0.127 × 0.111	0.295 × 0.162 × 0.043
**Reflections Measured, Independent Reflections**	5887, 2536	6555, 2628	56379, 5796	35189, 5944	31711, 5554
$\boldsymbol{R_{int}}$	0.0457	0.0334	0.0382	0.0202	0.0324
**Data / Restraints / Parameters**	2536 / 0 / 184	2628 / 0 / 171	5796 / 2 / 374	5944 / 4 / 374	5554 / 0 / 421
**GooF on F^2**	1.033	1.029	1.047	1.051	1.042
**Final *R* Indices [*I>2s(I)*]**	R_1 = 0.0372, wR_2 = 0.0882	R_1 = 0.0394, wR_2 = 0.1046	R_1 = 0.0318, wR_2 = 0.0852	R_1 = 0.0312, wR_2 = 0.0877	R_1 = 0.0337, wR_2 = 0.0851
$\boldsymbol{\Delta\rho_{max}, \Delta\rho_{min}}$ (e Å^{-3})	0.278, -0.340	0.299, -0.339	0.832, -0.482	0.397, -0.311	0.475, -0.502
**Flack Parameter**	0.511(8)[a]	0.502(9)[a]	---	---	---

Complex	1c	1d	1d	1e	1f
**CCDC**	2166983	2166984	2166985	2166986	2166987
**Molecular Formula**	$[C_{24} H_{32} N_4 O_4 Fe]^+[C O_3 F_3 S]^-$ x 0.265 (H_2 O)	$[C_{24} H_{32} N_4 O_4 Fe]^+[Cl O_4]^-$	$[C_{24} H_{32} N_4 O_4 Fe]^+[Cl O_4]^-$	$[C_{24} H_{32} N_4 O_4 Fe]^+[B F_4]^-$	$[C_{24} H_{32} N_4 O_4 Fe]^+[F_6 Sb]^-$ x 0.31 (H_2 O)
M_r (g mol^{-1})	650.23	595.83	595.83	649.42	737.63
T (K)	100(2)	100(2)	200(2)	293(2)	100(2)
**Crystal System**	Monoclinic	Monoclinic	Monoclinic	Monoclinic	Monoclinic
**Space Group**	$P2_1/c$ (#14)	$P2_1/c$ (#14)	$P2_1/c$ (#14)	$P2_1/c$ (#14)	$P2_1/c$ (#14)
a (Å)	8.05017(5)	7.39852(9)	7.47123(5)	7.4540(9)	8.13318(5)
b (Å)	20.9777(2)	16.1939(2)	17.5694(2)	17.537(2)	21.0366(2)
c (Å)	16.2883(1)	21.4358(3)	20.5528(2)	20.521(3)	16.0425(1)
α (°)	90	90	90	90	90
β (°)	97.8371(6)	95.064(1)	96.1464(6)	96.344(2)	96.3820(5)
γ (°)	90	90	90	90	90
V (Å^3)	2724.98(4)	2558.22(6)	2682.35(4)	1640.96(2)	2727.77(4)
**Z, Z'**	4, 1	4, 1	4, 1	4, 1	4, 1
**Radiation Type**	Cu $K\alpha$	Cu $K\alpha$	Cu $K\alpha$	Mo Ka	Cu Ka
μ (mm^{-1})	5.818	6.183	5.897	0.631	12.856
**Crystal Size**	0.193 × 0.118 × 0.071	0.207 × 0.104 × 0.045	0.252 × 0.149 × 0.114	0.40 × 0.10 × 0.10	0.119 × 0.085 × 0.028
**Reflections Measured, Independent Reflections**	55717, 5729	26655, 5372	54881, 5654	14397, 3260	37818, 5744
R_{int}	0.0543	0.0354	0.0309	0.0347	0.0327
**Data / Restraints / Parameters**	5729 / 0 / 394	5372 / 0 / 345	5654 / 0 / 364	3260 / 0 / 354	5744 / 0 / 404
**GooF on F²**	1.066	1.037	1.075	1.031	1.040
**Final *R* Indices [*I>2s(I)*]**	R_1 = 0.0347, wR_2 = 0.0947	R_1 = 0.0281, wR_2 = 0.0728	R_1 = 0.0344, wR_2 = 0.0942	R_1 = 0.0444, wR_2 = 0.1020	R_1 = 0.0243, wR_2 = 0.0588
$\Delta\rho_{max}$, $\Delta\rho_{min}$ (e Å^{-3})	0.707, -0.654	0.284. -0.433	0.607. -0.435	0.469, -0.397	0.562, -1.474

Complex	1g	1h	2a	2a	2b
**CCDC**	2166988	2166989	2166990	2166991	2166992
**Molecular Formula**	$[C_{24} H_{32} N_4 O_4 Fe]^+[I_3]^-$	$[C_{24} H_{32} N_4 O_4 Fe]^+[Cl]^-$ x $C_2 H_6 O$ x 0.25 $(H_2 O)^b$	$[C_{24} H_{32} N_4 O_4 Fe]^+[N O_3]^-$	$[C_{24} H_{32} N_4 O_4 Fe]^+[N O_3]^-$	$[C_{24} H_{32} N_4 O_4 Fe]^+[B F_4]^-$ x $H_2 O$
M_r (g mol^{-1})	877.08	582.41	558.39	558.39	601.21
T (K)	100(2)	100(2)	100(2)	293(2)	100(2)
**Crystal System**	Monoclinic	Monoclinic	Orthorhombic	Orthorhombic	Monoclinic
**Space Group**	$P2_1/c$ (#14)	$P2_1/c$ (#14)	Pccn (#56)	Pccn (#56)	$P2_1/c$ (#14)
a (Å)	18.8904(5)	7.3320(1)	8.2203(2)	7.5246(1)	7.6802(7)
b (Å)	8.0120(1)	16.5758(2)	17.9327(3)	18.3978(2)	20.4717(19)
c (Å)	20.1289(4)	22.3775(3)	17.1866(3)	19.1075(3)	16.5641(15)
α (°)	90	90	90	90	90
β (°)	108.588(3)	90.404(2)	90	90	99.897(2)
γ (°)	90	90	90	90	90
V (Å^3)	2887.59(11)	2719.55(6)	2533.51(9)	2645.17(6)	2565.6(4)
Z, Z'	4, 1	4, 1	4, 0.5	4, 0.5	4, 1
**Radiation Type**	Mo *Ka*	Cu *Ka*	Cu $K\alpha$	Cu $K\alpha$	Mo *Ka*
μ (mm^{-1})	3.766	5.720	5.237	5.016	0.661
**Crystal Size**	0.143 × 0.060 × 0.042	0.321 × 0.048 × 0.036	0.272 × 0.050 × 0.042	0.158 × 0.103 × 0.064	1.00 × 0.60 × 0.50
**Reflections Measured, Independent Reflections**	57637, 5893	27639, 5665	24801, 2660	12686, 2772	29091, 7471
R_{int}	0.0779	0.0428	0.0980	0.0295	0.0399
**Data / Restraints / Parameters**	5893 / 0 / 327	5665 / 0 / 309	2660 / 0 / 170	2772 / 0 / 171	7471 / 0 / 488
**GooF on F²**	1.093	1.049	1.070	1.038	1.035
**Final *R* Indices [*I>2s(I)]***	R_1 = 0.0391, wR_2 = 0.0996	R_1 = 0.0325, wR_2 = 0.0849	R_1 = 0.0636, wR_2 = 0.1564	R_1 = 0.0316, wR_2 = 0.0877	R_1 = 0.0358, wR_2 = 0.0853
$\Delta\rho_{max}$, $\Delta\rho_{min}$ (e Å^{-3})	1.981, -1.475	0.479, -0.583	1.368, -1.265	0.265, -0.223	0.488, -0.471

Complex	2c	3a	3a	3b	4a
**CCDC**	2166993	2166994	2166995	2166996	2166997
**Molecular Formula**	$[C_{24}H_{32}N_4O_4Fe]^+[F_6P]^-$ x H_2O	$[C_{24}H_{32}N_4O_4Fe]^+[NO_3]^-$	$[C_{24}H_{32}N_4O_4Fe]^+[NO_3]^-$	$[C_{24}H_{32}N_4O_4Fe]^+[BF_4]^-$	$[C_{26}H_{36}N_4O_6Fe]^+[NO_3]^-$ x CH_4O^b
M_r (g mol^{-1})	659.37	558.39	558.39	583.20	650.49
T (K)	293(2)	100(2)	293(2)	100(2)	100(2) K
**Crystal System**	Monoclinic	Monoclinic	Monoclinic	Monoclinic	Monoclinic
**Space Group**	$P2_1/c$ (#14)	P2/c (#13)	P2/c (#13)	P2/c (#13)	$P2_1/c$ (#14)
a (Å)	7.9224(6)	17.2266(3)	17.44550(10)	17.5340(15)	7.3600(2)
b (Å)	20.7034(15)	8.2713(2)	8.33440(10)	8.4068(7)	25.3852(5)
c (Å)	17.0178(12)	17.9701(4)	17.91840(10)	17.3701(15)	15.8505(4)
α (°)	90	90	90	90	90
β (°)	101.514(1)	101.129(2)	100.5440(10)	96.424(2)	102.530(2)
γ (°)	90	90	90	90	90
V (Å^3)	2735.1(3)	2512.34(9)	2561.30(4)	2544.4(4)	2890.90(12)
**Z, Z'**	4, 1	4, 1	4, 1	4, 1	4,
**Radiation Type**	Mo Ka	Mo $K\alpha$	Cu $K\alpha$	Mo Ka	Cu $K\alpha$
μ (mm^{-1})	0.694	0.655	5.180	0.661	4.754
**Crystal Size**	0.60 × 0.50 × 0.40	0.237 × 0.149 × 0.111	0.214 × 0.077 × 0.052	0.80 × 0.20 × 0.20	0.090 × 0.060 × 0.040
**Reflections Measured, Independent Reflections**	44903, 5975	36039, 8644	51335, 5380	16401, 6169	32913, 6025
R_{int}	0.0234	0.0344	0.0196	0.0271	0.0734
**Data / Restraints / Parameters**	5975 / 1 / 543	8644 / 0 / 337	5380 / 0 / 337	6169 / 0 / 472	6025 / 0 / 374
**GooF on F²**	1.059	1.072	1.057	1.057	1.045
**Final R Indices [I>2s(I)]**	R_1 = 0.0336, wR_2 = 0.0868	R_1 = 0.0372, wR_2 = 0.0821	R_1 = 0.0286, wR_2 = 0.0827	R_1 = 0.0422, wR_2 = 0.1005	R_1 = 0.0515, wR_2 = 0.1181
$\Delta\rho_{max}$, $\Delta\rho_{min}$ (e Å^{-3})	0.397, -0.171	0.524, -0.495	0.29, -0.31	0.728, -0.319	0.658, -0.583

Complex	4a	4b	4b	4c	5a
**CCDC**	2166998	2167009	2167010	2167011	2167012
**Molecular Formula**	$[C_{26} H_{36} N_4 O_6 Fe]^+[N O_3]^-$ x C $H_4 O^b$	$[C_{26} H_{36} N_4 O_{6.} Fe]^+[B F_4]^-$ x 0.5 (C H_4 O)^b	$[C_{26} H_{36} N_4 O_{6.} Fe]^+[B F_4]^-$ x 0.5 (C H_4 O)^b	$[C_{26} H_{36} N_4 O_6 Fe]^+[O_4 Cl]^-$ x 0.5 (C H_4 O)^b	$[C_{26} H_{36} N_4 O_4 Fe]^+[F_6 P]^-$ x $C_2 H_6$ O
M_r (g mol^{-1})	638.47	659.27	659.27	671.91	715.47
T (K)	298(2) K	100(2) K	100(2) K	100(2) K	100(2)
**Crystal System**	Monoclinic	Monoclinic	Monoclinic	Orthorhombic	Monoclinic
**Space Group**	$P2_1/c$ (#14)	$P2_1/n$ (#14)	$P2_1/n$ (#14)	$P2_1/n$ (#14)	$P2_1/n$ (#14)
a (Å)	7.4389(3)	7.3608(1)	7.4703(2)	7.3704(2)	20.0400(2)
b (Å)	25.334(1)	25.5467(5)	25.783(1)	25.957(1)	7.70456(7)
c (Å)	16.0847(9)	15.9111(4)	16.1483(5)	15.8498(7)	20.7074(2)
α (°)	90	90	90	90	90
β (°)	102.361(6)	103.313(2)	103.353(3)	102.906(4)	96.5038(8)
γ (°)	90	90	90	90	90
V (Å^3)	2961.0(2))	2911.58(10)	3026.19(17)	2955.7(2)	3176.63(5)
Z, Z'	4,	4, 1	4, 1	4, 1	4, 1
**Radiation Type**	Cu $K\alpha$	Cu Ka	Cu Ka	Cu $K\alpha$	Cu Ka
μ (mm^{-1})	4.621	4.823	4.641	5.485	4.990
**Crystal Size**	0.090 × 0.060 × 0.040	0.180 × 0.040 × 0.020	0.180 × 0.040 × 0.020	0.150 × 0.050 × 0.020	0.256 × 0.204 × 0.185
**Reflections Measured, Independent Reflections**	14246, 4533	23209, 6013	28967, 5205	20771, 6204	37576, 6685
R_{int}	0.0493	0.0462	0.0490	0.0735	0.0295
**Data / Restraints / Parameters**	4533 / 0 / 374	6013 / 0 / 397	5205 / 28 / 407	6204 / 0 / 382	6685 / 0 / 407
**GooF on F²**	1.020	1.058	1.039	1.052	1.082
**Final R Indices [$I>2s(I)$]**	R_1 = 0.0557, wR_2 = 0.1370	R_1 = 0.0479, wR_2 = 0.1287	R_1 = 0.0596, wR_2 = 0.1659	R_1 = 0.0758, wR_2 = 0.2043	R_1 = 0.0720, wR_2 = 0.1962
$\Delta\rho_{max}, \Delta\rho_{min}$ (e Å^{-3})	0.521, -0.372	0.729, -0.846	0.435, -0.550	0.871, -0.643	1.623, -0.850

Complex	5b	6b	6b·S	6c	6c
**CCDC**	2167013	2044253	2167014	2044252	2167015
**Molecular Formula**	$[C_{26}H_{36}N_4O_4Fe]^+[BF_4]^-$ x 0.315 (H_2O)	$[C_{30}H_{46}N_6O_2Fe]^+[F_6P]^-$	$[C_{30}H_{46}N_6O_2Fe]^+[F_6P]^-$ x 0.78(C_2H_3N) x 0.1(C_2H_6O)^b	$[C_{30}H_{46}N_6O_2Fe]^+[CO_3F_3S]^-$	$[C_{30}H_{46}N_6O_2Fe]^+[CO_3F_3S]^-$
M_r (g mol^{-1})	616.92	723.55	759.94	727.65	727.65
T (K)	100(2)	100(2)	100(2)	100(2)	293(2)
**Crystal System**	Monoclinic	Triclinic	Triclinic	Triclinic	Triclinic
**Space Group**	Pn (#7)	P-1 (#2)	P-1 (#2)	P-1 (#2)	P-1 (#2)
a (Å)	7.46133(5)	7.80570(7)	13.0164(2)	7.58348(6)	7.71714(7)
b (Å)	11.00091(7)	13.8253(2)	16.1629(2)	14.6609(2)	14.0529(1)
c (Å)	17.2247(1)	16.6288(2)	17.6941(2)	16.2660(2)	17.4409(1)
α (°)	90	68.5850(9)	78.1099(7)	108.0496(9)	71.2422(7)
β (°)	102.4789(6)	85.2660(7)	80.0716(8)	99.1600(7)	82.0484(7)
γ (°)	90	88.3907(7)	89.3125(7)	91.9331(7)	89.7708(7)
V (Å^3)	1380.427(5)	1664.92(4)	3587.04(8)	1690.88(3)	1775.88(2)
Z, Z'	2, 1	2, 1	2, 2	2, 1	2, 1
**Radiation Type**	Cu *Ka*	Mo *Ka*	Cu *Ka*	Cu *Ka*	Cu *Ka*
μ (mm^{-1})	4.984	0.572	4.412	4.712	4.487
**Crystal Size**	0.216 × 0.157 × 0.073	0.327 × 0.220 × 0.151	0.209 × 0.110 × 0.028	0.344 × 0.093 × 0.069	0.328 × 0.103 × 0.066
**Reflections Measured, Independent Reflections**	27616, 5557	106699, 11880	91164, 15049	53375, 7090	69770, 7455
R_{int}	0.0191	0.0338	0.0485	0.0370	0.0418
**Data / Restraints / Parameters**	5557 / 2 / 376	11880 / 0 / 419	15049 / 0 / 907	7090 / 0 / 428	7455 / 0 / 428
**GooF on F²**	1.048	1.071	1.067	1.080	1.102
**Final R Indices [$I>2s(I)$]**	$R_1 = 0.0208$, $wR_2 = 0.0548$	$R_1 = 0.0316$, $wR_2 = 0.0767$	$R_1 = 0.0466$, $wR_2 = 0.1277$	$R_1 = 0.0273$, $wR_2 = 0.0735$	$R_1 = 0.0391$, $wR_2 = 0.1132$
$\Delta\rho_{max}$, $\Delta\rho_{min}$ (e Å^{-3})	0.166, -0.253	0.525, -0.485	0.975, -0.651	0.423, -0.391	0.444, -0.417
**Flack Parameter**	-0.0095(11)	---	---	---	---

Complex	6d	6d·S	6e	7a	7b
**CCDC**	2044257	2167016	2167017	2167018	2167019
**Molecular Formula**	$[C_{30}\ H_{46}\ N_6\ O_2\ Fe]^+[B\ F_4]^-$	$[C_{30}\ H_{46}\ N_6\ O_2\ Fe]^+[B\ F_4]^-$ x $C_2\ H_6\ O$[b]	$[C_{30}\ H_{46}\ N_6\ O_2\ Fe]^+[N\ O_3]^-$ x $C\ H_2\ Cl_2$	$[C_{24}\ H_{32}\ N_4\ O_2\ Fe]^+[O_4\ Cl]^-$	$[C_{24}\ H_{32}\ N_4\ O_2\ Fe]^+[F_6\ P]^-$ x 0.68 $(H_2\ O)$[c]
$\boldsymbol{M_r}$ (g mol^{-1})	665.39	711.45	725.51	563.83	621.69
$\boldsymbol{T}$ (K)	100(2)	100(2)	100(2)	100(2)	100(2)
**Crystal System**	Monoclinic	Orthorhombic	Orthorhombic	Orthorhombic	Monoclinic
**Space Group**	$P2_1/n$ (#14)	$P2_12_12_1$ (#19)	$P2_12_12_1$ (#19)	$P2_12_12_1$ (#19)	C2/c (#14)
$\boldsymbol{a}$ (Å)	7.6682(1)	7.6914(2)	7.65722(8)	8.42810(9)	13.3659(2)
$\boldsymbol{b}$ (Å)	19.6632(4)	19.7983(4)	19.4470(2)	16.5915(2)	11.2845(2)
$\boldsymbol{c}$ (Å)	21.3813(4)	23.0219(4)	23.1185(3)	17.8926(2)	17.3363(2)
$\boldsymbol{\alpha}$ (°)	90	90	90	90	90
$\boldsymbol{\beta}$ (°)	91.087(2)	90	90	90	100.708(1)
$\boldsymbol{\gamma}$ (°)	90	90	90	90	90
$\boldsymbol{V}$ (Å^3)	3223.32(10)	3505.70(13)	3442.57(7)	2502.01(5)	2569.26(7)
Z, Z'	4, 1	4, 1	4, 1	4	4, 0.5
**Radiation Type**	Cu *Ka*	Cu *Ka*	Cu *Ka*	Cu *Ka*	Cu $K\alpha$
$\boldsymbol{\mu}$ (mm^{-1})	4.2470	3.981	5.348	6.225	6.003
**Crystal Size**	0.248 × 0.073 × 0.050	0.178 × 0.039 × 0.023	0.305 × 0.047 × 0.038	0.144 × 0.037 × 0.023	0.204 × 0.188 × 0.084
**Reflections Measured, Independent Reflections**	35206, 6776	17939, 7309	18331, 7175	26098, 5239	12503, 2674
$\boldsymbol{R_{int}}$	0.0627	0.0616	0.0346	0.0705	0.0191
**Data / Restraints / Parameters**	6776 / 0 / 401	7309 / 0 / 401	7175 / 0 / 425	5239 / 0 / 327	2674 / 0 / 192
**GooF on F²**	1.040	0.967	1.051	1.019	1.124
**Final *R* Indices [*I*>*2s(I)*]**	$R_1 = 0.0487$, $wR_2 = 0.1254$	$R_1 = 0.0381$, $wR_2 = 0.0743$	$R_1 = 0.0608$, $wR_2 = 0.1709$	$R_1 = 0.0339$, $wR_2 = 0.0778$	$R_1 = 0.0259$, $wR_2 = 0.0718$
$\boldsymbol{\Delta\rho_{max}, \Delta\rho_{min}}$ (e Å^{-3})	1.077, -0.659	0.246, -0.272	1.016, -1.380	0.264, -0.289	0.351, -0.448
**Flack Parameter**	---	-0.006(3)	0.212(7)[a]	-0.023(3)	---

Complex	7c	8	9a	9b	10e
**CCDC**	2167020	2167021	2167022	2167023	2167024
**Molecular Formula**	$[C_{24}H_{32}N_4O_2Fe]^+[BF_4]^-$	$[C_{30}H_{39}N_6O_5Fe]^+[NO_3]^-$ x C_2H_3N[b]	$([C_{30}H_{44}N_4O_2Fe]^+[F_6P]^-)_2$ x C_2H_6O	$[C_{30}H_{44}N_4O_2Fe]^+[BF_4]^-$	$[C_{22}H_{28}N_4O_2Fe]^+[Cl_4Fe]^-$
$\boldsymbol{M_r}$ (g mol^{-1})	551.19	619.52	1433.09	635.35	633.98
$\boldsymbol{T}$ (K)	100(2)	100(2)	100(2)	293(2)	100(2)
**Crystal System**	Orthorhombic	Monoclinic	Monoclinic	Tetragonal	Orthorhombic
**Space Group**	$P2_12_12_1$ (#19)	$P2_1/c$ (#14)	$P2_1/c$ (#14)	$P4_322$ (#95)	$P2_12_12_1$ (#19)
$\boldsymbol{a}$ (Å)	8.2990(1)	7.90357(9)	11.1204(9)	8.7437(16)	7.7052(1)
$\boldsymbol{b}$ (Å)	16.6365(2)	17.0622(2)	34.653(3)	8.7437(16)	17.4070(3)
$\boldsymbol{c}$ (Å)	17.9173(2)	22.6549(3)	17.6469(15)	42.142(15)	19.7425(3)
$\boldsymbol{\alpha}$ (°)	90	90	90	90	90
$\boldsymbol{\beta}$ (°)	90	96.780(1)	103.925(2)	90	90
$\boldsymbol{\gamma}$ (°)	90	90	90	90	90
$\boldsymbol{V}$ (Å^3)	2473.78(5)	3033.70(6)	6600.4(10)	3221.8(16)	2647.95(7)
**Z, Z'**	4, 1	4, 1	4, 2	4, 0.5	4, 1
**Radiation Type**	Cu $K\alpha$	Mo *Ka*	Mo *Ka*	Mo *Ka*	Mo *Ka*
$\boldsymbol{\mu}$ (mm^{-1})	5.419	0.546	0.576	0.524	1.527
**Crystal Size**	0.515 × 0.046 × 0.039	0.284 × 0.191 × 0.069	0.80 × 0.30 × 0.20	0.50 × 0.50 × 0.40	0.285 × 0.111 × 0.075
**Reflections Measured, Independent Reflections**	25661, 5188	52588, 7801	10341, 10341	17421, 3800	18710, 6338
$\boldsymbol{R_{int}}$	0.0578	0.0259	0.0481	0.0625	0.0260
**Data / Restraints / Parameters**	5188 / 0 / 335	7801 / 0 / 390	10341 / 0 / 855	3800 / 0 / 195	6338 / 0 / 312
**GooF on F²**	1.018	1.051	1.091	1.166	1.091
**Final *R* Indices [*I>2s(I)*]**	$R_1 = 0.0315$, $wR_2 = 0.0775$	$R_1 = 0.0458$, $wR_2 = 0.1291$	$R_1 = 0.0487$, $wR_2 = 0.1094$	$R_1 = 0.0839$, $wR_2 = 0.1875$	$R_1 = 0.0295$, $wR_2 = 0.0645$
$\boldsymbol{\Delta\rho_{max}, \Delta\rho_{min}}$ (e Å^{-3})	0.267, -0.475	0.496, -0.703	0.606, -0.499	0.67, -1.08	0.648, -0.458
**Flack Parameter**	-0.017(2)	---	---	0.44(6)[a]	-0.017(12)

Complex	10f	11a	11b	11c	12
**CCDC**	2167025	2167026	2167027	2167028	2166999
**Molecular Formula**	$[C_{22} H_{28} N_4 O_2 Fe]^+[B F_4]^-$	$[C_{22} H_{26} N_4 O_2 Fe Br_2]^+[F_6 P]^-$	$[C_{22} H_{26} N_4 O_2 Fe Br_2]^+[B F_4]^-$ x $C_2 H_6 O$	$[C_{22} H_{26} N_4 O_2 Fe Br_2]^+[N O_3]^-$ x $C_3 H_8 O$	$[C_{22} H_{24} N_4 O_2 Fe Br_4]^+[N O_3^-]$ x $C_3 H_8 O$
M_r (g mol^{-1})	523.14	739.11	727.02	716.24	874.05
T (K)	100(2)	293(2)	293(2)	100(2)	100(2)
**Crystal System**	Monoclinic	Monoclinic	Triclinic	Monoclinic	Monoclinic
**Space Group**	$P2_1/c$ (#14)	$P2_1$ (#4)	P-1 (#2)	$P2_1/n$ (#14)	$P2_1/n$ (#14)
a (Å)	7.4795(2)	7.889(4)	8.5033(6)	18.7006(18)	10.6549(13)
b (Å)	15.2664(4)	17.883(9)	9.8037(7)	8.0056(8)	14.3738(17)
c (Å)	20.2861(6)	9.483(5)	17.5760(13)	38.606(4)	20.851(3)
α (°)	90	90	84.3930(10)	90	90
β (°)	93.155(3)	92.588(8)	81.8850(10)	96.466(2)	103.324(2)
γ (°)	90	90	81.8740(10)	90	90
V (Å^3)	2312.86(11)	1336.6(12)	1431.44(18)	5742.9(10)	3107.4(6)
Z, Z'	4	2, 1	2, 1	4, 2	4, 1
**Radiation Type**	Mo *Ka*	Mo *Ka*	Mo *Ka*	Mo *Ka*	Mo *Ka*
μ (mm^{-1})	0.712	3.684	3.378	3.356	5.676
**Crystal Size**	0.291 × 0.188 × 0.177	0.50 × 0.30 × 0.20	0.50 × 0.30 × 0.10	0.80 × 0.80 × 0.30	0.60 × 0.50 × 0.40
**Reflections Measured, Independent Reflections**	25697, 5944	22230, 6194	30801, 7599	104961, 13870	27008, 6785
R_{int}	0.0323	0.0222	0.0244	0.0599	0.0742
**Data / Restraints / Parameters**	5944 / 0 / 324	6194 / 1 / 344	7599 / 0 / 362	13870 / 0 / 731	6785 / 0 / 391
**GooF on F²**	1.054	1.040	1.032	1.103	1.029
**Final *R* Indices [*I>2s(I)*]**	R_1 = 0.0330, wR_2 = 0.0767	R_1 = 0.0329, wR_2 = 0.0854	R_1 = 0.0387, wR_2 = 0.1041	R_1 = 0.0409, wR_2 = 0.0960	R_1 = 0.0447, wR_2 = 0.1025
$\Delta\rho_{max}$, $\Delta\rho_{min}$ (e Å^{-3})	0.387, -0.496	0.820, -0.302	0.941, -0.639	1.180, -0.899	1.270, -1.027
**Flack Parameter**	---	0.497(6)[a]	---	---	---

Complex	13a	13b	14	15a	15b
**CCDC**	2167000	2167001	2167002	2167003	2167004
**Molecular Formula**	$[C_{22} H_{24} N_4 O_2 Cl_4 Fe]^+[B F_4]^-$ x $C_3 H_8 O$	$[C_{22} H_{24} N_4 O_2 Cl_4 Fe]^+[F_6 P]^-$	$[C_{22} H_{24} N_4 O_2 Fe I_4]^+[F_6 P]^-$	$[C_{22} H_{26} N_6 O_6 Fe]^+[F_6 P]^-$ x $C_2 H_3 N$	$[C_{22} H_{26} N_6 O_6 Fe]^+[N O_3]^-$
M_r (g mol^{-1})	721.01	719.07	1084.87	712.36	588.35
T (K)	293(2)	100(2)	100(2)	293(2)	100(2)
**Crystal System**	Monoclinic	Monoclinic	Monoclinic	Monoclinic	Monoclinic
**Space Group**	P2/n (#13)	$P2_1/n$ (#14)	$P2_1/c$ (#14)	$P2_1/n$ (#14)	Cc (#9)
a (Å)	17.633(4)	10.8760(8)	9.0211(7)	8.8737(7)	10.3078(14)
b (Å)	8.4837(17)	18.6961(13)	17.5555(14)	13.9089(11)	12.9869(17)
c (Å)	21.240(4)	13.6869(9)	18.4041(15)	24.3218(19)	17.856(2)
α (°)	90	90	90	90	90
β (°)	96.596(4)	95.7120(10)	96.692(2)	93.6640(10)	99.035(2)
γ (°)	90	90	90	90	90
V (Å^3)	3156.2(11)	2769.3(3)	2894.8(4)	2995.7(4)	2360.7(5)
Z, Z'	4, 1	4, 1	4, 1	4, 1	4, 1
**Radiation Type**	Mo *Ka*	Mo *Ka*	Mo *Ka*	Mo *Ka*	Mo *Ka*
μ (mm^{-1})	0.874	1.059	4.911	0.646	0.710
**Crystal Size**	1.00 × 0.50 × 0.30	0.60 × 0.50 × 0.02	0.30 × 0.10 × 0.03	0.60 × 0.30 × 0.03	0.50 × 0.40 × 0.02
**Reflections Measured, Independent Reflections**	56846, 6890	62599, 8081	64939, 8443	49140, 6531	10803, 5513
R_{int}	0.0204	0.0325	0.0335	0.0238	0.0324
**Data / Restraints / Parameters**	6890 / 0 / 479	8081 / 0 / 457	8443 / 0 / 361	6531 / 0 / 440	5513 / 2 / 352
**GooF on F²**	1.034	1.058	1.114	1.036	1.016
**Final *R* Indices [*I>2s(I)]***	$R_1 = 0.0402$, $wR_2 = 0.1055$	$R_1 = 0.0413$, $wR_2 = 0.1008$	$R_1 = 0.0332$, $wR_2 = 0.0803$	$R_1 = 0.0465$, $wR_2 = 0.1237$	$R_1 = 0.0404$, $wR_2 = 0.0836$
$\Delta\rho_{max}$, $\Delta\rho_{min}$ (e Å^{-3})	0.472, -0.367	1.421, -0.647	2.429, -0.855	0.547, -0.398	0.749, -0.324
**Flack Parameter**	---	---	---	---	0.015(14)

Complex	16a	16b	16c	17
CCDC	2167005	2167006	2167007	2167008
Molecular Formula	$[C_{22} H_{26} N_6 O_6 Fe]^+[F_6 P]^-$ x $C_2 H_6 O$	$[C_{22} H_{26} N_6 O_6 Fe]^+[B F_4]^-$ x $C_2 H_6 O$	$[C_{22} H_{26} N_6 O_6 Fe]^+[O_4 Cl]^-$ x $C_2 H_6 O$	$[C_{24} H_{30} N_8 O_{10} Fe]^+[O_4 Cl]^-$ x $C_2 H_6 O$
$\boldsymbol{M_r}$ (g mol^{-1})	717.38	659.22	671.85	761.86
$\boldsymbol{T}$ (K)	293(2)	293(2)	100(2)	100(2)
**Crystal System**	Triclinic	Triclinic	Triclinic	Monoclinic
**Space Group**	P-1 (#2)	P-1 (#2)	P-1 (#2)	$P2_1/n$ (#14)
$\boldsymbol{a}$ (Å)	8.5699(11)	8.4309(18)	8.3554(3)	9.49602(8)
$\boldsymbol{b}$ (Å)	9.5942(12)	9.630(2)	9.4905(4)	18.2529(2)
$\boldsymbol{c}$ (Å)	18.508(2)	18.161(4)	18.1299(7)	17.1490(2)
$\boldsymbol{\alpha}$ (°)	84.697(2)	84.807(4)	85.469(3)	90
$\boldsymbol{\beta}$ (°)	90.925(2)	80.395(4)	78.615(4)	91.8604(7)
$\boldsymbol{\gamma}$ (°)	83.704(2)	82.295(3)	82.580(4)	90
$\boldsymbol{V}$ (Å^3)	1489.2(3)	1437.2(5)	1395.44(10)	2970.87(5)
**Z, Z'**	2, 1	2, 1	2, 1	4, 1
**Radiation Type**	Mo *Ka*	Mo *Ka*	Cu *Ka*	Cu *Ka*
$\boldsymbol{\mu}$ (mm^{-1})	0.651	0.604	5.848	5.704
**Crystal Size**	1.00 × 0.80 × 0.20	0.40 × 0.40 × 0.02	0.140 × 0.049 × 0.019	0.236 × 0.122 × 0.046
**Reflections Measured, Independent Reflections**	23123, 5835	9118, 4438	16828, 5792	31000, 6222
$\boldsymbol{R_{int}}$	0.0258	0.0207	0.0614	0.0333
**Data / Restraints / Parameters**	5835 / 0 / 549	4438 / 0 / 390	5792 / 0 / 390	6222 / 0 / 455
**GooF on F^2**	1.026	1.043	1.012	1.041
**Final *R* Indices [*I>2s(I)*]**	$R_1 = 0.0389$, $wR_2 = 0.1028$	$R_1 = 0.0578$, $wR_2 = 0.1597$	$R_1 = 0.0411$, $wR_2 = 0.0936$	$R_1 = 0.0270$, $wR_2 = 0.0685$
$\boldsymbol{\Delta\rho_{max}, \Delta\rho_{min}}$ (e Å^{-3})	0.360, -0.297	1.082, -0.579	0.358, -0.431	0.284, -0.441

[a]The structure was refined as a two-component inversion twin. [b]The solvent could not be modelled in terms of atomic sites. PLATON SQUEEZE was used to compensate for the spread electron density. [c]The hydrogen atoms of the water molecules could not be detected.

S2.2 Bond Lengths and Distortion Parameters

Table S2.2. Summary of bond lengths and distortion parameters for **1** – **17**.

Complex	1a		1a·S		1b	1c	1d		1e	1f	1g	1h
**Temperature (K)**	**100**	**293**	**100**	**293**	**100**	**100**	**100**	**200**	**293**	**100**	**100**	**100**
**Comment**												
**Bond Lengths (Å)**												
**Fe–O$_{phen}$**	1.908(2)	1.923(3)	1.8785(11)	1.8768(10)	1.878(1)	1.879(1)	1.881(1)	1.8748(12)	1.875(2)	1.874(1)	1.877(3)	1.8781(11)
			1.8858(11)	1.8841(10)	1.878(1)	1.887(1)	1.885(1)	1.8776(12)	1.877(2)	1.879(1)	1.874(3)	1.8935(11)
**Fe–N$_{iminie}$**	1.982(2)	2.048(4)	1.9514(12)	1.9487(12)	1.958(2)	1.961(2)	1.956(1)	1.9568(16)	1.950(3)	1.947(2)	1.945(4)	1.9567(14)
			1.9545(13)	1.9528(13)	1.949(1)	1.947(1)	1.957(1)	1.9568(14)	1.957(3)	1.955(2)	1.946(4)	1.9614(14)
**Fe–N$_{amine}$**	2.051(3)	2.102(4)	2.0154(12)	2.0166(12)	2.020(1)	2.025(1)	2.023(1)	2.0221(15)	2.017(3)	2.022(2)	2.023(4)	2.0142(13)
			2.0171(13)	2.0175(13)	2.023(2)	2.027(1)	2.027(1)	2.0251(15)	2.023(3)	2.025(2)	2.017(4)	2.0196(14)
**Distortion Parameters (°)**												
**Σ**	34.26	52.06	24.13	25.22	27.78	25.63	21.83	27.34	26.62	27.79	25.33	29.23
**Θ**	127.29	200.78	58.94	63.53	71.98	68.02	59.15	73.46	72.86	71.75	58.11	77.52
**α**	49.39	50.93	44.29	45.89	45.55	39.50	40.91	41.24	40.89	41.92	43.77	40.73
**τ**	30.80	30.02	26.04<br>26.84	25.17<br>26.10	31.78<br>26.88	32.27<br>26.13	29.08<br>27.39	29.35<br>29.28	29.19<br>30.21	30.74<br>26.13	25.25<br>25.44	28.61<br>29.24

**Complex**	**2a**		**2b**	**2c**	**2d**	**2e**	**3a**				**3b**	
**Temperature (K)**	**100**	**293**	**100**	**293**	**293**	**100**	**100**		**293**		**100**	
**Comment**							**Site I**	**Site II**	**Site I**	**Site II**	**Site I**	**Site II**
**Bond Lengths (Å)**												
**Fe–O$_{phen}$**	1.870(1)	1.8778(10)	1.855(1)	1.877(1)	1.861(2)	1.893(1)	1.876(1)	1.874(1)	1.8767(10)	1.8798(10)	1.873(1)	1.875(1)
			1.871(1)	1.879(1)	1.867(2)	1.882(1)						
**Fe–N$_{iminie}$**	1.959(2)	1.9578(14)	1.953(1)	1.954(1)	1.944(2)	1.945(1)	1.943(1)	1.944(1)	1.9420(12)	1.9514(11)	1.951(2)	1.959(2)
			1.967(1)	1.966(1)	1.946(2)	1.944(1)						
**Fe–N$_{amine}$**	2.017(2)	2.0170(14)	2.024(1)	2.019(1)	2.012(2)	2.025(1)	2.014(1)	2.012(1)	2.0188(13)	2.0180(12)	2.010(2)	2.014(2)
			2.022(1)	2.022(2)	2.016(2)	2.023(1)						
**Distortion Parameters (°)**												
**Σ**	35.59	29.20	28.65	28.27	30.71	29.90	21.94	24.24	29.92	23.86	28.51	26.47
**Θ**	99.98	80.03	80.74	78.45	79.62	78.11	50.17	58.49	73.33	61.35	79.56	70.26
**α**	66.64	40.95	40.66	39.22	45.97	36.79	38.15	38.86	37.71	37.49	35.47	32.41
**τ**	28.33	28.88	36.52<br>28.17	35.02<br>27.90	33.43<br>27.30	30.52<br>28.42	26.18	30.18	27.84	29.56	29.98	32.70

**Complex**	**4a**	**4a**	**4b**	**4b**	**4c**	**5a**	**5b**	**6a**	**6b**	**6b·S**		**6c**
**Temperature (K)**	**100**	**293**	**100**	**293**	**100**	**100**	**100**	**100**	**100**	**100**		**100**
**Comment**										**Site I**	**Site II**	
**Bond Lengths (Å)**												
**Fe–O$_{phen}$**	1.8922(19)	1.889(2)	1.881(2)	1.883(3)	1.885(4)	1.862(2)	1.8739(15)	1.884(2)	1.878(1)	1.8802(15)	1.8728(14)	1.878(1)
	1.8939(19)	1.893(2)	1.893(2)	1.885(3)	1.893(4)	1.888(2)	1.8860(15)	1.879(2)	1.874(1)	1.8815(15)	1.8741(14)	1.879(1)
**Fe–N$_{iminie}$**	1.955(2)	1.947(3)	1.950(3)	1.946(4)	1.947(5)	1.952(3)	1.9524(18)	1.952(3)	1.949(1)	1.9438(17)	1.9416(18)	1.954(1)
	1.956(2)	1.948(3)	1.952(3)	1.950(3)	1.952(5)	1.958(3)	1.9652(19)	1.955(3)	1.950(1)	1.9465(18)	1.9465(16)	1.955(1)
**Fe–N$_{amine}$**	2.020(2)	2.016(3)	2.019(3)	2.012(4)	2.012(5)	2.016(3)	2.0258(19)	2.026(3)	2.023(1)	2.026(2)	2.0157(19)	2.024(1)
	2.028(2)	2.017(3)	2.031(3)	2.020(4)	2.029(5)	2.023(3)	2.0288(18)	2.026(3)	2.021(1)	2.0322(18)	2.0212(17)	2.025(1)
**Distortion Parameters (°)**												
**Σ**	29.29	27.79	29.11	27.26	28.51	25.32	24.95	27.87	27.62	27.69	31.36	28.78
**Θ**	83.14	73.82	79.30	70.69	77.47	70.20	76.39	72.14	71.25	67.81	81.93	77.34
**α**	38.96	40.07	40.77	41.63	41.89	45.55	50.55	57.75	51.24	50.24	50.97	56.33
**τ**	31.37<br>29.50	28.44<br>29.82	28.66<br>31.54	27.99<br>29.42	28.75<br>30.05	27.49<br>33.12	29.82<br>33.37	29.38<br>28.95	30.23<br>24.74	21.19<br>32.89	19.43<br>25.43	29.03<br>29.58

Complex	6c	6d	6d·S	6e	7a	7b	7c	8	9a		9b	10a
**Temperature**	**293**	**100**	**100**	**100**	**100**	**100**	**100**	**100**	**100**		**293**	**100**
**Comment**									**Site I**	**Site II**		
**Bond Lengths (Å)**												
**Fe–O$_{phen}$**	1.8768(13)	1.8671(16)	1.884(2)	1.885(3)	1.869(3)	1.878(1)	1.873(2)	1.8845(13)	1.904(2)	1.896(3)	1.919(6)	1.873(3)
	1.8798(12)	1.8740(16)	1.889(2)	1.889(3)	1.871(3)		1.872(2)	1.8859(13)	1.891(2)	1.895(3)	1.908(6)	1.870(3)
**Fe–N$_{iminie}$**	1.9551(16)	1.9463(18)	1.946(3)	1.951(4)	1.946(3)	1.950(1)	1.943(2)	1.9821(15)	1.920(3)	1.921(2)	1.924(8)	1.944(4)
	1.9564(16)	1.9479(18)	1.952(3)	1.967(4)	1.949(3)		1.948(2)	1.9833(15)	1.924(2)	1.917(2)	1.926(7)	1.957(4)
**Fe–N$_{amine}$**	2.0232(16)	2.0231(18)	2.021(3)	2.008(4)	2.013(3)	2.025(1)	2.013(2)	2.0490(14)	2.014(2)	2.017(3)	2.003(7)	2.020(6)
	2.0276(16)	2.0245(18)	2.022(3)	2.026(4)	2.019(3)		2.023(2)	2.0528(16)	2.003(3)	2.011(3)	2.023(7)	2.012(6)
**Distortion Parameters (°)**												
**Σ**	27.53	30.17	26.20	26.07	26.82	24.00	27.18	25.72	21.94	24.24	19.58	28.27
**Θ**	75.70	79.92	68.86	68.45	65.95	61.26	65.94	76.93	50.17	58.49	44.76	80.76
**α**	52.66	54.14	51.90	52.24	40.66	48.97	40.11	49.46	31.74	32.46	32.08	49.39
**τ**	26.43<br>30.61	25.00<br>26.23	26.26<br>28.89	26.00<br>29.62	17.73<br>24.66	27.28	25.49<br>17.67	25.55<br>25.46	22.94<br>13.84	16.47<br>18.83	20.29<br>19.03	28.16<br>27.87

Complex	10b	10c	10d	10e	10f	11a	11b	11c		12	13a	
**Temperature**	**293**	**100**	**100**	**100**	**100**	**293**	**293**	**100**		**100**	**293**	
**Comment**								**Site I**	**Site II**		**Site I**	**Site II**
**Bond Lengths (Å)**												
**Fe–O$_{phen}$**	1.891(8)	1.882(4)	1.874(1)	1.882(2)	1.884(1)	1.882(2)	1.885(2)	1.871(2)	1.873(2)	1.883(3)	1.879(2)	1.876(2)
	1.896(8)		1.879(1)	1.879(2)	1.875(1)	1.893(2)	1.875(2)	1.880(2)	1.888(2)	1.882(3)		
**Fe–N$_{iminie}$**	1.95(1)	1.957(5)	1.961(1)	1.955(2)	1.953(1)	1.961(3)	1.960(2)	1.955(2)	1.955(2)	1.944(4)	1.954(2)	1.954(2)
	1.96(1)		1.953(1)	1.955(2)	1.956(1)	1.954(3)	1.958(2)	1.952(2)	1.954(2)	1.955(3)		
**Fe–N$_{amine}$**	2.01(1)	2.018(5)	2.032(1)	2.018(2)	2.018(1)	2.015(3)	2.011(2)	2.010(2)	2.010(2)	2.011(4)	2.009(2)	2.014(2)
	2.02(1)		2.023(1)	2.022(2)	2.023(1)	2.013(3)	2.002(2)	2.012(2)	2.011(2)	2.004(4)		
**Distortion Parameters (°)**												
**Σ**	28.43	30.10	27.29	26.02	25.30	25.94	22.38	25.95	25.92	24.74	23.63	29.90
**Θ**	74.16	88.40	69.38	68.49	63.64	65.46	59.20	71.41	68.38	63.01	60.99	78.11
**α**	49.39	50.49	47.79	44.43	48.73	45.37	32.01	43.42	35.29	41.85	36.34	36.79
**τ**	26.38<br>29.15	29.50	25.80<br>26.88	30.81<br>28.18	26.89<br>26.73	28.44<br>27.41	28.50<br>27.85	28.61<br>28.86	27.38<br>27.12	24.87<br>22.65	28.50	28.84

Complex	13b	14	15a	15b	16a	16b	16c	17				
**Temperature (K)**	**100**	**100**	**293**	**100**	**293**	**293**	**100**	**100**				
**Comment**												
**Bond Lengths (Å)**												
**Fe–O$_{phen}$**	1.874(1)	1.879(2)	1.880(1)	1.887(2)	1.881(2)	1.886(3)	1.887(2)	1.889(1)				
	1.863(1)	1.874(2)	1.884(1)	1.890(2)	1.890(2)	1.900(3)	1.896(2)	1.891(1)				
**Fe–N$_{iminie}$**	1.946(2)	1.950(3)	1.941(2)	1.950(3)	1.955(2)	1.959(3)	1.957(2)	1.951(1)				
	1.949(2)	1.945(3)	1.933(2)	1.957(2)	1.959(2)	1.956(2)	1.958(2)	1.955(1)				
**Fe–N$_{amine}$**	2.021(2)	2.011(3)	2.005(2)	2.008(2)	2.001(2)	1.999(3)	2.006(2)	2.008(1)				
	2.016(2)	2.007(3)	2.006(2)	2.004(3)	2.007(2)	2.017(3)	2.009(2)	2.003(1)				
**Distortion Parameters (°)**												
**Σ**	24.54	25.70	27.96	29.94	24.05	22.36	21.02	27.75				
**Θ**	60.26	64.96	65.59	72.17	63.44	58.70	56.65	76.98				
**α**	45.13	20.18	34.51	44.74	26.07	26.67	26.66	21.29				
**τ**	27.62<br>22.40	26.12<br>25.80	19.42<br>26.06	29.06<br>17.47	26.34<br>29.30	26.24<br>28.88	30.54<br>25.58	27.21<br>24.98				

S2.3 Intermolecular Interactions

Table S2.3. Intermolecular hydrogen bonds for all complexes. Symmetry operations used to generate equivalent atoms are shown beneath each set of hydrogen bonds.

D–H...A	d(D–H)	d(H...A)	d(D...A)	<(DHA)
**1b** [Fe^{III}(4-OMe-sal$_2$323)]PF$_6$·0.45H$_2$O				
O(5)–H(2O5)...O(4)	0.84	2.18	2.961(4)	155.6
O(5)–H(1O5)...F(5B)	0.84	2.17	2.912(10)	147.5
O(5)–H(1O5)...F(4B)	0.84	2.22	2.953(6)	145.3
N(2)–H(2)...O(5)#1	1.00	2.35	3.315(5)	160.7
N(2)–H(2)...F(4A)#1	1.00	2.40	3.152(4)	131.6
#1 x–1,y,z				
**1c** [Fe^{III}(4-OMe-sal$_2$323)]OTf·0.27H$_2$O				
N(2)–H(1N2)...O(6A)#1	0.87(2)	2.44(2)	3.048(3)	127.3(19)
N(2)–H(1N2)...O(6B)#1	0.87(2)	2.44(2)	3.141(7)	138(2)
N(3)–H(1N3)...O(9)#2	0.91(2)	2.31(2)	3.202(6)	167.3(19)
#1 x–1,–y+3/2,z–1/2; #2 x,–y+3/2,z–1/2				
**1d** [Fe^{III}(4-OMe-sal$_2$323)]ClO$_4$				
N(3)–H(3)...O(5)#1	1.00	2.22	3.0784(17)	143.1
100 K – #1 x,y,z+1				
N(3)–H(3)...O(5)#1	1.00	2.26	3.075(2)	138.0
N(2)–H(1N2)...O(5)#2	1.00	2.21	3.063(2)	142.1
200 K – #1 x,y,z+1; #2 x–1,y,z+1				
**1e** [Fe^{III}(4-OMe-sal$_2$323)]BF$_4$				
N(3)–H(1N3)...F(1)#1	0.91	2.29	3.072(4)	144.4
N(2)–H(1N2)...F(1)#2	0.91	2.30	3.055(4)	140.7
#1 –x,1–y,–z; #2 1–x,1–y,2–z				
**1f** [Fe^{III}(4-OMe-sal$_2$323)]SbF$_6$·0.31H$_2$O				
N(3)–H(1N3)...F(5A)#1	1.00	2.35	3.132(5)	134.9
N(3)–H(1N3)...O(5)#2	1.00	2.44	3.392(6)	158.4
N(2)–H(1N2)...O(5)	1.00	2.50	3.478(6)	166.9
#1 –x,1/2+y,1/2–z; #2 –1+x,y,z				
**1g 1373** [Fe^{III}(4-OMe-sal$_2$323)]I$_3$				
N(2)–H(2)...I(2)#1	1.00	2.98	3.861(4)	147.3
N(3)–H(3)...I(2)#2	1.00	2.93	3.733(4)	137.6
#1 –x+1,y+1/2,–z+1/2; #2 –x+1,y–1/2,–z+1/2				
**1h** [Fe^{III}(4-OMe-sal$_2$323)]Cl·EtOH·0.25H$_2$O				
N(3)–H(1N3)...Cl#1	1.00	2.54	3.2530(14)	127.8
N(2)–H(1N2)...Cl	1.00	2.39	3.1770(14)	134.8
#1 x+1,y,z				

D–H...A	d(D–H)	d(H...A)	d(D...A)	<(DHA)
**2a** [Fe^{III}(3-OMe-sal$_2$323)]NO$_3$				
N(2)–H(1N2)...O(3)#1	1.00	2.30	3.098	149.0
N(2)–H(1N2)...O(4)#2	1.00	2.19	3.0948(19)	149.2
100 K – #1 x–1,3/2–y,–1/2+z; #2 –x+1/2,y,z–1/2				
N(2)–H(1N2)...O(4)	0.98	2.01	2.9075(16)	150.7
293 K				
**2b** [Fe^{III}(3-OMe-sal$_2$323)]BF$_4$·H$_2$O				
O(5)–H(1O5)...O(1)#1	0.76(3)	2.10(3)	2.8313(16)	163(2)
O(5)–H(2O5)...F(3)	0.81(3)	2.09(3)	2.8706(17)	162(2)
#1 –x+1,y+1/2,–z+3/2				
**2c** [Fe^{III}(3-OMe-sal$_2$323)]PF$_6$·H$_2$O				
O(5)–H(1O5)...O(1)	0.91(3)	2.08(4)	2.864(2)	143(4)
O(5)–H(2O5)...F(3A)	0.93(3)	2.11(5)	2.990(10)	156(6)
**3a** [Fe^{III}(5-OMe-sal$_2$323)]NO$_3$				
N(4)–H(1N4)...O(7)#1	1.00	2.10	2.9987(17)	148.4
#1 x,y+1,z				
**3b** [Fe^{III}(5-OMe-sal$_2$323)]BF$_4$				
N(2)–H(1N2)...F(1)#1	0.78(2)	2.16(3)	2.896(2)	158(2)
#1 –x+1,y,–z+3/2				
**4a** [Fe^{III}(4,6-diOMe-sal$_2$323)]NO$_3$·MeOH				
N(2)–H(1N2)...O(7)#1	1.00	2.17	2.995(4)	139.1
N(3)–H(1N3)...O(7)#2	1.00	2.05	2.988(4)	154.9
100 K – #1 x,–y+3/2,z+1/2; #2 x+1,–y+3/2,z+1/2				
N(2)–H(1N2)...O(7)#1	0.98	2.18	2.998(6)	140.1
N(3)–H(1N3)...O(7)#2	0.98	2.09	2.970(6)	148.8
293 K – #1 x,–y+3/2,z+1/2 #2 x+1,–y+3/2,z+1/2				
**4b** [Fe^{III}(4,6-diOMe-sal$_2$323)]BF$_4$·0.5MeOH				
N(2)–H(1N2)...F(1)	1.00	2.21	3.017(3)	136.5
N(3)–H(1N3)...F(1)#1	1.00	2.10	3.014(3)	151.3
100 K – #1 x+1,y,z				
N(2)–H(1N2)...F(1)	0.98	2.28	3.067(6)	137.0
N(3)–H(1N3)...F(1)#1	0.98	2.16	3.039(6)	147.8
293 K – #1 x+1,y,z				

D–H...A	d(D–H)	d(H...A)	d(D...A)	<(DHA)
**4c** [Fe^{III}(4,6-diOMe-$sal_2$323)]ClO_4·0.5MeOH				
N(3)–H(1N3)...O(7)#1	1.00	2.15	3.050(7)	148.3
#1 x+1,y,z				
**5a** [Fe^{III}(3-OEt-$sal_2$323)]PF_6·EtOH				
N(2)–H(3)...O(5)#1	1.00	2.38	3.209(5)	140.2
N(3)–H(3)...O(5)#1	1.00	2.28	3.257(5)	166.9
#1 –x+1,–y+1,–z				
**5b** [Fe^{III}(3-OEt-$sal_2$323)]BF_4·0.32H_2O				
O(5)–H(2O5)...F(2)#1	0.84	1.80	2.609(7)	161.2
O(5)–H(1O5)...O(3)	0.84	2.17	2.988(6)	164.9
N(3)–H(3)...F(1)#1	1.00	2.22	3.142(2)	153.3
N(2)–H(2)...F(1)	1.00	2.29	3.086(2)	135.3
#1 x+1,y,z				
**6b** [Fe^{III}(4-NEt_2-$sal_2$323)]PF_6				
N(3)–H(3)...F(2)	1.00	2.35	3.174(1)	138.5
N(4)–H(4)...F(1)#1	1.00	2.37	3.331(1)	161.3
#1 x+1,y,z				
**6b·S** [Fe^{III}(4-NEt_2-$sal_2$323)]PF_6·0.78MeCN·0.1EtOH				
N(3)–H(3)...F(1)	1.00	2.15	3.100(3)	157.8
N(4)–H(4)...F(7)#1	1.00	2.44	3.221(3)	134.8
N(9)–H(9)...F(9A)	1.00	2.10	3.097(3)	176.9
N(10)–H(10)...F(5)	1.00	2.30	3.126(2)	139.4
#1 x+1,y,z				
**6c** [Fe^{III}(4-NEt_2-$sal_2$323)]OTf				
N(4)–H(4)...O(5)#1	1.00	2.14	3.0393(15)	149.2
100 K – #1 –x+2,–y+1,–z+1				
N(4)–H(4)...O(4)#1	0.98	2.39	3.180(2)	137.8
293 K – #1 –x+1,–y+1,–z+1				
**6d** [Fe^{III}(4-NEt_2-$sal_2$323)]BF_4				
N(3)–H(1N3)...F(4)#1	1.00	2.20	3.050(2)	142.1
#1 –x+3/2,y–1/2,–z+1/2				
**6d·S** [Fe^{III}(4-NEt_2-$sal_2$323)]BF_4·EtOH				
N(3)–H(1N3)...F(1)	1.00	2.20	3.118(4)	152.3
N(4)–H(1N4)...F(1)#1	1.00	2.08	3.020(4)	154.8
#1 x–1,y,z				
**6e** [Fe^{III}(4-NEt_2-$sal_2$323)]NO_3·CH_2Cl_2				
N(3)–H(1N3)...O(3)#1	1.00	2.05	2.970(7)	152.2
N(4)–H(1N4)...O(3)#2	1.00	2.09	3.035(7)	156.4
#1 x,y+1,z #2 x–1,y+1,z				

D–H...A	d(D–H)	d(H...A)	d(D...A)	<(DHA)
**7a** [Fe^{III}(3-Me-$sal_2$323)]ClO_4				
N(2)–H(1N2)...O(3)#1	1.00	2.11	3.061(4)	158.5
N(3)–H(1N1)...O(4)#2	1.00	2.18	3.049(4)	144.4
#1 x–1,y–1,z; #2 x,y–1,z				
**7b** [Fe^{III}(3-Me-$sal_2$323)]PF_6·0.68H_2O				
N(2)–H(1N2)...F(3)#1	0.87(2)	2.46(2)	3.279(2)	157(2)
#1 x, 1–y, –1/2+z				
**7b** [Fe^{III}(3-Me-$sal_2$323)]BF_4				
N(2)–H(1N2)...F(4)#1	0.85(4)	2.18(4)	2.993(3)	160(4)
N(3)–H(1N3)...F(2)#2	0.93(5)	2.22(5)	3.013(3)	142(4)
#1 –x,y+1/2,–z+1/2; #2 –x+1,y+1/2,–z+1/2				
**8 0982** [Fe^{III}(3-Allyl-$sal_2$323)]NO_3·MeCN				
N(2)–H(1N2)...O(3)	0.93	2.25	3.145(2)	162.2
N(3)–H(1N3)...O(3)#1	0.93	2.18	3.033(2)	151.2
#1 x+1,y,z				
**9a** [Fe^{III}(3-^{t}Bu-$sal_2$323)]PF_6·EtOH				
N(6)–H(6)...O(5A)#1	0.93	2.07	2.930(6)	152.7
N(6)–H(6)...O(5B)#1	0.93	2.07	2.749(18)	128.8
O(5A)–H(5A)...F(1)	0.84	2.00	2.838(5)	172.0
O(5B)–H(5B)...F(1)	0.84	1.92	2.711(17)	156.1
N(2)–H(2)...F(5)	0.93	2.44	3.227(3)	142.1
N(7)–H(7)...F(3)	0.93	2.36	3.229(4)	155.3
N(7)–H(7)...F(6)	0.93	2.54	3.204(4)	128.1
#1 –x,–y+1,–z				
**9b** [Fe^{III}(3-^{t}Bu-$sal_2$323)]BF_4				
N(2)–H(1N2)...F(2)#1	0.91	2.07	2.913(9)	154.6
N(3)–H(1N3)...F(1)#2	0.91	2.07	2.925(9)	155.3
#1 x–1,y+1,z; #2 x–1,y,z				
**10e** [Fe^{III}($sal_2$323)]$FeCl_4$				
N(2)–H(2)...Cl(3B)#1	0.93	2.55	3.365(7)	147.3
N(3)–H(3)...Cl(3B)#2	0.93	2.41	3.287(6)	157.2
N(2)–H(2)...Cl(3A)#1	0.93	2.54	3.398(2)	154.2
N(3)–H(3)...Cl(3A)#2	0.93	2.47	3.304(2)	148.9
#1 –x+1,y+1/2,–z+1/2; #2 –x,y+1/2,–z+1/2				
**10f** [Fe^{III}($sal_2$323)]BF_4				
N(3)–H(3)...F(3A)#1	1.00	2.13	2.935(2)	136.4
N(2)–H(2)...F(3A)#2	1.00	2.26	3.069(2)	136.9
#1 –x,–y+1,–z+1; #2 –x+1,–y+1,–z+1				

D–H...A	d(D–H)	d(H...A)	d(D...A)	<(DHA)
**11a** [Fe^{III}(5-Br-sal$_2$323)]PF_6				
N(2)–H(1N2)...Br(2)#1	0.91	2.99	3.784(3)	146.0
N(3)–H(1N3)...Br(2)#2	0.91	2.95	3.635(3)	133.8
#1 –x, –1/2+y,1–z; #2 1–x, –1/2+y,1–z				
**11b** [Fe^{III}(5-Br-sal$_2$323)]BF_4·EtOH				
O(3)–H(1O3)...F(3)#1	0.82	2.12	2.902(4)	158.6
N(3)–H(1N3)...O(3)	0.83(4)	2.08(4)	2.899(4)	168(3)
N(2)–H(1N2)...F(3)#2	0.82(4)	2.24(3)	3.021(4)	159(3)
#1 x,y–1,z #2 1+x, –1+y,z				
**11c** [Fe^{III}(5-Br-sal$_2$323)]NO_3·^{i}PrOH				
N(2)–H(1N2)...O(9)#1	0.84(3)	2.20(4)	2.980(3)	155(3)
N(6)–H(1N6)...O(7)	0.85(4)	2.16(4)	2.978(3)	163(3)
O(11)–H(11O)...O(10)	0.78(4)	2.03(4)	2.812(3)	179(4)
O(12)–H(12O)...O(5)#2	0.83(5)	2.00(5)	2.827(4)	173(4)
N(3)–H(1N3)...O(10)#1	0.84(3)	2.36(3)	3.036(3)	138(3)
N(7)–H(1N7)...O(5)#3	0.82(4)	2.41(4)	3.067(3)	138(3)
#1 –x,–y,–z+1; #2 –x+1,–y+1,–z+1; #3 x,1+y,z				
**12** [Fe^{III}(3,5-diBr-sal$_2$323)]NO_3·^{i}PrOH				
O(6B)–H(6B)...O(3)#1	0.84	2.18	2.879(5)	140.7
O(6A)–H(6A)...O(3)#1	0.84	2.04	2.879(5)	175.8
N(3)–H(1N3)...O(5)	0.82(6)	2.17(6)	2.961(5)	161(5)
N(2)–H(1N2)...O(4)#2	0.77(6)	2.33(6)	3.064(5)	159(6)
#1 –x+1/2,y+1/2,–z+3/2; #2 3/2–x, –1/2+y,3/2–z				
**13a** [Fe^{III}(3,5-diCl-sal$_2$323)]BF_4·^{i}PrOH				
O(3)–H(1O3)...F(1)#1	0.82	1.99	2.801(3)	167.3
N(4)–H(1N4)...O(3)	0.88(3)	2.15(3)	2.933(3)	148(2)
N(2)–H(1N2)...F(2)#2	0.81(3)	2.17(3)	2.863(2)	144(2)
#1 –x+1,–y,–z+1; #2 x+1/2,–y+1,z–1/2				
**13b** [Fe^{III}(3,5-diCl-sal$_2$323)]PF_6				
N(3)–H(1N3)...F(4)	0.82(3)	2.50(3)	3.269(3)	157(3)
N(3)–H(1N3)...F(4)	0.82(3)	2.50(3)	3.269(3)	157(3)
N(2)–H(1N2)...F(5)#1	0.80(3)	2.49(3)	3.263(3)	163(3)
#1 1/2+x,1/2–y, –1/2+z				
**14** [Fe^{III}(3,5-diI-sal$_2$323)]PF_6				
N(2)–H(1N2)...I(3)#1	0.93	3.11	3.989(3)	157.9
N(3)–H(1N3)...I(3)#2	0.93	3.08	3.947(3)	156.4
#1 x–1,y,z; #2 –x,y–1/2,–z+1/2				

D–H...A	d(D–H)	d(H...A)	d(D...A)	<(DHA)
**15a** [Fe^{III}(3-NO_2-sal$_2$323)]PF_6·MeCN				
N(4)–H(1N4)...O(6)#1	0.81(3)	2.25(3)	3.000(3)	155(3)
#1 –x+2,–y+1,–z+2				
**15b** [Fe^{III}(3-NO_2-sal$_2$323)]NO_3				
N(3)–H(1N3)...O(7)	0.93	2.05	2.927(3)	157.8
N(4)–H(1N4)...O(9)#1	0.93	2.00	2.922(3)	172.5
#1 x–1/2,y+1/2,z				
**16a** [Fe^{III}(5-NO_2-sal$_2$323)]PF_6·EtOH				
N(3)–H(3)...O(7)#1	0.77(3)	2.24(3)	2.995(3)	167(3)
O(7)–H(7)...F(5A)#2	0.82	2.33	3.117(6)	160.2
N(4)–H(4)...F(5A)#1	0.81(3)	2.42(3)	3.209(5)	166(2)
#1 x+1,y,z; #2 x,y–1,z; #3 x,1+y,z				
**16b** [Fe^{III}(5-NO_2-sal$_2$323)]BF_4·EtOH				
O(7)–H(7)...F(3)	0.82	2.16	2.945(7)	161.6
N(3)–H(3)...O(7)#1	0.91	2.03	2.933(6)	171.4
N(4)–H(4)...F(3)	0.91	2.12	3.010(6)	164.1
#1 x+1,y,z				
**16c** [Fe^{III}(5-NO_2-sal$_2$323)]ClO_4·EtOH				
N(3)–H(1N3)...O(11)	1.00	1.91	2.905(3)	170.5
N(4)–H(1N4)...O(7)#1	1.00	2.03	3.021(3)	172.6
O(11)–H(11)...O(7)#2	0.84	2.05	2.860(3)	163.0
#1 x–1,y+1,z; #2 x,y+1,z				
**17** [Fe^{III}(3,5-diNO_2-sal$_2$323)]ClO_4·EtOH				
O(15)–H(15)..O(13)#1	0.82(3)	2.04(3)	2.8485(19)	171(2)
N(5)–H(5)...O(15)#2	0.87(2)	2.03(2)	2.8496(17)	156(2)
N(4)–H(4)...O(12)#	0.85(2)	2.44(2)	3.035(2)	128(2)
N(4)–H(4)...O(6)#	0.85(2)	2.55(2)	3.283(2)	146(2)
#1 –x+1,–y+1,–z+1; #2 x+1/2,–y+1/2,z+1/2; #3 x–1/2, –y+1/2,z–1/2				

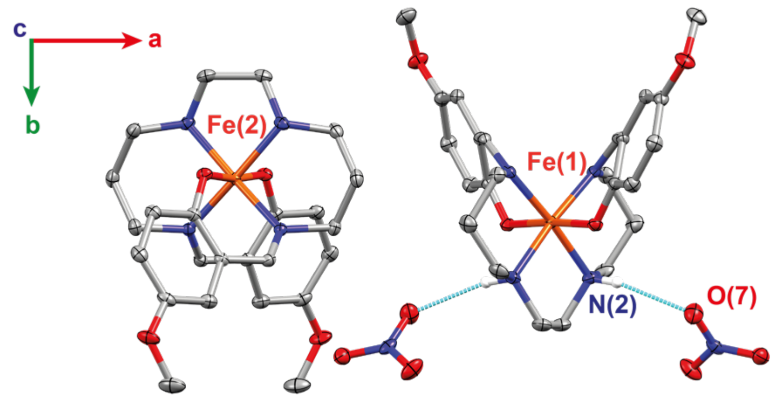

Figure S2.1. Molecular structure of **3a** showing the two unique cations, and the N–H…O hydrogen bonding existing between the amine N(2) and O(7) of the NO_3^- anion. Thermal ellipsoids are drawn at 50 % probability. Hydrogen atoms, except those involved in hydrogen bonding have been omitted for clarity. A similar motif is observed in **3b**.

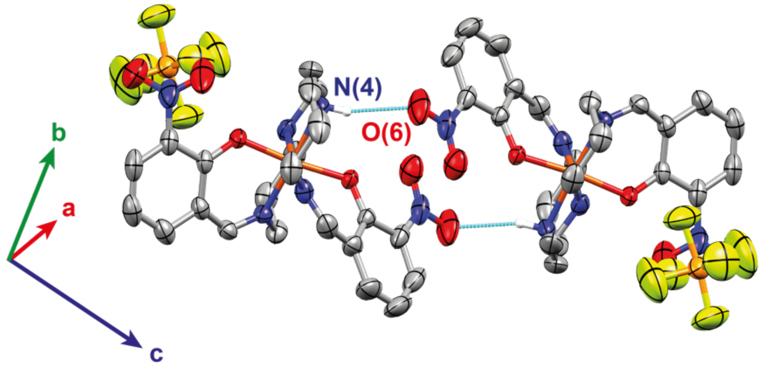

Figure S2.2. Partial packing diagram showing the formations of hydrogen bonding dimers through N–H…O interactions. Thermal ellipsoids are drawn at 50 % probability. Hydrogen atoms, except those involved in hydrogen bonding have been omitted for clarity.

Table S2.4 Summary of bond lengths and distortion parameters of high spin Fe^{III} cations with similar hexadentate ligands of differing backbone lengths.[7-9]

**Complex**	[Fe(5-Me-sal$_2$222)]PF_6	[Fe(4,6-diOMe-sal$_2$232)]ClO_4	[Fe(5-F-sal$_2$333)]Cl
**Refcode**	WOBWOA	ELANOV	FONFAS
**Bond Length (Å)**			
**Fe–O$_{phen}$**	1.9315(13)	1.906(2)	1.9426(9)
**Fe–N$_{imine}$**	2.1245(14)	2.045(3)	2.1398(11)
**Fe–N$_{amine}$**	2.2179(15)	2.144(3)	2.1837(11)
**Distortion Parameters (°)**			
Σ	93.87	74.62	57.97
Θ	325.88	301.84	230.34

S2.4 Hirshfeld Surface Analysis

Table S2.5. Relative contribution (%) of the various intermolecular interactions to the Hirshfeld surface in **1a** – **1h**.

Complex	1a		1a·S		1b	1c	1d	1e	1f	1g	1h
***T* (K)**	100	293	100	293	100	100	100	100	100	100	100
**H···H**	54.5	56.7	55.2	55.8	48	44.2	53.2	52.6	46.6	55.9	65.2
**O···H**	24	21.9	19.4	19.2	12.2	25.5	26.4	9.5	11.3	8.6	10.4
**C···H**	18.9	18.5	18.2	17.9	15.9	14.4	18.3	17.5	15.8	15.9	16.2
**O···O**	1.3	1.3	0.1	0.2	0.2	0.7	0.4	0	0.2	0	0.1
**N···H**	0.8	1	5.6	5.6	0.4	0.3	0.1	0.3	0.4	0	0.1
**C···C**	0.1	0.2	0.7	0.7	1.1	1.2	0.1	0	0.8	0.7	0
**C···O**	0.4	0.5	0.8	0.6	1.1	1.9	1.4	1.3	0.8	1.1	0
**F···H**	0	0	0	0	21.2	11.6	0	18.8	23.9	0	0
**I···H**	0	0	0	0	0	0	0	0	0	17.1	0
**O···I**	0	0	0	0	0	0	0	0	0	0.8	0
**Cl···H**	0	0	0	0	0	0	0	0	0	0	8

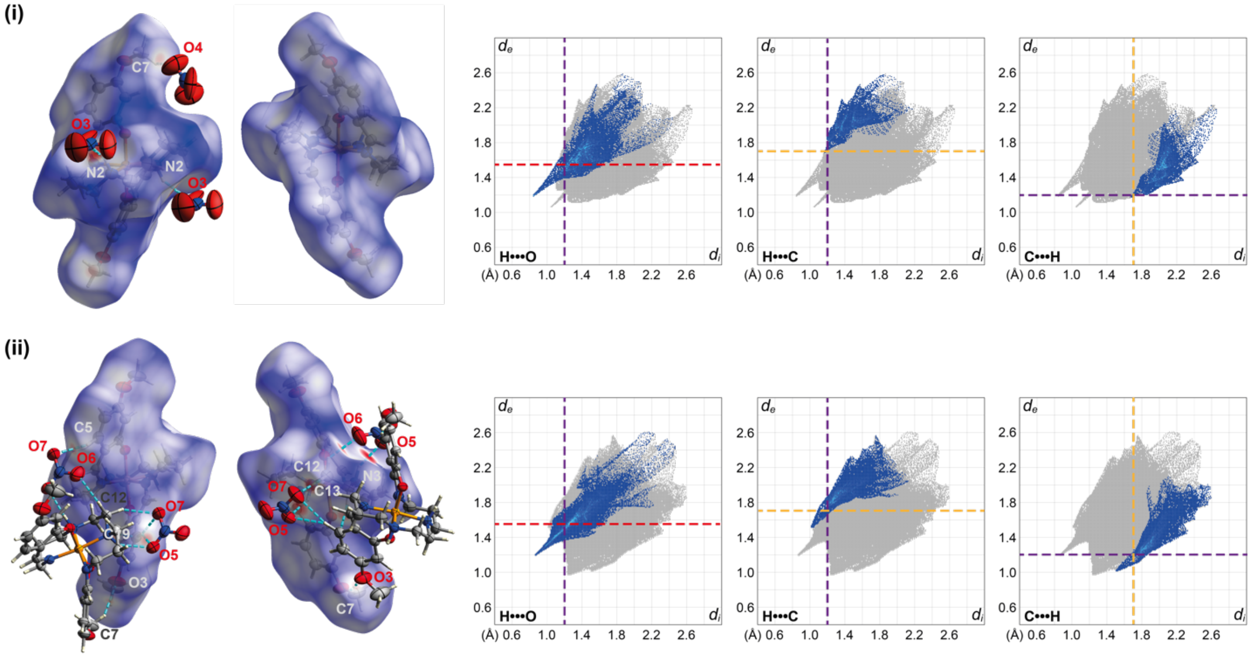

Figure S2.3. Hirshfeld surface analysis for **1a (i)** and **1a·S (ii)** at 293 K with two views of the cation for both. The surface is mapped with d_{norm} values of -0.4374 to 1.3143 a.u. **(i)** and -0.4014 to 1.3143 a.u. **(ii)**. Contacts which are shorter than the van der Waals (vdW) radii of the contact atoms are shown in red on the surface and contacts longer than the vdW radii in blue. Contacts which are shorter than the vdW radii are indicated with a dashed blue line and labelled according to the contact atoms inside (white) and outside the surface (colored). Two-dimensional fingerprint plots delineated into H···O, H···C and C···H contacts, with d_i on the *x*-axis (distance from the surface to the closest internal atom) and d_e on the *y*-axis (distance from the surface to the closest external atom). The dashed lines on the plots refer to the vdW radii of the selected atoms:[10]

S3 Magnetic Measurements

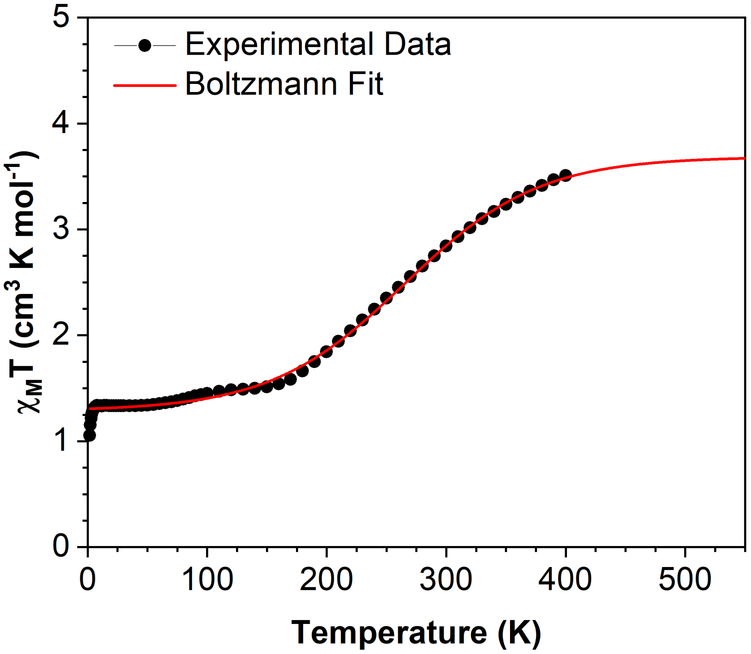

Figure S3.1. Magnetic susceptibility of **1a** (black circles) and fitted to the Boltzmann equation (red line), which reveals a $T_{1/2}$ of 265 K.

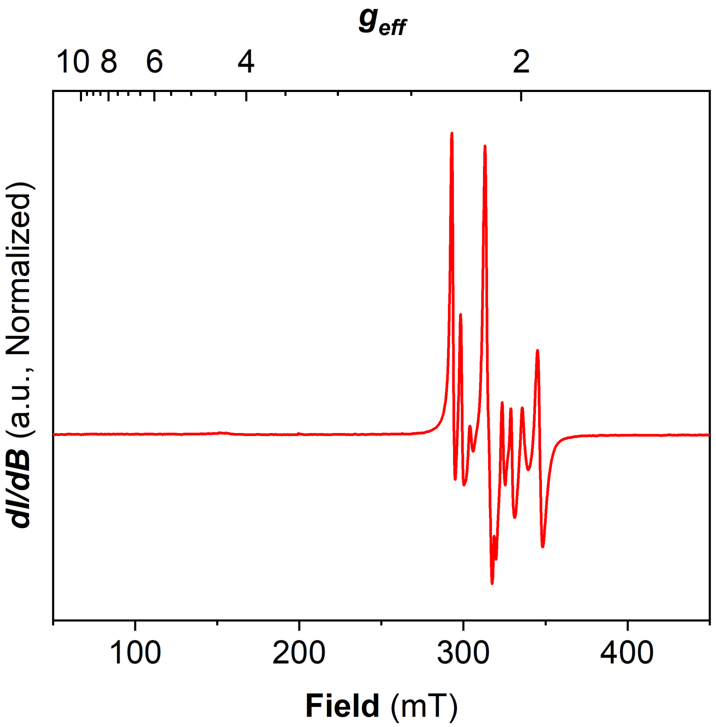

Figure S3.2. EPR spectrum of a polycrystalline sample of **1a**. The multi-line spectrum was obtained as the sample was polycrystalline and not ground.

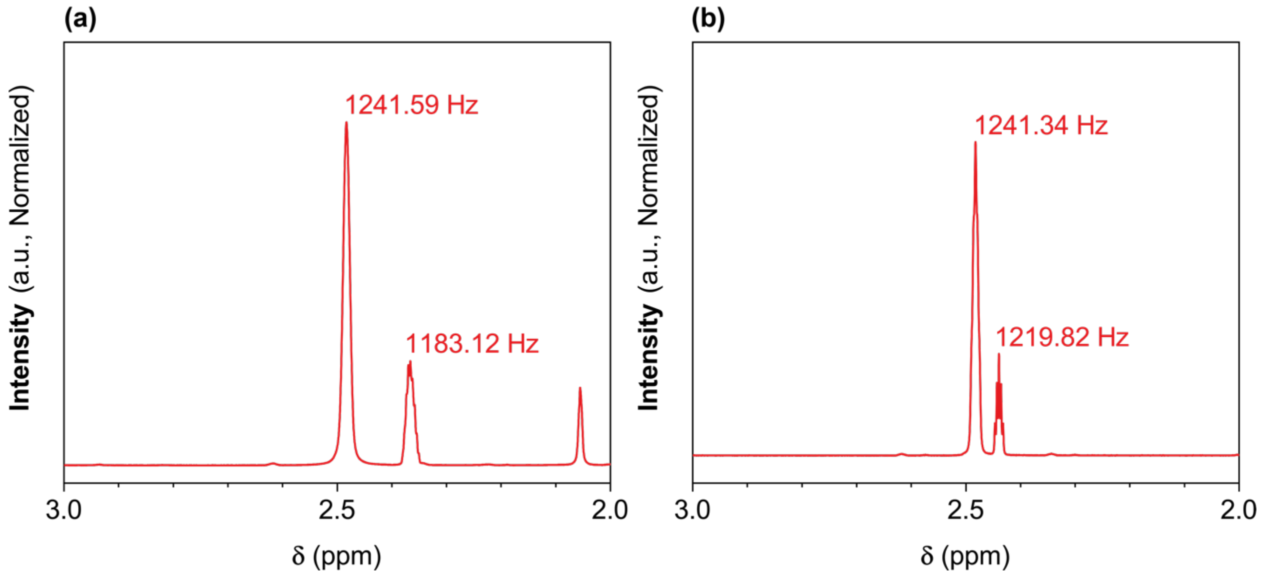

Figure S3.3. ^{1}H NMR spectra of **1a** (a) and **1d** (b) showing the shift in the DMSO-d_6 peaks. The peaks are labelled with their chemical shift in Hz.

S4 Quantum Chemistry Calculations

Table S4.1. XYZ coordinates of the optimized geometries of the $[Fe^{III}(sal_2 323)]^+$ and $[Fe^{III}(4\text{-OMe-}sal_2 323)]^+$ cations.

$[Fe^{III}(sal_2 323)]^+$							
Spin Sextet (S = 5/2, HS)				Spin Doublet (S = 1/2, LS)			
Fe	0.753843003	11.35305228	11.89688577	Fe	0.754629586	11.36497342	11.83157739
O	1.564473465	9.594799204	11.72597859	O	1.714348933	9.801990325	11.41333129
C	2.231623391	8.872033774	12.60107277	C	2.235046663	9.004601609	12.331942
C	2.250860061	7.463809671	12.48490282	C	2.472918117	7.650576837	12.00626178
H	1.71580756	7.009617282	11.65021699	H	2.228810088	7.314639728	10.99791359
C	2.935033898	6.682765715	13.40762414	C	2.982328274	6.772241674	12.95234917
H	2.926869141	5.597111369	13.30049656	H	3.135310184	5.726160248	12.68358647
C	3.641279985	7.271286844	14.47147869	C	3.307189359	7.213882943	14.24872211
H	4.177398339	6.649168254	15.18679532	H	3.711000665	6.516454476	14.98138754
C	3.659161845	8.651969399	14.58918877	C	3.133828903	8.548500169	14.57170333
H	4.219855565	9.126726498	15.39736935	H	3.421918128	8.917121783	15.55836827
C	2.960446988	9.475171379	13.67796588	C	2.590163412	9.463470334	13.64012965
C	3.087241479	10.8994208	13.80283583	C	2.598154405	10.8644539	13.93720682
H	3.84050322	11.24937559	14.52895608	H	3.24332121	11.18924058	14.76539929
N	2.433772564	11.79420145	13.12353609	N	1.991818937	11.78798387	13.24471196
C	2.852935303	13.18944081	13.23266232	C	2.453456402	13.17027604	13.41395186
H	3.617295364	13.29593639	14.02100717	H	3.080219908	13.23646519	14.31648182
H	1.983485076	13.80172168	13.50947366	H	1.593540958	13.83752998	13.54874976
C	3.427521662	13.68190653	11.89630207	C	3.26819453	13.59847523	12.18735557
H	3.840041564	14.68972452	12.05438374	H	3.689759732	14.59592796	12.38173086
H	4.278206144	13.04062856	11.60983666	H	4.123268026	12.91447215	12.05835467
C	2.428642999	13.7736193	10.74534538	C	2.453486654	13.68400967	10.90177418
H	1.560090753	14.37713799	11.04361723	H	1.593788011	14.35190596	11.04591414
H	2.911609519	14.27107637	9.885572249	H	3.073798111	14.09742974	10.0887822
N	1.899601938	12.45458575	10.3188205	N	1.898252343	12.3745516	10.47253674
H	2.686677681	11.82146821	10.13509681	H	2.667517511	11.71405756	10.30433541
C	1.059159656	12.54403566	9.107508639	C	1.08978286	12.50525381	9.234666718
H	1.645965177	12.83722213	8.220704057	H	1.717587509	12.76071181	8.366179384
H	0.313804719	13.33441334	9.285947167	H	0.384486357	13.33215721	9.400663137
C	0.368192837	11.21013882	8.877108093	C	0.343033081	11.20676005	9.009969884
H	-0.242936427	11.2410689	7.959353587	H	-0.309328395	11.26900413	8.124401745
H	1.114337489	10.41065252	8.750155708	H	1.048254413	10.37740387	8.856343517
N	-0.439888089	10.8681851	10.06537779	N	-0.431004559	10.89136194	10.23657284
H	-1.227783354	11.52275807	10.13504689	H	-1.200686061	11.56576881	10.33197718
C	-0.965491777	9.481363169	10.01767052	C	-0.981777752	9.512272947	10.19379732
H	-0.092740554	8.814917454	10.05952403	H	-0.12215229	8.840186819	10.0696476
H	-1.476075397	9.315266363	9.052439499	H	-1.626624408	9.408199856	9.304966501
C	-1.930516727	9.159384917	11.15605398	C	-1.760206275	9.137144274	11.44957222

H	-2.344612319	8.158451828	10.96234843	H	-2.182016034	8.133200508	11.29275802
H	-2.784989733	9.856845027	11.13755095	H	-2.614484114	9.819222133	11.59313916
C	-1.315127929	9.154227782	12.56316926	C	-0.909469408	9.110602189	12.72523329
H	-2.057088388	8.773825902	13.28555761	H	-1.511250743	8.728562594	13.56406961
H	-0.441199536	8.488399511	12.58233042	H	-0.049940884	8.442632467	12.59258231
N	-0.892210252	10.5012744	12.93849056	N	-0.445385034	10.46651982	13.03957252
C	-1.522136572	11.09798785	13.90607707	C	-1.029200843	11.0853275	14.02776073
H	-2.256401309	10.51191601	14.48444572	H	-1.653244719	10.48711098	14.70623469
C	-1.392210485	12.47647571	14.28428744	C	-1.024135303	12.50159225	14.24049503
C	-2.061030807	12.92474847	15.44528701	C	-1.540693345	13.02886964	15.44705575
H	-2.600825577	12.19403885	16.05158706	H	-1.804225873	12.33620883	16.24902125
C	-2.040042041	14.25936917	15.81785403	C	-1.71874722	14.39091543	15.61646892
H	-2.552813859	14.58879032	16.72044902	H	-2.10162886	14.78494161	16.55689894
C	-1.361475922	15.18694482	15.0078657	C	-1.426454154	15.26073824	14.54916431
H	-1.351318779	16.24145191	15.28733202	H	-1.583560849	16.33366939	14.66774539
C	-0.707334816	14.78210892	13.85125276	C	-0.944353509	14.77267787	13.34277456
H	-0.194040528	15.50267499	13.21361595	H	-0.72599112	15.44204533	12.51001449
C	-0.691350205	13.42243196	13.46661269	C	-0.702179132	13.39189123	13.1673386
O	-0.052574001	13.05535652	12.37551193	O	-0.208942386	12.97013749	12.01428156
**[Fe^{III}(4-OMe-sal$_2$323)]$^+$**							
**Spin Sextet (S = 5/2, HS)**				**Spin Doublet (S = 1/2, LS)**			
Fe	-1.095538255	5.351638493	16.57906009	Fe	-1.099611924	5.370314373	16.61853123
O	-2.506513973	6.483064688	16.04166989	O	-2.467439498	6.515614925	15.81167197
C	-3.084986733	6.402125567	14.85581829	C	-3.167015288	6.328411195	14.71339884
C	-3.732873252	7.547033755	14.34628164	C	-3.658245382	7.446283819	14.00764237
H	-3.7285427	8.445605451	14.95980018	H	-3.447411206	8.433070489	14.41436832
C	-4.340294883	7.514027552	13.08918413	C	-4.395145506	7.275124487	12.83180158
O	-4.958670596	8.569274533	12.51364267	O	-4.889286276	8.290116991	12.08987535
C	-5.029789274	9.79795049	13.24787185	C	-4.668054309	9.63371932	12.5390101
H	-5.572487481	9.659041252	14.19555437	H	-5.119126611	9.7961095	13.53007395
H	-5.578876655	10.49327413	12.60596219	H	-5.158048093	10.27532644	11.80048079
H	-4.02244983	10.19517699	13.44830359	H	-3.591913494	9.864176935	12.57434281
C	-4.338569277	6.323913796	12.31815609	C	-4.675812863	5.976799279	12.33841532
H	-4.827210436	6.329680135	11.3451647	H	-5.256900934	5.877814118	11.42310561
C	-3.748664184	5.19224566	12.83013427	C	-4.222332806	4.881339352	13.03634298
H	-3.777557581	4.262865256	12.25806962	H	-4.451376089	3.87790421	12.6716635
C	-3.099490802	5.19134094	14.0915383	C	-3.456990487	5.011296057	14.22268335
C	-2.670524029	3.961384999	14.668313	C	-3.09430955	3.835768162	14.9433031
H	-3.057638957	3.039558188	14.21165632	H	-3.555413948	2.897016061	14.5924488
N	-1.938558362	3.83927948	15.74514971	N	-2.307073995	3.773319373	15.98289848
C	-1.972218233	2.5504948	16.44130208	C	-2.247898324	2.522755918	16.73341192
H	-0.952283387	2.230839441	16.68980239	H	-1.198715163	2.207882917	16.82701749
H	-2.413742777	1.789846651	15.77874149	H	-2.798556645	1.731277489	16.19653982

C	-2.81168853	2.670071398	17.71927361	C	-2.857828635	2.7064658	18.13153773
H	-3.830531255	3.001218429	17.45825471	H	-3.900119682	3.052658304	18.02831315
H	-2.911221264	1.670371825	18.16821188	H	-2.90669473	1.719812596	18.61682391
C	-2.205072724	3.59117292	18.77198225	C	-2.087059625	3.635557411	19.06717732
H	-2.798720369	3.547215068	19.70093025	H	-2.527132722	3.578867811	20.07889757
H	-1.181997806	3.268107242	19.00734423	H	-1.037105739	3.317978927	19.13485504
N	-2.102949921	5.003407163	18.32427899	N	-2.069943466	5.046999496	18.61281042
H	-3.040602943	5.362851929	18.10565456	H	-3.032534782	5.355430987	18.43379337
C	-1.481183523	5.861788039	19.36208344	C	-1.441215686	5.956364171	19.59081295
H	-0.556967175	5.357886342	19.67984496	H	-0.467433343	5.519772276	19.86151128
H	-2.132116222	5.969029674	20.24475164	H	-2.034429867	6.04052857	20.51730188
C	-1.165184757	7.209711948	18.74636114	C	-1.24597675	7.323691331	18.9545642
H	-2.088457995	7.707182689	18.4158963	H	-2.21571787	7.739099949	18.6397358
H	-0.661775201	7.870978801	19.46996634	H	-0.803800482	8.029135899	19.67851155
N	-0.337142318	6.992660334	17.53484064	N	-0.416634169	7.184957	17.74138648
H	0.59499566	6.66441411	17.81697184	H	0.537910538	6.921645902	18.01204371
C	-0.204283587	8.235810412	16.73356774	C	-0.368627035	8.426283412	16.93163415
H	0.245308231	9.024912971	17.36004886	H	-0.082159293	9.276473917	17.57620969
H	-1.225900174	8.546983108	16.47676937	H	-1.391921644	8.602216636	16.57149719
C	0.615998722	8.045965751	15.4624164	C	0.597205444	8.343380125	15.75141002
H	1.637086617	7.71075712	15.70903272	H	1.615803172	8.120086539	16.11107437
H	0.716071914	9.029945448	14.9800935	H	0.643093489	9.343669942	15.29460706
C	-0.011351865	7.071302046	14.45800654	C	0.21766804	7.33410241	14.65712052
H	-1.031659893	7.390756493	14.21088458	H	-0.808579093	7.529756082	14.31486093
H	0.577714851	7.069592409	13.52767379	H	0.897217643	7.456804888	13.79636439
N	-0.031736626	5.718896074	15.0214583	N	0.29906174	5.970287305	15.17099404
C	0.842703623	4.86176157	14.56213369	C	1.226590723	5.183448767	14.69662077
H	1.358611057	5.117042363	13.62574659	H	1.800651487	5.536457667	13.82324272
C	1.264571628	3.671993475	15.22243281	C	1.609028146	3.912141697	15.21635737
C	2.094008498	2.742596615	14.54358761	C	2.551820531	3.132324587	14.49989741
H	2.274646405	2.88738065	13.47670214	H	2.90389202	3.498925554	13.53341971
C	2.672011924	1.676689733	15.19209688	C	3.027012351	1.936220954	14.98562921
H	3.299693119	0.955902089	14.67056702	H	3.745498486	1.333389197	14.43260215
C	2.473314831	1.523880254	16.58782252	C	2.5839721	1.479975714	16.25193536
O	3.094818611	0.459308909	17.1424419	O	3.112372464	0.304081283	16.6548098
C	2.968346966	0.259599976	18.55592433	C	2.732880967	-0.209222355	17.93854945
H	3.370773708	1.120078635	19.11228785	H	3.016452057	0.489463447	18.7404657
H	3.557560478	-0.634384611	18.78095174	H	3.283169675	-1.147614229	18.05429572
H	1.916987043	0.091007203	18.83682323	H	1.650436868	-0.406785423	17.97874665
C	1.683119971	2.431466322	17.29643431	C	1.667381953	2.22738166	16.99788378
H	1.523117298	2.34032263	18.36879117	H	1.32813504	1.904974218	17.98016626
C	1.04935845	3.499677655	16.62752917	C	1.155652025	3.441123719	16.49403262
O	0.295807198	4.32451054	17.33395956	O	0.287287044	4.121730444	17.21047692

Table S4.2. Calculated Gibbs free energies (ΔG_{SCO}) upon SCO from the LS to HS state for the $[Fe^{III}(sal_2323)]^+$ and $[Fe^{III}(4\text{-OMe-}sal_2323)]^+$ cations.

	**$[Fe^{III}(sal_2323)]^+$**	**$[Fe^{III}(4\text{-OMe-}sal_2323)]^+$**
	**kJ mol^{-1}**	
ΔE	+40.7	+40.2
ΔG_{corr}	−21.9	−21.8
ΔG_{SCO} (HS-LS)	+18.8	+18.4

S5 Powder X-ray Diffraction

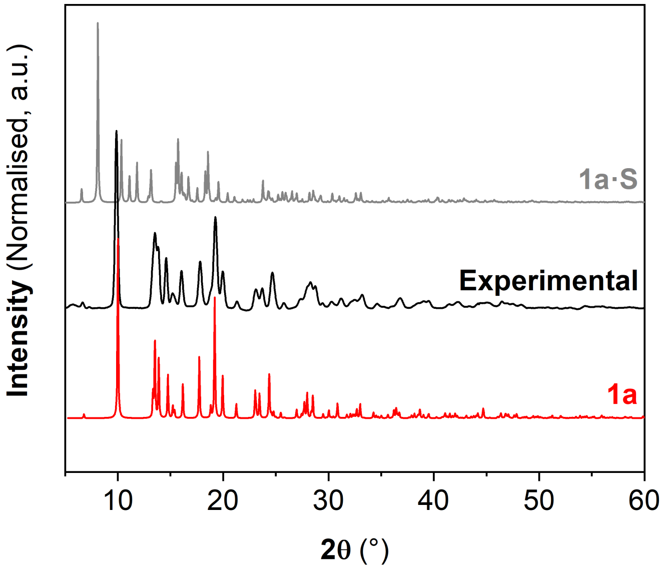

Figure S5.1 Micro-powder XRD spectrum of **1a**/**1a·S** recorded on the sample used in the magnetic susceptibility measurement. The simulated spectra for **1a** and **1a·S** from the single crystal XRD data are provided in red and grey respectively.

S6 Author Contribution

Conor T. Kelly: Conceptualization, Formal Analysis, Investigation, Data Curation, Writing – Original Draft, Writing – Review & Editing, Visualization. **Michael Griffin:** Conceptualization, Formal Analysis, Investigation. **Kane Esien:** Investigation**. Solveig Felton:** Resources, Supervision. **Helge Müller-Bunz:** Formal Analysis, Investigation. **Grace G. Morgan:** Conceptualization, Resources, Writing – Review & Editing, Supervision, Project Administration, Funding Acquisition.

S6 References

1. Tooke, D. M.; Spek, A. L.; Ramu, K.; Reedijk, J., CCDC 808899: Experimental Crystal Structure Determination. *CSD Communication* **2011**.

2. Kannappan, R.; Tanase, S.; Mutikainen, I.; Turpeinen, U.; Reedijk, J., Low-spin iron(III) Schiff-base complexes with symmetric hexadentate ligands: Synthesis, crystal structure, spectroscopic and magnetic properties. *Polyhedron* **2006,** *25* (7), 1646-1654.

3. Howard-Smith, K. J.; Craze, A. R.; Badbhade, M.; Marjo, C. E.; Murphy, T. D.; Castignolles, P.; Wuhrer, R.; Li, F., Syntheses and Structure Investigations of 3d Transition Metal Complexes with a Flexible N_4O_2-Donor Hexadentate Schiff-Base Ligand. *Aust. J. Chem.* **2017,** *70* (5), 581-587.

4. Ito, T.; Sugimoto, M.; Ito, H.; Toriumi, K.; Nakayama, H.; Mori, W.; Sekizaki, M., A chelate ring size effect of spin states of iron(III) complexes with hexadentate ligands derived from salicylaldehyde and 4,8-diazaundecance-1,11-diamine(3,3,3-tet) or 4,7-diazadecance-1,10-diamine(3,2,3-tet), and their X-ray structures *Chem. Lett.* **1983,** *12* (1), 121-124.

5. Hayami, S.; Matoba, T.; Nomiyama, S.; Kojima, T.; Osaki, S.; Maeda, Y., Structures and Magnetic Properties of Some Fe(III) Complexes with Hexadentate Ligands: in Connection with Spin-Crossover Behavior. *Bull. Chem. Soc. Jpn.* **1997,** *70* (12), 3001-3009.

6. Butcher, R. J.; Pourian, M.; Jasinski, J. P., [2,2′-(2,6,9,13-Tetraazatetradeca-1,13-diene-1,14-diyl)diphenolato]iron(III) chloride. *Acta Crystallogr., Sect. E: Struct. Rep. Online* **2007,** *63* (11), m2742-m2743.

7. Pritchard, R.; Barrett, S. A.; Kilner, C. A.; Halcrow, M. A., The influence of ligand conformation on the thermal spin transitions in iron(III) saltrien complexes. *Dalton Trans.* **2008,** (24), 3159-3168.

8. Sundaresan, S.; Kühne, I. A.; Kelly, C. T.; Barker, A.; Salley, D.; Müller-Bunz, H.; Powell, A. K.; Morgan, G. G., Anion Influence on Spin State in Two Novel Fe(III) Compounds: [Fe(5F-$sal_2$333)]X. *Crystals* **2019,** *9* (1), 19.

9. Griffin, M.; Shakespeare, S.; Shepherd, H. J.; Harding, C. J.; Letard, J. F.; Desplanches, C.; Goeta, A. E.; Howard, J. A.; Powell, A. K.; Mereacre, V.; Garcia, Y.; Naik, A. D.; Muller-Bunz, H.; Morgan, G. G., A symmetry-breaking spin-state transition in iron(III). *Angew. Chem. Int. Ed.* **2011,** *50* (4), 896-900.

10. Batsanov, S. S., Van der Waals Radii of Elements. *Inorg. Mater.* **2001,** *37* (9), 871-885.
